# Ultra-deep multi-oncopanel sequencing of benchmarking samples with a wide range of variant allele frequencies

**DOI:** 10.1038/s41597-022-01359-6

**Published:** 2022-06-09

**Authors:** Binsheng Gong, Rebecca Kusko, Wendell Jones, Weida Tong, Joshua Xu

**Affiliations:** 1 Division of Bioinformatics and Biostatistics, National Center for Toxicological Research, US Food and Drug Administration, Jefferson, AR 72079 USA; 2 Immuneering Corporation, One Broadway, 14th Floor, Cambridge, MA 02142 USA; 3 Q2 Solutions - EA Genomics, 5927 S Miami Blvd., Morrisville, NC 27560 USA

**Keywords:** Next-generation sequencing, Data publication and archiving

## Abstract

The lack of suitable reference genomic material to enable a transparent cross-lab study of oncopanels inspired the SEQC2 Oncopanel Sequencing Working Group to develop four reference samples, sequenced with eight oncopanels at independent test laboratories with ultra-deep sequencing depth. This rich, publicly available dataset enabled performance assessment of the clinical applicability of oncopanels. In addition, this dataset present sample opportunities for developing specific and robust bioinformatics pipelines and fine-tuning parameters for more accurate variant calling, investigating ideal sequencing depth for variant calling of a given minimum VAF and variant type, and also recommending best use cases for Unique Molecular Identifier (UMI) technology.

## Abstract (summary)

**Table:** 

Measurement(s)	Short Somatic Mutation
Technology Type(s)	Tumor Panel Sequencing
Factor Type(s)	Tumor Panel • Genomic Region
Sample Characteristic - Organism	Homo sapiens

## Background & Summary

Personalized therapy, diagnostics and prognostic prediction for cancer patients are critical components for bringing the precision medicine era from theory to practice. The advantage of emerging technologies, including the next generation sequencing, oncopanel testing, as well as the bioinformatics tools, are bringing hope to the patients and their caregivers. Physicians can guide patients to targeted therapy rather than general treatment when corresponding genomic indicators are accurately identified with these technologies. False positive variant calls from these technologies can lead to unnecessary or improper therapies as well as overestimation of biomarkers such as tumor mutational burden (TMB), a key measurement for immune checkpoint inhibitor (ICI) therapy. In recent years, the FDA has approved or cleared several cancer panels which are designed to provide physicians with clinically actionable information related to specific targets, a significant milestone for the precision medicine field. Oncopanels typically cover a small-to-large number (50–1000) of genes and can detect mutations with variant allele frequency (VAF) as low as 1% if given sufficient sequencing depth and base quality. Most pan-cancer panels are currently still for research use only. Before they can be adopted for clinical testing, a thorough understanding of their robustness, analytical characteristics, reproducibility, sensitivity, and false positive rate is greatly needed.

We proposed a multi-lab cross-oncopanel study to assess the performance and the clinical applicability of oncopanels^1^. Eight panel providers agreed to participant in this study: Agilent Technologies, Burning Rock Biotech, Integrated DNA Technologies, Inc., iGeneTech, Illumina Inc., QIAGEN Sciences Inc., Roche Sequencing Solutions Inc., and Thermo Fisher Scientific. In order to evaluate the performance of oncopanels in a comprehensive and fair manner, we formed the Oncopanel Sequencing Working Group under the umbrella of the Sequencing Quality Control Phase II (SEQC2) consortium. The working group contained 151 members from 100 institutes, government agencies and private companies across 14 countries. The working group mainly focused on assessing the oncopanel performance using three key metrics: sensitivity, false positive rate, and reproducibility. We developed four related reference samples with a large number of high-confident small variant positive and negatives positions to serve as known content for the performance assessment^2^. The four reference samples were distributed to the eight participating panel providers identified previously for library preparation, sequencing, and variant calling. We asked each panel provider to recruit at least three independent laboratories with which they were remarkably familiar to processing their panels. Each lab made four panel libraries for each reference sample following the harmonized experimental protocols developed by the panel providers accordingly. The libraries from the same panel were sequenced on various sequencing platforms, including Illumina HiSeq 2500, NovaSeq, NextSeq and ThermoFisher IonTorrent S5. Each panel provider performed the downstream analysis using their recommended or in-house bioinformatics pipelines for variant calling, but blinded to any of the known content. Variant calling results were submitted in VCF format, along with their reporting regions and allowlist/blocklist, to an independent group for performance evaluation. In addition to the primary goal of assessing the performance and the clinical applicability of oncopanels, the dataset also holds its value for: i) developing specific bioinformatics pipelines and fine-tuning parameters for more accurate variant calling for a specific panel, ii) developing robust bioinformatics pipelines that can be applied on multiple panels with promising results, iii) investigating ideal sequencing depth for variant calling of a given minimum VAF and certain variant type (i.e., SNVs, small indels and MNVs, or long indels), and iv) making the best use of UMI technology.

## Methods

### Study design

**Fig. 1:**
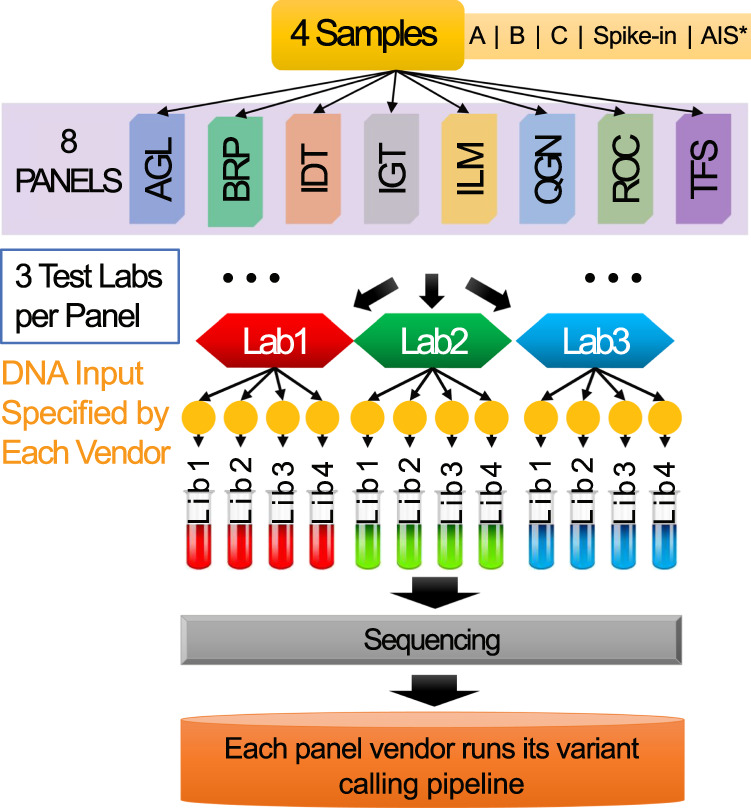
Study design. The illustration demonstrates the workflow of sample distribution, library preparation and sequencing data processing. Four reference samples were created and distributed to each of the testing laboratories. Four technical library replicates were prepared for each sample at each laboratory. DNA libraries were then sequenced and processed by panel-specific bioinformatics pipelines for variant calling. *Sample AIS was not used in the related work. It was designed for a side study which aims to assess the usability of Accugenomics spike-in control as an orthogonal quality control in oncopanel sequencing.

The SEQC2 Oncopanel Sequencing Working Group developed four reference samples for this study, namely: Sample A, Sample B, Sample C and Sample Spike-in (a.k.a., AC5) (Fig. 1). Sample A is the equal mass mixture of DNA from ten cancer cell-lines that are used to make the Agilent UHRR material^3^. Sample B is a cell line derived from a normal male individual available at Agilent. Sample C is the 1:1 dilution of Sample A and Sample B. Sample Spike-in is a mixture of Sample B with 5% AcroMetrix hotspot synthetic controls^4^. There were eight oncopanels included in this study: Agilent Custom Comprehensive Cancer Panel v2 (AGL), Burning Rock DX OncoScreen Plus (BRP), Integrated DNA Technologies xGen Pan-Cancer Panel (IDT), iGeneTech AIOnco-seq (IGT), Illumina TruSight Tumor 170 (ILM), QIAGEN Comprehensive cancer panel (QGN), Roche SeqCap EZ Choice custom PHC Panel (ROC), and Thermo Fisher Oncomine Comprehensive Assay v3 (TFS) (Fig. 1). Each panel provider recruited at least three independent laboratories. We distributed the four reference samples to the test laboratories and each laboratory made four DNA libraries as technical replicates. The libraries from the same panel were sequenced on the same sequencing platforms, including Illumina HiSeq 2500, NovaSeq, NextSeq and ThermoFisher IonTorrent S5. Each panel provider performed the downstream analysis using their recommended or in-house bioinformatics pipelines for variant calling. Variant calling results were submitted in VCF format, along with their reporting regions and allowlist/blocklist, to an independent group, led by Dr. Joshua Xu, for performance evaluation^1^.

### Genomic DNA libraries construction of reference samples

Sample A is composed of an equal mass pooling of 10 gDNA samples prepared from Agilent’s Universal Human Reference RNA (UHRR) cancer cell lines^3^. Over 42 K small variants are identified with high confidence in the defined regions of over 22 million bases^2^.

Sample B is a gDNA sample from a normal male cell line (Agilent Human Reference DNA, Male, Agilent part #: 5190–8848).

Sample C is a 1:1 mix of Sample A and Sample B. Sample C was included to dramatically increase (4x) the number of known variants with VAF between 1% and 2.5%^1^.

Sample Spike-in (a.k.a., AC5) is Sample B with 5% AcroMetrix Spike hotspot synthetic controls (Thermo Fisher Scientific, Fremont, CA)^4^.

Samples A, B, and C were prepared by Agilent and were kindly provided to SEQC2 for study. Sample Spike-in was prepared at Thermo Fisher Scientific (Fremont, CA) after receiving Sample B from Agilent under their material transfer agreement. All four samples were stored in low-EDTA TE buffer (10 mM Tris, 0.1 mM EDTA, pH 8.0) at 20 ng/ul concentration.

### Participating panels sign-up, test sites recruitment and sample distribution

Eight oncopanel providers signed up to participate in this study, each provider recruited at least three independent laboratories which are remarkably familiar with their panels. A total of 28 testing laboratories were initially recruited. One laboratory was later excluded due to affiliation with the panel provider, one laboratory was excluded due to failing to pass quality control, and one laboratory was excluded due to an extended delay in experimental execution and data generation. We distributed the four reference samples to each laboratory. Four DNA libraries for each reference sample were then made at each laboratory as technical replicates. A total of 430 DNA libraries were prepared. Detailed information of the eight participating oncopanels are listed in Table 1. To shorten the description and file names, panel codes were used to identify panels. Laboratory codes are listed in Table 1 to identify the test laboratories for each oncopanels.

**Table 1:** Detailed information for eight participating oncopanels.

Panel code	Panel name*	List of test labs	Size (Kbp)	Gene count	Genome version	DNA input (ng)
AGL	Agilent ClearSeq Comprehensive Cancer Panel v2	ST01/ST02/ST03	7,625	1058	hg19	30
BRP	Burning Rock DX OncoScreen Plus	ST25^†^/ST26/ST27/ST28	1,631	523	hg19	100
IDT	Integrated DNA Technologies xGen Pan-Cancer Panel	ST04/ST05/ST06	780	127	hg19	100
IGT	iGeneTech AIOnco-seq	ST07/ST08/ST09	944	113	hg19	100
ILM	Illumina TruSight Tumor 170	ST10/ST11^‡^/ST12/ST23/ST29	527	154	hg19	50
QGN	QIAGEN Human Comprehensive cancer panel	ST13/ST14/ST15	837	275	hg19	40
ROC	Roche NimbleGen SeqCap EZ Choice PHC Panel	ST16/ST17^§^/ST18/ST19	149	45	hg38	100
TFS	Thermo Fisher Oncomine Comprehensive Assay v3	ST22/ST23/ST24	349	146	hg19	20

Panel code	UMI	Fragmentation approach	Median fragment size (bp)
AGL	Yes (for deduplication)	Covaris E220 instrument/Focused-Ultrasonicators	350
BRP	No	Covaris M220/Focused-Ultrasonicators with AFA Technology	265
IDT	No	Covaris/Focused-Ultrasonicators	300
IGT	No	Covaris/Focused-Ultrasonicators	200 (150–250)
ILM	No	Covaris/Focused-Ultrasonicators	170 (90–250)
QGN	Yes (for error reduction)	QIAGEN Fx enzymatic fragmentation module	183
ROC	Yes (for deduplication)	Kapa Plus enzyme	187
TFS	No	No fragmentation as PCR based target amplification	156

Panel code	Enrichment	Sequencing platform	Read length	Avg. read count	Detection limit (VAF)
AGL	capture based/Agilent SureSelectXT HS Target Enrichment System	HiSeq 2500	2 × 150 bp	362,985,832	1%
BRP	capture based	NovaSeq	2 × 150 bp	89,735,363	1%
IDT	capture based	NovaSeq	2 × 150 bp	124,947,599	2%
IGT	capture based	HiSeq 2500	2 × 125 bp	101,613,666	1%
ILM	capture based	NextSeq	2 × 101 bp	>100 million	2.6%
QGN	one end randomly fragmented and adapter ligated, other end gene specific primer, single primer extension and universal PCR	NovaSeq	2 × 150 bp	141,196,120	0.5%
ROC	capture based	NovaSeq	2 × 150 bp	422,956,489	2.5%
TFS	amplicon based	IonTorrent S5	113 bp (average)	10,765,203	2.5%

This table is expanded version of Supplementary Table 1 in our related work^1^.

^∗^All participating panels are for research use only.

^†^ST25 was excluded from performance analysis as it is a clinical lab closely affiliated with the panel provider.

^‡^ST11 was excluded from performance analysis due to an extended delay in experimental execution and data generation.

^§^ST17 was excluded from performance analysis due to over fragmentation of DNA samples during library preparation.

### Experimental protocols

Basic information about the experimental procedures and wet-lab QC metrics are summarized in Table 1. These experimental protocols are expanded versions of descriptions in our related work^1^, which was approved by the panel providers accordingly.

#### AGL experimental protocol

Genomic DNA libraries were constructed for the reference samples according to the Agilent SureSelectXT HS Target Enrichment System for Illumina Paired-End Multiplexed Sequencing Library Protocol (Cat. No. G9702–90000 Version A1, July 2017). In brief, 30 ng of each sample’s high molecular weight genomic DNA was sonicated in a 50 μl total volume with a Covaris E220 instrument to a mean size of 350 bp (Duty Factor: 10%, Peak Incident Power: 175, Cycles per Burst: 200, Treatment Time: 2 × 30 seconds, Bath Temperature: 2° to 8 °C). DNA fragments were then end-repaired and A-tailed using a two-step cycling protocol (20 °C for 15 minutes and 72 °C for 15 minutes), followed by ligation to XTHS adaptors with UMIs for 30 minutes at 20 °C. Adapter-ligated fragments were amplified and indexed by PCR in a 50 μl total volume with Herculase II Fusion DNA Polymerase under the following conditions: 2 min at 98 °C (initial denaturation), 10 cycle amplification of 30 seconds at 98 °C, 30 seconds at 60 °C, 1 minute at 72 °C, and 5 minutes at 72 °C (final extension). Library quality control (quantity and size distribution) was then assessed using either the 2100 Bioanalyzer and DNA 1000 assay or the 2200 TapeStation and D1000 screen tape. 1 μg of prepared gDNA libraries were then hybridized to a custom Immuno-Oncology focused Comprehensive Cancer Panel (1058 targets coding regions including UTRs and 7.6 Mb in size) biotinylated RNA probes (5 minutes at 95 °C, 10 minutes at 65 °C, 1 minute at 65 °C, 60 cycles of 1 minute at 65 °C and 3 seconds at 37 °C, and 65 °C hold) and captured with Dynabeads MyOne Streptavidin T1 beads. SureSelect enriched gDNA libraries were PCR amplified using on-bead protocol in a 50 μl total volume with Herculase II Fusion DNA Polymerase under the following conditions: 2 min at 98 °C (initial denaturation), 10 cycles of 30 seconds at 98 °C, 30 seconds at 60 °C, 1 minute at 72 °C (amplification), and 5 minutes at 72 °C (final extension), followed by 4 °C hold. All DNA purifications between steps were performed with AMPure XP beads as indicated in the user manual. Post-capture library quality control was assessed using either the 2100 Bioanalyzer and a High Sensitivity DNA assay or the 2200 TapeStation and HSD1000 screen tape. Indexed samples were finally pooled and sequenced to approximately 5000X (Samples A, B, AC5, AAG) or 10,000X (Sample C) read depth on a NovaSeq 6000 instrument using a 2x150bp Paired-End protocol (Q30 scores ≥ 75%).

#### BRP experimental protocol

The library prep and enrichment process were performed using Burning Rock HS library preparation kit without modification. In brief, DNA shearing was performed on SEQC2 gDNA reference samples using Covaris M220 for 195 S, with Peak Incident Power = 50 W, Duty Factor: 20%, Cycle Per Burst: 200, at 6–8 °C, followed by end repair, adaptor ligation and PCR enrichment. About 1 μg of purified pre-enrichment library was hybridized to the OncoScreenPlus^TM^ panel and further enriched following manufacturer instruction. The OncoScreenPlus^TM^ panel is about 1.7 M bp in size and covers 520 human cancer related genes. Final DNA libraries were quantified using Qubit Fluorometer with dsDNA HS assay kit (Life Technologies, Carlsbad, CA). A LabChip GX Touch System, Agilent 2100 bioanalyzer or Agilent 4200 TapeStation D1000 ScreenTape was then performed to assess the quality and size distribution of the library. The libraries were sequenced on the NovaSeq 6000 sequencer (Illumina, Inc., California, US) with 2 × 150 bp paired-end reads with a unique dual index.

#### IDT experimental protocol

Libraries were constructed using 100 ng from each of the reference samples in quadruplicate. Briefly, genomic DNA was sheared to 300 bp (Covaris) followed by library construction using the NEBNext Ultra II DNA Library Prep Kit for Illumina and xGen Dual Index UMI Adapters–Tech Access. End repair and A-tailing were performed according to the manufacturer’s recommendations. Adapters were ligated using 5 µL of 15 µM stock for each reaction followed by 0.9X AMPure purification. Libraries were amplified with Illumina P5 and P7 primers using NEBNext Ultra II Q5 Master Mix using 6 cycles of amplification. Libraries were purified using 1X AMPure clean up and quantified by Qubit. Following library construction, 500 ng of each library was captured with the xGen Pan-Cancer Panel v1.5 following the manufacturer’s instructions using xGen Universal Blockers–TS Mix. Hydrophilic Streptavidin Magnetic Beads and NEBNext Ultra II Q5 Master Mix were substituted for the capture and post-capture amplification using 16 cycles of PCR. Libraries were purified, quantified, and pooled for sequencing on an Illumina NovaSeq S4 flow cell.

#### IGT experimental protocol

The iGeneTech AIOnco-seq gene panel was designed to target the exons of 113 cancer related genes. The total size of targeted regions was 944,153 bp. 100 ng of human genomic DNA was sheared on a Covaris S220 focused-ultrasonicator for 180 s (175 W peak incident power, 10% duty factor, and 200 cycles per burst). The resulting fragment-size distribution was about 150~250 bp. End repair, nontemplated dA-tailing, and adapter ligation were performed by using KAPA Hyper Prep Kits. The adapter was generated by annealing oligonucleotide ACACTCTTTCCCTACACGACGCTCTTCCGATC*T (the * represent a phosphorothioate bond) and oligonucleotide Pho-GATCGGAAGAGCACACGTCTGAACTCCAGTCAC. The ligation product was cleaned up and size-selected by using Beckman Ampure XP Beads (Beckman). The purified ligated product was amplified with 7 cycles of PCR to generate about 1 μg library.

A mix containing 750 ng whole genome library, 2.5 μg human Cot-1 DNA (Invitrogen), 2.5 μg salmon sperm DNA, and 2000 pmol adapter blocking oligonucleotides was concentrated to 9 μl, and then heated at 95 °C for 5 min, and held at 65 °C for 5 min. A 13 μl of 65 °C prewarmed hybridization buffer (iGeneTech) was added to the library. A 7 μl of freshly prepared, 65 °C prewarmed mixture containing 200 ng biotinylated RNA probes and 20 U SUPERase-In (Invitrogen) was added to the library and mixed by a pipette. After 16 h at 65 °C, the hybridization mix was added to 50 ul Dynabeads MyOne Streptavidin T1 (Invitrogen), which had been washed three times and resuspended in 200 μl binding buffer (iGeneTech). The binding reaction was rotated on a rotational mixer (10 rpm/min) at 25 °C for 30 min, washed once at 25 °C for 15 min with 200 μl Wash Buffer I (iGeneTech), and wash three times at 65 °C for 15 min with Wash Buffer II (iGeneTech). The beads were resuspended in 20 μl TE Buffer. The post-hybridization PCR was performed to enrich and amplify the target region library. The libraries were sequenced on an Illumina HiSeq 2500 sequencer with 125 bp paired-end sequencing reads.

#### ILM experimental protocol

DNA was processed according to the TruSight Tumor 170 Reference Guide^5^, briefly samples were sheared using Covaris to approximately 90–250 bp. Following shearing, DNA fragments were end-repaired and A-tailed in a single reaction, followed by ligation to a universal adapter. Post-ligation clean-up was performed using SPRI beads and then libraries were indexed using unique dual indexes by PCR. Target regions were captured using an overnight hybridization to biotinylated target specific oligos which covered 533Kb of genomic targets across 154 genes, followed by capture with streptavidin magnetic beads. A second hybridization and capture reaction were performed followed by PCR amplification using the universal primers compatible with Illumina’s sequencing flowcells. Libraries were normalized using bead-based normalization before being pooled in equal parts and sequenced, 10 samples per flowcell, on NextSeq v2 high-output flowcell. Sequencing reads were 2 × 101 bp with 8 bp dual indexed reads. Five independent labs were recruited as the test labs for ILM panel. One test lab was excluded from performance analysis due to an extensive delay in data generation and some deviation from the experimental protocol.

#### QGN experimental protocol

The Human Comprehensive Cancer QIASeq DNA Panel is a targeted enrichment panel that enriches genes using single primer extension and constructs libraries using integrated unique molecular indices (UMIs). The panel uses 11,311 primers in a single tube mix to enrich over 0.83 Mb region across full coding areas of 275 cancer genes. The selected genes harbor mutations that are commonly involved in cancer development and progression. Genomic DNA samples were first fragmented, end repaired and A-tailed within a single, controlled multi-enzyme reaction. The prepared DNA fragments were then ligated at their 5′ ends with an Illumina sequencing platform-specific adapter containing a 12-base fully random sequence of UMI and sample index. Therefore, each original DNA molecule in the sample received a unique UMI sequence during ligation prior to target enrichment and library amplification PCR. This allowed the correction of errors associated with PCR amplification and sequencing.

Target enrichment with Human Comprehensive Cancer QIASeq DNA Panel was performed post-UMI assignment to ensure that targeting regions of DNA molecules containing UMIs were sufficiently and uniformly enriched in the sequenced library. For enrichment, ligated DNA molecules were subject to six cycles of targeted PCR using one region-specific primer and one universal primer complementary to the adapter. After enrichment PCR, nineteen cycles of Universal PCR were carried out to further amplify the target enriched library and added platform specific adapter sequences and additional sample indices. Finished libraries were checked on Bioanalyzer for proper size distribution and library concentration was measured by QIAseq Library Quantification system. Libraries were normalized to 5 nM and pooled together according to quantified library concentration. Pooled libraries were sent out to Illumina for sequencing on NovaSeq with a Qiagen custom read 1 sequencing primer. Libraries within a test site were loaded on each lane of the flowcell for sequencing.

#### ROC experimental protocol

The panel design utilized in the study is 170907_HG38_SEQC2_PHC_EZ. This is a research panel, not intended for commercial development. The panel consists of coding sequence (CDS) from 33 cancer genes from SEQC Pan Cancer Gene list and CDS regions overlapping the Accugenomics controls. Additional targets were derived from the Roche Avenio ctDNA Panels. The panel consists of 45 genes in total (~131 kb of targets). The panel also targets 730 putative structural variant breakpoints derived from the 10X cell line sequencing (~206 kb of targets).

The ROC panel utilized an enzymatic fragmentation library prep followed by a hybridization-based workflow. The extracted DNA sample (100 ng) was fragmented using the KAPA HyperPlus Library Preparation Kit, and then ligated to Illumina-compatible, unique-dual-index (UDI) sequencing library adapters (IDT). After the ligation, the DNA library was amplified using the KAPA HiFi HotStart Ready Mix. Amplified libraries were quantified with a fluorometric method, and the maximum available amount of library (> = 0.5 ug and < = 2.5 ug) was utilized for capture. The sample was incubated overnight at 47 C with the gene panel (170907_HG38_SEQC2_PHC_EZ), which consisted of biotinylated DNA probes designed to capture the genes and regions of interest. Universal Blocking Oligos were utilized in the hybridization to prevent cross-hybridization between sequencing adapters and to increase capture specificity. The desired DNA-probe complexes were then captured on streptavidin beads, and after a series of washes, first at 47 C and then at room temperature, the samples were amplified using ligation mediated PCR (LM-PCR). The final product of the workflow was enriched libraries ready for sequencing. The final sequencing libraries were sequenced (2 × 150 bp) using the Illumina NovaSeq sequencing platform.

Four independent labs were recruited to test the ROC panel. Initial sequencing data QC analysis led to the exclusion of one test lab from performance analysis. Shorter fragments, which resulted from over fragmentation during library preparation, indicated a likely deviation from the experimental protocol.

#### TFS experimental protocol

The Oncomine Comprehensive Assay DNA v3C – Chef Ready Kit^6^ was used to generate libraries for next-generation sequencing on the Ion Torrent S5 platform. This assay enables analysis of variants across 146 genes and covers 350,350 bp of DNA target sequence. It typically is used in combination with an accompanying RNA assay bringing the gene total to 161, but the study used only the DNA assay with the RNA component omitted. The gene content of the Oncomine Comprehensive Assay was prioritized to detect relevant somatic variants in solid tumors with associations to published evidence including the indication statements of approved cancer drugs, consensus clinical treatment guidelines, and the enrolment criteria of oncology clinical trials. Purified DNA was extracted using the RecoverAll Multi-Sample RNA/DNA Isolation Workflow^7^. Four DNA samples were used to prepare duplicate libraries on each of two runs using the Ion Chef Instrument. An input of 10 ng per primer pool of DNA was used, and libraries were generated following the manufacturer’s instructions in the Oncomine Comprehensive Assay User Guide^8^ and the Ion AmpliSeq Library Preparation on the Ion Chef User Guide^9^. A total of 8 DNA sample libraries were combined for template preparation on the Ion Chef Instrument using the Ion 540 Kit-Chef^10^. Sequencing was performed with the Ion S5 XL System^11^ and the Ion 540 Chip^12^.

### Sequencing, Data processing and collection

The libraries from the same panel were sequenced on the same sequencing platforms chosen by the panel providers. Even though four sequencing platforms were used in this study, including three Illumina platforms (HiSeq 2500, NovaSeq, NextSeq) and one ThermoFisher platform (IonTorrent S5), each library was sequenced on only one of the platforms. Comparison across sequencing platforms of the exact same DNA library is not available in this study. Sequencing data was required to be shared within the SEQC2 Oncopanel Working Group via Illumina BaseSpace Sequence Hub or by uploading data to the sFTP server hosted at Stanford University. Either FASTQ or BAM format was used for data sharing. All the data was collected at the National Center for Toxicological Research (NCTR), organized and renamed in a consistent manner, and then submitted to NCBI SRA data repository. The data became publicly available upon the publication of the related research manuscript^1^.

### Bioinformatics pipelines and variant calling results

Each panel provider ran their recommended or in-house pipelines for variant calling and filtering. In this study, we considered each recommended or in-house pipeline to be part of the solution of variant detection along with the according oncopanel. The purpose of our study was not to determine the superior method for variant calling; thus, the recommended or in-house pipelines were used as they should perform reasonably at variant calling. The reproducibility, sensitivity, and false positive rate of the eight participating oncopanels were reported in the related research manuscript^1^. If the inquisitive reader is interested in comparing the variant calling results between the recommended or in-house pipelines, the VCF files can be downloaded at figshare^13^.

## Data Records

The data have been deposited to NCBI SRA with accession number SRP295113^14^. BioSample metadata are available in the NCBI BioSample database (http://www.ncbi.nlm.nih.gov/biosample/), where Sample A is under accession number SAMN16786360^15^ with download size of 879 Gb, Sample B is under accession number SAMN16786361^16^ with download size of 801 Gb, Sample C is under accession number SAMN16786362^17^ with download size of 1.37 Tb, and Sample Spike-in is under accession number SAMN16786363^18^ with download size of 2.47 Tb. There are 400 NCBI SRA records in total for this study, 103 for Sample A, 103 for Sample B, 91 for Sample C, and 103 for Sample Spike-in. Some of the data for Panel ILM was not available due to an unrecoverable data loss, thus, these data files are not submittable to NCBI SRA. A detailed list of all 400 NCBI SRA records can be found in Online-only Table 1. An extra Sample AIS^19^, which contains 103 SRA records, can also be found under the BioProject PRJNA677997 umbrella. This sample was not used in the related publication^1^, but was designed for a side study to assess the usability of Accugenomics spike-in control as an orthogonal quality control in oncopanel sequencing. If interested in analyzing the Sample AIS, a brief description about the sample can be found in the Usage Notes. The “LibraryName” column shows the individual DNA library identifier, which combines the sample ID, panel code, testing laboratory code, DNA input amount, and library replicate together with “_”. There is a number “1” immediately after the panel code, which indicates the oncopanel study shown here. The “FileType” column show the file type of the according NCBI SRA record, where “fastq” indicates the FASTQ format and “bam” indicated the BAM format. Data for panel ROC is provided in unmapped BAM format. Data for TFS is provided in mapped BAM format, and the reference genome used for read mapping is listed in the “ReferenceGenome” column. Data for the other six panels is provided in FASTQ format. Details of read processing for all panels and read mapping for panel TFS can be found in Code Availability. Several filenames may be listed in columns “filename1” to “filename8”. These filenames are the original filenames used when uploading the data files to NCBI SRA. If retrieving the data using NCBI’s SRA ToolKit, one may see different filenames, which may be related to the SRR accession number. Filenames start with “LibraryName”, followed by part number if applicable, then the read identifier and filename extension. If the file type is “bam”, there is one file listed for each record, ending with “.bam” as the filename extension. If the file type is “fastq”, the files end with “.fastq.gz”. You will find pairs of files listed for each record, where “R1” and “R2” refer to the left and right reads. Specifically, for panel AGL, “R1” indicates the left reads, “R2” indicates the UMI barcodes, and “R3” indicates the right reads. To make the file size manageable for data transfer, we split bigger files into parts. For some records, there may be “PT1”, “PT2”, “PT3”, “PT4” in the filenames, which indicates different parts of the same data files. These parts of the same data files need to be merged in the order of 1 to 4 (or 2 if there are only two parts) before any data processing.

Data from three testing laboratories (ST25, ST11, ST17) were excluded from the study due to the clinical lab being too closely affiliated with the panel provider (ST25), an extended delay in experimental execution and data generation (ST11), and over fragmentation of DNA samples during library preparation (ST17). Due to an unexpected hard drive failure, 20 files for panel ILM were unable to be recovered, including data for ST12 (LIB3 and LIB4), ST29 (LIB1, LIB2 and LIB4).

## Technical Validation

The dataset is validated in multiple aspects, (a) each of the four reference samples was prepared and sequenced four times at one testing laboratory, and is also prepared and sequenced at least 12 times (three or more testing laboratories per oncopanel) for each oncopanel; high intra- and cross-lab reproducibility was observed;^1^ (b) Sample Spike-in has known variants and shows very high sensitivity with a handful of readily explainable exceptions;^1^ (c) few false positive calls were reported with Sample B for all panels^1^, which indicates a low error rate of the sequencing data and high quality calling.

## Usage Notes

As all eight oncopanels were designed to target certain genomic regions, variant calling should be restricted to the designed regions of each oncopanel. The corresponding regions were provided in BED format and can be downloaded from figshare^20^. Due to the choice of reference genome in the oncopanel design, for all panels except ROC, the regions were provided for GRCh37/hg19; and for the ROC panel, the regions were provided for GRCh38/hg38. A blocklist of AcroMetrix Oncology Hotspot Control (AOHC) variants for panel TFS is also provided, which should be used when analyzing Sample AC5 with panel TFS.

The extra Sample AIS was composed of Sample A and enzymatically fragmented Accugenomics spike-in control. As stated in our related work, the raw reads from Sample AIS should map against both the native template (NT) of reference genome (hg19 or hg38) and the modified internal standard (IS) genome template to separate the NT and IS reads^21^.

The 500+ enzymatically fragmented Acrometrix Synthetic Hotspot controls^4^ were added to Sample B to make Sample AC5. These variants can be used as positive controls for analyzing Sample AC5. The list of Acrometrix Synthetic Hotspot controls is provided in VCF format for both hg19 and hg38 and can be downloaded from figshare^22^.

This manuscript reflects the views of the authors and does not necessarily reflect those of the U.S. Food and Drug Administration. Any mention of commercial products is for clarification only and is not intended as approval, endorsement, or recommendation.

## Data Availability

The details and computational parameters of raw read processing for all panels and read mapping for panel TFS are expanded versions of descriptions in our related work^1^. **AGL read processing** Each sample was demultiplexed using bcl2fastq v2.20 (Illumina)^23^ with the base mask Y150, I8, Y10, Y150 and all default settings except for mask-short-adapter-reads, which was set to 0. Adapters were trimmed using AGeNT Trimmer (Agilent)^24^. **BRP read processing** After demultiplexing using bcl2fastq v2.20^23^, sequence data were filtered using the Trimmomatic 0.36^25^ with parameters “TRAILING:20 SLIDINGWINDOW:30:25 MINLEN:50”. **IDT read processing** IDT libraries were prepared with xGen Dual Index UMI Adapters—Tech Access which contain 9 bp degenerate unique molecular identifiers (UMIs) downstream of the i7 sample index. To demultiplex Illumina sequencing data containing UMIs, Illumina basecall (BCL) files were used to generate demultiplexed BAM files using Picard v2.9.0 (http://broadinstitute.github.io/picard/) IlluminaBasecallsToSam. Prior to running Picard v2.9.0 IlluminaBasecallsToSam, Picard v2.9.0 ExtractIlluminaBarcodes was used to determine the barcode for each read using the read structure 151T8B9S8B151T (T = template, B = sample barcode, M = molecular barcode, and S = skip). The UMI bases were not used for downstream analysis since there was not enough raw sequencing depth for consensus analysis. After demultiplexing, FASTQ files were generated using Picard v2.9.0 SamToFastq. FASTQ files were downsampled to an equivalent read count per sample using seqtk v1.0 (https://github.com/lh3/seqtk). **IGT read processing** Raw reads were firstly quality trimmed with Trimmomatic 0.36^25^, using an 8-base-pair sliding-window algorithm with a quality score cutoff of 20, clipping off ends with at least one occurrence of a quality score below 20, and discarding reads that dropped below a length of 40 base-pairs. Adaptors (a1: GATCGGAAGAGCACACGTCT, a2: AGATCGGAAGAGCGTCGTGTAGGGAAAGAGTGT) were removed simultaneously. **ILM read processing** Pan Cancer sample testing analysis was performed using the standard TruSight Tumor 170 pipeline available on the BaseSpace Sequence Hub. Briefly, high level sequencing run metrics are evaluated to generate a Run QC Metrics report. Next, reads are converted into the FASTQ format using bcl2fastq v2.20^23^ and adapters are trimmed. **QGN read processing** Raw sequencing data were demultiplexed and converted into the FASTQ format using bcl2fastq v2.20^23^ in BaseSpace, with the library specific pairs of Qiagen custom read 1 primer sequences and the reverse complementary Illumina i7 primer sequences. QIAGEN’s UMI-aware variant caller smCounter2^26^ can be used for variant calling. **ROC read processing** Two NovaSeq runs were provided by Illumina via BaseSpace (RUN1 = SeqC2_ROC1_ST_16_17_18_19; RUN2 = SeqC2_ROC1_ST16_17_18_19). BCL files were converted to unaligned BAM files using instructions provided by IDT (https://www.idtdna.com/pages/products/next-generation-sequencing/adapters/xgen-dual-index-umi-adapters-tech-access). Following the IDT tech note, Picard 2.18.3 ExtractIlluminaBarcodes and IlluminaBasecallsToSam were used to create the unaligned BAM files. The UMI for each fragment is stored in the RX tag in the BAM file. **TFS read processing and mapping** Signal processing and base calling were performed using Torrent Suite Software 5.8 (https://github.com/iontorrent/TS) using default parameters for the Oncomine Comprehensive Assay. The signal processing step consists of modeling the pH dynamics on the semiconductor surface taking into account the varying local pH in each individual sensor coming from the different reagent flows across the chip and from any nucleotide incorporation that may be happening over each sensor^27^. The base calling step consists of taking the estimated levels of nucleotide incorporation for each read and each nucleotide flow, and modeling the de-phasing process whereby some templates within each clonally-amplified population run ahead or behind in terms of their nucleotide incorporation. During the base calling process, sample-specific barcodes and 3′ adapters are annotated. After completion of primary analysis with Torrent Suite Software 5.8, reads were uploaded to Ion Reporter Software 5.6^28^ for subsequent processing. Reads were aligned with the Torrent Mapping Alignment Program (TMAP, https://github.com/iontorrent/TS/tree/master/Analysis/TMAP), which uses the BWA fastmap routine to map reads and applies post-processing of the alignments to optimize for technology-specific error patterns.

## Online-only Table

**Online-only Table 1:** Metadata for the 400 NCBI SRA records.

Sample	BioSample	PanelCode	Experiment	Run	Instrument	LibraryName	LibraryLayout	FileType	ReferenceGenome	Bytes	filename1	filename2	filename3	filename4	filename5	filename6	filename7	filename8
Sample A	SAMN16786360	AGL1	SRX9623365	SRR13189055	Illumina NovaSeq 6000	SampleA_AGL1_ST01_30ng_LIB1	PAIRED	fastq		8.90 GB	SampleA_AGL1_ST01_30ng_LIB1.R1.fastq.gz	SampleA_AGL1_ST01_30ng_LIB1.R2.fastq.gz	SampleA_AGL1_ST01_30ng_LIB1.R3.fastq.gz					
Sample A	SAMN16786360	AGL1	SRX9623366	SRR13189054	Illumina NovaSeq 6000	SampleA_AGL1_ST01_30ng_LIB2	PAIRED	fastq		9.35 GB	SampleA_AGL1_ST01_30ng_LIB2.R1.fastq.gz	SampleA_AGL1_ST01_30ng_LIB2.R2.fastq.gz	SampleA_AGL1_ST01_30ng_LIB2.R3.fastq.gz					
Sample A	SAMN16786360	AGL1	SRX9623377	SRR13189043	Illumina NovaSeq 6000	SampleA_AGL1_ST01_30ng_LIB3	PAIRED	fastq		7.91 GB	SampleA_AGL1_ST01_30ng_LIB3.R1.fastq.gz	SampleA_AGL1_ST01_30ng_LIB3.R2.fastq.gz	SampleA_AGL1_ST01_30ng_LIB3.R3.fastq.gz					
Sample A	SAMN16786360	AGL1	SRX9623388	SRR13189032	Illumina NovaSeq 6000	SampleA_AGL1_ST01_30ng_LIB4	PAIRED	fastq		8.80 GB	SampleA_AGL1_ST01_30ng_LIB4.R1.fastq.gz	SampleA_AGL1_ST01_30ng_LIB4.R2.fastq.gz	SampleA_AGL1_ST01_30ng_LIB4.R3.fastq.gz					
Sample A	SAMN16786360	AGL1	SRX9623399	SRR13189021	Illumina NovaSeq 6000	SampleA_AGL1_ST02_30ng_LIB1	PAIRED	fastq		4.83 GB	SampleA_AGL1_ST02_30ng_LIB1.R1.fastq.gz	SampleA_AGL1_ST02_30ng_LIB1.R2.fastq.gz	SampleA_AGL1_ST02_30ng_LIB1.R3.fastq.gz					
Sample A	SAMN16786360	AGL1	SRX9623410	SRR13189010	Illumina NovaSeq 6000	SampleA_AGL1_ST02_30ng_LIB2	PAIRED	fastq		5.35 GB	SampleA_AGL1_ST02_30ng_LIB2.R1.fastq.gz	SampleA_AGL1_ST02_30ng_LIB2.R2.fastq.gz	SampleA_AGL1_ST02_30ng_LIB2.R3.fastq.gz					
Sample A	SAMN16786360	AGL1	SRX9623421	SRR13188999	Illumina NovaSeq 6000	SampleA_AGL1_ST02_30ng_LIB3	PAIRED	fastq		1.41 GB	SampleA_AGL1_ST02_30ng_LIB3.R1.fastq.gz	SampleA_AGL1_ST02_30ng_LIB3.R2.fastq.gz	SampleA_AGL1_ST02_30ng_LIB3.R3.fastq.gz					
Sample A	SAMN16786360	AGL1	SRX9623422	SRR13188998	Illumina NovaSeq 6000	SampleA_AGL1_ST02_30ng_LIB4	PAIRED	fastq		5.93 GB	SampleA_AGL1_ST02_30ng_LIB4.R1.fastq.gz	SampleA_AGL1_ST02_30ng_LIB4.R2.fastq.gz	SampleA_AGL1_ST02_30ng_LIB4.R3.fastq.gz					
Sample A	SAMN16786360	AGL1	SRX14332179	SRR18185264	Illumina NovaSeq 6000	SampleA_AGL1_ST03_30ng_LIB1	PAIRED	fastq		7.21 GB	SampleA_AGL1_ST03_30ng_LIB1_PT1.R1.fastq.gz	SampleA_AGL1_ST03_30ng_LIB1_PT1.R2.fastq.gz	SampleA_AGL1_ST03_30ng_LIB1_PT1.R3.fastq.gz	SampleA_AGL1_ST03_30ng_LIB1_PT2.R1.fastq.gz	SampleA_AGL1_ST03_30ng_LIB1_PT2.R2.fastq.gz	SampleA_AGL1_ST03_30ng_LIB1_PT2.R3.fastq.gz		
Sample A	SAMN16786360	AGL1	SRX14332180	SRR18185263	Illumina NovaSeq 6000	SampleA_AGL1_ST03_30ng_LIB2	PAIRED	fastq		9.51 GB	SampleA_AGL1_ST03_30ng_LIB2_PT1.R1.fastq.gz	SampleA_AGL1_ST03_30ng_LIB2_PT1.R2.fastq.gz	SampleA_AGL1_ST03_30ng_LIB2_PT1.R3.fastq.gz	SampleA_AGL1_ST03_30ng_LIB2_PT2.R1.fastq.gz	SampleA_AGL1_ST03_30ng_LIB2_PT2.R2.fastq.gz	SampleA_AGL1_ST03_30ng_LIB2_PT2.R3.fastq.gz		
Sample A	SAMN16786360	AGL1	SRX14332191	SRR18185252	Illumina NovaSeq 6000	SampleA_AGL1_ST03_30ng_LIB3	PAIRED	fastq		7.98 GB	SampleA_AGL1_ST03_30ng_LIB3_PT1.R1.fastq.gz	SampleA_AGL1_ST03_30ng_LIB3_PT1.R2.fastq.gz	SampleA_AGL1_ST03_30ng_LIB3_PT1.R3.fastq.gz	SampleA_AGL1_ST03_30ng_LIB3_PT2.R1.fastq.gz	SampleA_AGL1_ST03_30ng_LIB3_PT2.R2.fastq.gz	SampleA_AGL1_ST03_30ng_LIB3_PT2.R3.fastq.gz		
Sample A	SAMN16786360	AGL1	SRX14332192	SRR18185251	Illumina NovaSeq 6000	SampleA_AGL1_ST03_30ng_LIB4	PAIRED	fastq		7.18 GB	SampleA_AGL1_ST03_30ng_LIB4_PT1.R1.fastq.gz	SampleA_AGL1_ST03_30ng_LIB4_PT1.R2.fastq.gz	SampleA_AGL1_ST03_30ng_LIB4_PT1.R3.fastq.gz	SampleA_AGL1_ST03_30ng_LIB4_PT2.R1.fastq.gz	SampleA_AGL1_ST03_30ng_LIB4_PT2.R2.fastq.gz	SampleA_AGL1_ST03_30ng_LIB4_PT2.R3.fastq.gz		
Sample Spike-in	SAMN16786363	AGL1	SRX9623369	SRR13189051	Illumina NovaSeq 6000	SampleAC5_AGL1_ST01_30ng_LIB1	PAIRED	fastq		9.89 GB	SampleAC5_AGL1_ST01_30ng_LIB1.R1.fastq.gz	SampleAC5_AGL1_ST01_30ng_LIB1.R2.fastq.gz	SampleAC5_AGL1_ST01_30ng_LIB1.R3.fastq.gz					
Sample Spike-in	SAMN16786363	AGL1	SRX9623370	SRR13189050	Illumina NovaSeq 6000	SampleAC5_AGL1_ST01_30ng_LIB2	PAIRED	fastq		9.13 GB	SampleAC5_AGL1_ST01_30ng_LIB2.R1.fastq.gz	SampleAC5_AGL1_ST01_30ng_LIB2.R2.fastq.gz	SampleAC5_AGL1_ST01_30ng_LIB2.R3.fastq.gz					
Sample Spike-in	SAMN16786363	AGL1	SRX9623371	SRR13189049	Illumina NovaSeq 6000	SampleAC5_AGL1_ST01_30ng_LIB3	PAIRED	fastq		8.13 GB	SampleAC5_AGL1_ST01_30ng_LIB3.R1.fastq.gz	SampleAC5_AGL1_ST01_30ng_LIB3.R2.fastq.gz	SampleAC5_AGL1_ST01_30ng_LIB3.R3.fastq.gz					
Sample Spike-in	SAMN16786363	AGL1	SRX9623372	SRR13189048	Illumina NovaSeq 6000	SampleAC5_AGL1_ST01_30ng_LIB4	PAIRED	fastq		9.18 GB	SampleAC5_AGL1_ST01_30ng_LIB4.R1.fastq.gz	SampleAC5_AGL1_ST01_30ng_LIB4.R2.fastq.gz	SampleAC5_AGL1_ST01_30ng_LIB4.R3.fastq.gz					
Sample Spike-in	SAMN16786363	AGL1	SRX9623373	SRR13189047	Illumina NovaSeq 6000	SampleAC5_AGL1_ST02_30ng_LIB1	PAIRED	fastq		5.12 GB	SampleAC5_AGL1_ST02_30ng_LIB1.R1.fastq.gz	SampleAC5_AGL1_ST02_30ng_LIB1.R2.fastq.gz	SampleAC5_AGL1_ST02_30ng_LIB1.R3.fastq.gz					
Sample Spike-in	SAMN16786363	AGL1	SRX9623374	SRR13189046	Illumina NovaSeq 6000	SampleAC5_AGL1_ST02_30ng_LIB2	PAIRED	fastq		5.04 GB	SampleAC5_AGL1_ST02_30ng_LIB2.R1.fastq.gz	SampleAC5_AGL1_ST02_30ng_LIB2.R2.fastq.gz	SampleAC5_AGL1_ST02_30ng_LIB2.R3.fastq.gz					
Sample Spike-in	SAMN16786363	AGL1	SRX9623375	SRR13189045	Illumina NovaSeq 6000	SampleAC5_AGL1_ST02_30ng_LIB3	PAIRED	fastq		6.05 GB	SampleAC5_AGL1_ST02_30ng_LIB3.R1.fastq.gz	SampleAC5_AGL1_ST02_30ng_LIB3.R2.fastq.gz	SampleAC5_AGL1_ST02_30ng_LIB3.R3.fastq.gz					
Sample Spike-in	SAMN16786363	AGL1	SRX9623376	SRR13189044	Illumina NovaSeq 6000	SampleAC5_AGL1_ST02_30ng_LIB4	PAIRED	fastq		6.41 GB	SampleAC5_AGL1_ST02_30ng_LIB4.R1.fastq.gz	SampleAC5_AGL1_ST02_30ng_LIB4.R2.fastq.gz	SampleAC5_AGL1_ST02_30ng_LIB4.R3.fastq.gz					
Sample Spike-in	SAMN16786363	AGL1	SRX14332193	SRR18185250	Illumina NovaSeq 6000	SampleAC5_AGL1_ST03_30ng_LIB1	PAIRED	fastq		7.35 GB	SampleAC5_AGL1_ST03_30ng_LIB1_PT1.R1.fastq.gz	SampleAC5_AGL1_ST03_30ng_LIB1_PT1.R2.fastq.gz	SampleAC5_AGL1_ST03_30ng_LIB1_PT1.R3.fastq.gz	SampleAC5_AGL1_ST03_30ng_LIB1_PT2.R1.fastq.gz	SampleAC5_AGL1_ST03_30ng_LIB1_PT2.R2.fastq.gz	SampleAC5_AGL1_ST03_30ng_LIB1_PT2.R3.fastq.gz		
Sample Spike-in	SAMN16786363	AGL1	SRX14332194	SRR18185249	Illumina NovaSeq 6000	SampleAC5_AGL1_ST03_30ng_LIB2	PAIRED	fastq		10.89 GB	SampleAC5_AGL1_ST03_30ng_LIB2_PT1.R1.fastq.gz	SampleAC5_AGL1_ST03_30ng_LIB2_PT1.R2.fastq.gz	SampleAC5_AGL1_ST03_30ng_LIB2_PT1.R3.fastq.gz	SampleAC5_AGL1_ST03_30ng_LIB2_PT2.R1.fastq.gz	SampleAC5_AGL1_ST03_30ng_LIB2_PT2.R2.fastq.gz	SampleAC5_AGL1_ST03_30ng_LIB2_PT2.R3.fastq.gz		
Sample Spike-in	SAMN16786363	AGL1	SRX14332195	SRR18185248	Illumina NovaSeq 6000	SampleAC5_AGL1_ST03_30ng_LIB3	PAIRED	fastq		8.49 GB	SampleAC5_AGL1_ST03_30ng_LIB3_PT1.R1.fastq.gz	SampleAC5_AGL1_ST03_30ng_LIB3_PT1.R2.fastq.gz	SampleAC5_AGL1_ST03_30ng_LIB3_PT1.R3.fastq.gz	SampleAC5_AGL1_ST03_30ng_LIB3_PT2.R1.fastq.gz	SampleAC5_AGL1_ST03_30ng_LIB3_PT2.R2.fastq.gz	SampleAC5_AGL1_ST03_30ng_LIB3_PT2.R3.fastq.gz		
Sample Spike-in	SAMN16786363	AGL1	SRX14332196	SRR18185247	Illumina NovaSeq 6000	SampleAC5_AGL1_ST03_30ng_LIB4	PAIRED	fastq		7.65 GB	SampleAC5_AGL1_ST03_30ng_LIB4_PT1.R1.fastq.gz	SampleAC5_AGL1_ST03_30ng_LIB4_PT1.R2.fastq.gz	SampleAC5_AGL1_ST03_30ng_LIB4_PT1.R3.fastq.gz	SampleAC5_AGL1_ST03_30ng_LIB4_PT2.R1.fastq.gz	SampleAC5_AGL1_ST03_30ng_LIB4_PT2.R2.fastq.gz	SampleAC5_AGL1_ST03_30ng_LIB4_PT2.R3.fastq.gz		
Sample B	SAMN16786361	AGL1	SRX9623395	SRR13189025	Illumina NovaSeq 6000	SampleB_AGL1_ST01_30ng_LIB1	PAIRED	fastq		9.61 GB	SampleB_AGL1_ST01_30ng_LIB1.R1.fastq.gz	SampleB_AGL1_ST01_30ng_LIB1.R2.fastq.gz	SampleB_AGL1_ST01_30ng_LIB1.R3.fastq.gz					
Sample B	SAMN16786361	AGL1	SRX9623396	SRR13189024	Illumina NovaSeq 6000	SampleB_AGL1_ST01_30ng_LIB2	PAIRED	fastq		8.89 GB	SampleB_AGL1_ST01_30ng_LIB2.R1.fastq.gz	SampleB_AGL1_ST01_30ng_LIB2.R2.fastq.gz	SampleB_AGL1_ST01_30ng_LIB2.R3.fastq.gz					
Sample B	SAMN16786361	AGL1	SRX9623397	SRR13189023	Illumina NovaSeq 6000	SampleB_AGL1_ST01_30ng_LIB3	PAIRED	fastq		9.48 GB	SampleB_AGL1_ST01_30ng_LIB3.R1.fastq.gz	SampleB_AGL1_ST01_30ng_LIB3.R2.fastq.gz	SampleB_AGL1_ST01_30ng_LIB3.R3.fastq.gz					
Sample B	SAMN16786361	AGL1	SRX9623398	SRR13189022	Illumina NovaSeq 6000	SampleB_AGL1_ST01_30ng_LIB4	PAIRED	fastq		7.76 GB	SampleB_AGL1_ST01_30ng_LIB4.R1.fastq.gz	SampleB_AGL1_ST01_30ng_LIB4.R2.fastq.gz	SampleB_AGL1_ST01_30ng_LIB4.R3.fastq.gz					
Sample B	SAMN16786361	AGL1	SRX9623400	SRR13189020	Illumina NovaSeq 6000	SampleB_AGL1_ST02_30ng_LIB1	PAIRED	fastq		6.95 GB	SampleB_AGL1_ST02_30ng_LIB1.R1.fastq.gz	SampleB_AGL1_ST02_30ng_LIB1.R2.fastq.gz	SampleB_AGL1_ST02_30ng_LIB1.R3.fastq.gz					
Sample B	SAMN16786361	AGL1	SRX9623401	SRR13189019	Illumina NovaSeq 6000	SampleB_AGL1_ST02_30ng_LIB2	PAIRED	fastq		5.15 GB	SampleB_AGL1_ST02_30ng_LIB2.R1.fastq.gz	SampleB_AGL1_ST02_30ng_LIB2.R2.fastq.gz	SampleB_AGL1_ST02_30ng_LIB2.R3.fastq.gz					
Sample B	SAMN16786361	AGL1	SRX9623402	SRR13189018	Illumina NovaSeq 6000	SampleB_AGL1_ST02_30ng_LIB3	PAIRED	fastq		22.22 GB	SampleB_AGL1_ST02_30ng_LIB3.R1.fastq.gz	SampleB_AGL1_ST02_30ng_LIB3.R2.fastq.gz	SampleB_AGL1_ST02_30ng_LIB3.R3.fastq.gz					
Sample B	SAMN16786361	AGL1	SRX9623403	SRR13189017	Illumina NovaSeq 6000	SampleB_AGL1_ST02_30ng_LIB4	PAIRED	fastq		5.59 GB	SampleB_AGL1_ST02_30ng_LIB4.R1.fastq.gz	SampleB_AGL1_ST02_30ng_LIB4.R2.fastq.gz	SampleB_AGL1_ST02_30ng_LIB4.R3.fastq.gz					
Sample B	SAMN16786361	AGL1	SRX14332183	SRR18185260	Illumina NovaSeq 6000	SampleB_AGL1_ST03_30ng_LIB1	PAIRED	fastq		6.64 GB	SampleB_AGL1_ST03_30ng_LIB1_PT1.R1.fastq.gz	SampleB_AGL1_ST03_30ng_LIB1_PT1.R2.fastq.gz	SampleB_AGL1_ST03_30ng_LIB1_PT1.R3.fastq.gz	SampleB_AGL1_ST03_30ng_LIB1_PT2.R1.fastq.gz	SampleB_AGL1_ST03_30ng_LIB1_PT2.R2.fastq.gz	SampleB_AGL1_ST03_30ng_LIB1_PT2.R3.fastq.gz		
Sample B	SAMN16786361	AGL1	SRX14332184	SRR18185259	Illumina NovaSeq 6000	SampleB_AGL1_ST03_30ng_LIB2	PAIRED	fastq		10.58 GB	SampleB_AGL1_ST03_30ng_LIB2_PT1.R1.fastq.gz	SampleB_AGL1_ST03_30ng_LIB2_PT1.R2.fastq.gz	SampleB_AGL1_ST03_30ng_LIB2_PT1.R3.fastq.gz	SampleB_AGL1_ST03_30ng_LIB2_PT2.R1.fastq.gz	SampleB_AGL1_ST03_30ng_LIB2_PT2.R2.fastq.gz	SampleB_AGL1_ST03_30ng_LIB2_PT2.R3.fastq.gz		
Sample B	SAMN16786361	AGL1	SRX14332185	SRR18185258	Illumina NovaSeq 6000	SampleB_AGL1_ST03_30ng_LIB3	PAIRED	fastq		8.03 GB	SampleB_AGL1_ST03_30ng_LIB3_PT1.R1.fastq.gz	SampleB_AGL1_ST03_30ng_LIB3_PT1.R2.fastq.gz	SampleB_AGL1_ST03_30ng_LIB3_PT1.R3.fastq.gz	SampleB_AGL1_ST03_30ng_LIB3_PT2.R1.fastq.gz	SampleB_AGL1_ST03_30ng_LIB3_PT2.R2.fastq.gz	SampleB_AGL1_ST03_30ng_LIB3_PT2.R3.fastq.gz		
Sample B	SAMN16786361	AGL1	SRX14332186	SRR18185257	Illumina NovaSeq 6000	SampleB_AGL1_ST03_30ng_LIB4	PAIRED	fastq		7.59 GB	SampleB_AGL1_ST03_30ng_LIB4_PT1.R1.fastq.gz	SampleB_AGL1_ST03_30ng_LIB4_PT1.R2.fastq.gz	SampleB_AGL1_ST03_30ng_LIB4_PT1.R3.fastq.gz	SampleB_AGL1_ST03_30ng_LIB4_PT2.R1.fastq.gz	SampleB_AGL1_ST03_30ng_LIB4_PT2.R2.fastq.gz	SampleB_AGL1_ST03_30ng_LIB4_PT2.R3.fastq.gz		
Sample C	SAMN16786362	AGL1	SRX9623408	SRR13189012	Illumina NovaSeq 6000	SampleC_AGL1_ST01_30ng_LIB1	PAIRED	fastq		19.15 GB	SampleC_AGL1_ST01_30ng_LIB1.R1.fastq.gz	SampleC_AGL1_ST01_30ng_LIB1.R2.fastq.gz	SampleC_AGL1_ST01_30ng_LIB1.R3.fastq.gz					
Sample C	SAMN16786362	AGL1	SRX9623409	SRR13189011	Illumina NovaSeq 6000	SampleC_AGL1_ST01_30ng_LIB2	PAIRED	fastq		17.56 GB	SampleC_AGL1_ST01_30ng_LIB2.R1.fastq.gz	SampleC_AGL1_ST01_30ng_LIB2.R2.fastq.gz	SampleC_AGL1_ST01_30ng_LIB2.R3.fastq.gz					
Sample C	SAMN16786362	AGL1	SRX9623411	SRR13189009	Illumina NovaSeq 6000	SampleC_AGL1_ST01_30ng_LIB3	PAIRED	fastq		17.46 GB	SampleC_AGL1_ST01_30ng_LIB3.R1.fastq.gz	SampleC_AGL1_ST01_30ng_LIB3.R2.fastq.gz	SampleC_AGL1_ST01_30ng_LIB3.R3.fastq.gz					
Sample C	SAMN16786362	AGL1	SRX9623412	SRR13189008	Illumina NovaSeq 6000	SampleC_AGL1_ST01_30ng_LIB4	PAIRED	fastq		15.68 GB	SampleC_AGL1_ST01_30ng_LIB4.R1.fastq.gz	SampleC_AGL1_ST01_30ng_LIB4.R2.fastq.gz	SampleC_AGL1_ST01_30ng_LIB4.R3.fastq.gz					
Sample C	SAMN16786362	AGL1	SRX9623413	SRR13189007	Illumina NovaSeq 6000	SampleC_AGL1_ST02_30ng_LIB1	PAIRED	fastq		16.19 GB	SampleC_AGL1_ST02_30ng_LIB1.R1.fastq.gz	SampleC_AGL1_ST02_30ng_LIB1.R2.fastq.gz	SampleC_AGL1_ST02_30ng_LIB1.R3.fastq.gz					
Sample C	SAMN16786362	AGL1	SRX9623414	SRR13189006	Illumina NovaSeq 6000	SampleC_AGL1_ST02_30ng_LIB2	PAIRED	fastq		12.30 GB	SampleC_AGL1_ST02_30ng_LIB2.R1.fastq.gz	SampleC_AGL1_ST02_30ng_LIB2.R2.fastq.gz	SampleC_AGL1_ST02_30ng_LIB2.R3.fastq.gz					
Sample C	SAMN16786362	AGL1	SRX9623415	SRR13189005	Illumina NovaSeq 6000	SampleC_AGL1_ST02_30ng_LIB3	PAIRED	fastq		11.16 GB	SampleC_AGL1_ST02_30ng_LIB3.R1.fastq.gz	SampleC_AGL1_ST02_30ng_LIB3.R2.fastq.gz	SampleC_AGL1_ST02_30ng_LIB3.R3.fastq.gz					
Sample C	SAMN16786362	AGL1	SRX9623416	SRR13189004	Illumina NovaSeq 6000	SampleC_AGL1_ST02_30ng_LIB4	PAIRED	fastq		12.04 GB	SampleC_AGL1_ST02_30ng_LIB4.R1.fastq.gz	SampleC_AGL1_ST02_30ng_LIB4.R2.fastq.gz	SampleC_AGL1_ST02_30ng_LIB4.R3.fastq.gz					
Sample C	SAMN16786362	AGL1	SRX14332187	SRR18185256	Illumina NovaSeq 6000	SampleC_AGL1_ST03_30ng_LIB1	PAIRED	fastq		7.43 GB	SampleC_AGL1_ST03_30ng_LIB1_PT1.R1.fastq.gz	SampleC_AGL1_ST03_30ng_LIB1_PT1.R2.fastq.gz	SampleC_AGL1_ST03_30ng_LIB1_PT1.R3.fastq.gz	SampleC_AGL1_ST03_30ng_LIB1_PT2.R1.fastq.gz	SampleC_AGL1_ST03_30ng_LIB1_PT2.R2.fastq.gz	SampleC_AGL1_ST03_30ng_LIB1_PT2.R3.fastq.gz		
Sample C	SAMN16786362	AGL1	SRX14332188	SRR18185255	Illumina NovaSeq 6000	SampleC_AGL1_ST03_30ng_LIB2	PAIRED	fastq		34.39 GB	SampleC_AGL1_ST03_30ng_LIB2_PT1.R1.fastq.gz	SampleC_AGL1_ST03_30ng_LIB2_PT1.R2.fastq.gz	SampleC_AGL1_ST03_30ng_LIB2_PT1.R3.fastq.gz	SampleC_AGL1_ST03_30ng_LIB2_PT2.R1.fastq.gz	SampleC_AGL1_ST03_30ng_LIB2_PT2.R2.fastq.gz	SampleC_AGL1_ST03_30ng_LIB2_PT2.R3.fastq.gz		
Sample C	SAMN16786362	AGL1	SRX14332189	SRR18185254	Illumina NovaSeq 6000	SampleC_AGL1_ST03_30ng_LIB3	PAIRED	fastq		15.32 GB	SampleC_AGL1_ST03_30ng_LIB3_PT1.R1.fastq.gz	SampleC_AGL1_ST03_30ng_LIB3_PT1.R2.fastq.gz	SampleC_AGL1_ST03_30ng_LIB3_PT1.R3.fastq.gz	SampleC_AGL1_ST03_30ng_LIB3_PT2.R1.fastq.gz	SampleC_AGL1_ST03_30ng_LIB3_PT2.R2.fastq.gz	SampleC_AGL1_ST03_30ng_LIB3_PT2.R3.fastq.gz		
Sample C	SAMN16786362	AGL1	SRX14332190	SRR18185253	Illumina NovaSeq 6000	SampleC_AGL1_ST03_30ng_LIB4	PAIRED	fastq		16.25 GB	SampleC_AGL1_ST03_30ng_LIB4_PT1.R1.fastq.gz	SampleC_AGL1_ST03_30ng_LIB4_PT1.R2.fastq.gz	SampleC_AGL1_ST03_30ng_LIB4_PT1.R3.fastq.gz	SampleC_AGL1_ST03_30ng_LIB4_PT2.R1.fastq.gz	SampleC_AGL1_ST03_30ng_LIB4_PT2.R2.fastq.gz	SampleC_AGL1_ST03_30ng_LIB4_PT2.R3.fastq.gz		
Sample A	SAMN16786360	BRP1	SRX9605667	SRR13167117	Illumina NovaSeq 6000	SampleA_BRP1_ST25_100ng_LIB1	PAIRED	fastq		4.00 GB	SampleA_BRP1_ST25_100ng_LIB1.R1.fastq.gz	SampleA_BRP1_ST25_100ng_LIB1.R2.fastq.gz						
Sample A	SAMN16786360	BRP1	SRX9605668	SRR13167116	Illumina NovaSeq 6000	SampleA_BRP1_ST25_100ng_LIB2	PAIRED	fastq		4.89 GB	SampleA_BRP1_ST25_100ng_LIB2.R1.fastq.gz	SampleA_BRP1_ST25_100ng_LIB2.R2.fastq.gz						
Sample A	SAMN16786360	BRP1	SRX9605679	SRR13167105	Illumina NovaSeq 6000	SampleA_BRP1_ST25_100ng_LIB3	PAIRED	fastq		3.68 GB	SampleA_BRP1_ST25_100ng_LIB3.R1.fastq.gz	SampleA_BRP1_ST25_100ng_LIB3.R2.fastq.gz						
Sample A	SAMN16786360	BRP1	SRX9605690	SRR13167094	Illumina NovaSeq 6000	SampleA_BRP1_ST25_100ng_LIB4	PAIRED	fastq		5.33 GB	SampleA_BRP1_ST25_100ng_LIB4.R1.fastq.gz	SampleA_BRP1_ST25_100ng_LIB4.R2.fastq.gz						
Sample A	SAMN16786360	BRP1	SRX9605701	SRR13167083	Illumina NovaSeq 6000	SampleA_BRP1_ST26_100ng_LIB1	PAIRED	fastq		4.82 GB	SampleA_BRP1_ST26_100ng_LIB1.R1.fastq.gz	SampleA_BRP1_ST26_100ng_LIB1.R2.fastq.gz						
Sample A	SAMN16786360	BRP1	SRX9605712	SRR13167072	Illumina NovaSeq 6000	SampleA_BRP1_ST26_100ng_LIB2	PAIRED	fastq		4.78 GB	SampleA_BRP1_ST26_100ng_LIB2.R1.fastq.gz	SampleA_BRP1_ST26_100ng_LIB2.R2.fastq.gz						
Sample A	SAMN16786360	BRP1	SRX9605723	SRR13167061	Illumina NovaSeq 6000	SampleA_BRP1_ST26_100ng_LIB3	PAIRED	fastq		3.68 GB	SampleA_BRP1_ST26_100ng_LIB3.R1.fastq.gz	SampleA_BRP1_ST26_100ng_LIB3.R2.fastq.gz						
Sample A	SAMN16786360	BRP1	SRX9605728	SRR13167056	Illumina NovaSeq 6000	SampleA_BRP1_ST26_100ng_LIB4	PAIRED	fastq		3.80 GB	SampleA_BRP1_ST26_100ng_LIB4.R1.fastq.gz	SampleA_BRP1_ST26_100ng_LIB4.R2.fastq.gz						
Sample A	SAMN16786360	BRP1	SRX9605729	SRR13167055	Illumina NovaSeq 6000	SampleA_BRP1_ST27_100ng_LIB1	PAIRED	fastq		5.44 GB	SampleA_BRP1_ST27_100ng_LIB1.R1.fastq.gz	SampleA_BRP1_ST27_100ng_LIB1.R2.fastq.gz						
Sample A	SAMN16786360	BRP1	SRX9605730	SRR13167054	Illumina NovaSeq 6000	SampleA_BRP1_ST27_100ng_LIB2	PAIRED	fastq		5.04 GB	SampleA_BRP1_ST27_100ng_LIB2.R1.fastq.gz	SampleA_BRP1_ST27_100ng_LIB2.R2.fastq.gz						
Sample A	SAMN16786360	BRP1	SRX9605669	SRR13167115	Illumina NovaSeq 6000	SampleA_BRP1_ST27_100ng_LIB3	PAIRED	fastq		4.61 GB	SampleA_BRP1_ST27_100ng_LIB3.R1.fastq.gz	SampleA_BRP1_ST27_100ng_LIB3.R2.fastq.gz						
Sample A	SAMN16786360	BRP1	SRX9605670	SRR13167114	Illumina NovaSeq 6000	SampleA_BRP1_ST27_100ng_LIB4	PAIRED	fastq		4.21 GB	SampleA_BRP1_ST27_100ng_LIB4.R1.fastq.gz	SampleA_BRP1_ST27_100ng_LIB4.R2.fastq.gz						
Sample A	SAMN16786360	BRP1	SRX9605671	SRR13167113	Illumina NovaSeq 6000	SampleA_BRP1_ST28_100ng_LIB1	PAIRED	fastq		4.60 GB	SampleA_BRP1_ST28_100ng_LIB1.R1.fastq.gz	SampleA_BRP1_ST28_100ng_LIB1.R2.fastq.gz						
Sample A	SAMN16786360	BRP1	SRX9605672	SRR13167112	Illumina NovaSeq 6000	SampleA_BRP1_ST28_100ng_LIB2	PAIRED	fastq		4.41 GB	SampleA_BRP1_ST28_100ng_LIB2.R1.fastq.gz	SampleA_BRP1_ST28_100ng_LIB2.R2.fastq.gz						
Sample A	SAMN16786360	BRP1	SRX9605673	SRR13167111	Illumina NovaSeq 6000	SampleA_BRP1_ST28_100ng_LIB3	PAIRED	fastq		3.64 GB	SampleA_BRP1_ST28_100ng_LIB3.R1.fastq.gz	SampleA_BRP1_ST28_100ng_LIB3.R2.fastq.gz						
Sample A	SAMN16786360	BRP1	SRX9605674	SRR13167110	Illumina NovaSeq 6000	SampleA_BRP1_ST28_100ng_LIB4	PAIRED	fastq		3.45 GB	SampleA_BRP1_ST28_100ng_LIB4.R1.fastq.gz	SampleA_BRP1_ST28_100ng_LIB4.R2.fastq.gz						
Sample Spike-in	SAMN16786363	BRP1	SRX9605675	SRR13167109	Illumina NovaSeq 6000	SampleAC5_BRP1_ST25_100ng_LIB1	PAIRED	fastq		4.50 GB	SampleAC5_BRP1_ST25_100ng_LIB1.R1.fastq.gz	SampleAC5_BRP1_ST25_100ng_LIB1.R2.fastq.gz						
Sample Spike-in	SAMN16786363	BRP1	SRX9605676	SRR13167108	Illumina NovaSeq 6000	SampleAC5_BRP1_ST25_100ng_LIB2	PAIRED	fastq		4.19 GB	SampleAC5_BRP1_ST25_100ng_LIB2.R1.fastq.gz	SampleAC5_BRP1_ST25_100ng_LIB2.R2.fastq.gz						
Sample Spike-in	SAMN16786363	BRP1	SRX9605677	SRR13167107	Illumina NovaSeq 6000	SampleAC5_BRP1_ST25_100ng_LIB3	PAIRED	fastq		3.57 GB	SampleAC5_BRP1_ST25_100ng_LIB3.R1.fastq.gz	SampleAC5_BRP1_ST25_100ng_LIB3.R2.fastq.gz						
Sample Spike-in	SAMN16786363	BRP1	SRX9605678	SRR13167106	Illumina NovaSeq 6000	SampleAC5_BRP1_ST25_100ng_LIB4	PAIRED	fastq		3.50 GB	SampleAC5_BRP1_ST25_100ng_LIB4.R1.fastq.gz	SampleAC5_BRP1_ST25_100ng_LIB4.R2.fastq.gz						
Sample Spike-in	SAMN16786363	BRP1	SRX9605680	SRR13167104	Illumina NovaSeq 6000	SampleAC5_BRP1_ST26_100ng_LIB1	PAIRED	fastq		6.71 GB	SampleAC5_BRP1_ST26_100ng_LIB1.R1.fastq.gz	SampleAC5_BRP1_ST26_100ng_LIB1.R2.fastq.gz						
Sample Spike-in	SAMN16786363	BRP1	SRX9605681	SRR13167103	Illumina NovaSeq 6000	SampleAC5_BRP1_ST26_100ng_LIB2	PAIRED	fastq		6.70 GB	SampleAC5_BRP1_ST26_100ng_LIB2.R1.fastq.gz	SampleAC5_BRP1_ST26_100ng_LIB2.R2.fastq.gz						
Sample Spike-in	SAMN16786363	BRP1	SRX9605682	SRR13167102	Illumina NovaSeq 6000	SampleAC5_BRP1_ST26_100ng_LIB3	PAIRED	fastq		5.21 GB	SampleAC5_BRP1_ST26_100ng_LIB3.R1.fastq.gz	SampleAC5_BRP1_ST26_100ng_LIB3.R2.fastq.gz						
Sample Spike-in	SAMN16786363	BRP1	SRX9605683	SRR13167101	Illumina NovaSeq 6000	SampleAC5_BRP1_ST26_100ng_LIB4	PAIRED	fastq		5.10 GB	SampleAC5_BRP1_ST26_100ng_LIB4.R1.fastq.gz	SampleAC5_BRP1_ST26_100ng_LIB4.R2.fastq.gz						
Sample Spike-in	SAMN16786363	BRP1	SRX9605684	SRR13167100	Illumina NovaSeq 6000	SampleAC5_BRP1_ST27_100ng_LIB1	PAIRED	fastq		4.93 GB	SampleAC5_BRP1_ST27_100ng_LIB1.R1.fastq.gz	SampleAC5_BRP1_ST27_100ng_LIB1.R2.fastq.gz						
Sample Spike-in	SAMN16786363	BRP1	SRX9605685	SRR13167099	Illumina NovaSeq 6000	SampleAC5_BRP1_ST27_100ng_LIB2	PAIRED	fastq		4.10 GB	SampleAC5_BRP1_ST27_100ng_LIB2.R1.fastq.gz	SampleAC5_BRP1_ST27_100ng_LIB2.R2.fastq.gz						
Sample Spike-in	SAMN16786363	BRP1	SRX9605686	SRR13167098	Illumina NovaSeq 6000	SampleAC5_BRP1_ST27_100ng_LIB3	PAIRED	fastq		4.63 GB	SampleAC5_BRP1_ST27_100ng_LIB3.R1.fastq.gz	SampleAC5_BRP1_ST27_100ng_LIB3.R2.fastq.gz						
Sample Spike-in	SAMN16786363	BRP1	SRX9605687	SRR13167097	Illumina NovaSeq 6000	SampleAC5_BRP1_ST27_100ng_LIB4	PAIRED	fastq		4.57 GB	SampleAC5_BRP1_ST27_100ng_LIB4.R1.fastq.gz	SampleAC5_BRP1_ST27_100ng_LIB4.R2.fastq.gz						
Sample Spike-in	SAMN16786363	BRP1	SRX9605688	SRR13167096	Illumina NovaSeq 6000	SampleAC5_BRP1_ST28_100ng_LIB1	PAIRED	fastq		5.03 GB	SampleAC5_BRP1_ST28_100ng_LIB1.R1.fastq.gz	SampleAC5_BRP1_ST28_100ng_LIB1.R2.fastq.gz						
Sample Spike-in	SAMN16786363	BRP1	SRX9605689	SRR13167095	Illumina NovaSeq 6000	SampleAC5_BRP1_ST28_100ng_LIB2	PAIRED	fastq		3.62 GB	SampleAC5_BRP1_ST28_100ng_LIB2.R1.fastq.gz	SampleAC5_BRP1_ST28_100ng_LIB2.R2.fastq.gz						
Sample Spike-in	SAMN16786363	BRP1	SRX9605691	SRR13167093	Illumina NovaSeq 6000	SampleAC5_BRP1_ST28_100ng_LIB3	PAIRED	fastq		2.54 GB	SampleAC5_BRP1_ST28_100ng_LIB3.R1.fastq.gz	SampleAC5_BRP1_ST28_100ng_LIB3.R2.fastq.gz						
Sample Spike-in	SAMN16786363	BRP1	SRX9605692	SRR13167092	Illumina NovaSeq 6000	SampleAC5_BRP1_ST28_100ng_LIB4	PAIRED	fastq		3.02 GB	SampleAC5_BRP1_ST28_100ng_LIB4.R1.fastq.gz	SampleAC5_BRP1_ST28_100ng_LIB4.R2.fastq.gz						
Sample B	SAMN16786361	BRP1	SRX9605693	SRR13167091	Illumina NovaSeq 6000	SampleB_BRP1_ST25_100ng_LIB1	PAIRED	fastq		3.76 GB	SampleB_BRP1_ST25_100ng_LIB1.R1.fastq.gz	SampleB_BRP1_ST25_100ng_LIB1.R2.fastq.gz						
Sample B	SAMN16786361	BRP1	SRX9605694	SRR13167090	Illumina NovaSeq 6000	SampleB_BRP1_ST25_100ng_LIB2	PAIRED	fastq		4.25 GB	SampleB_BRP1_ST25_100ng_LIB2.R1.fastq.gz	SampleB_BRP1_ST25_100ng_LIB2.R2.fastq.gz						
Sample B	SAMN16786361	BRP1	SRX9605695	SRR13167089	Illumina NovaSeq 6000	SampleB_BRP1_ST25_100ng_LIB3	PAIRED	fastq		5.81 GB	SampleB_BRP1_ST25_100ng_LIB3.R1.fastq.gz	SampleB_BRP1_ST25_100ng_LIB3.R2.fastq.gz						
Sample B	SAMN16786361	BRP1	SRX9605696	SRR13167088	Illumina NovaSeq 6000	SampleB_BRP1_ST25_100ng_LIB4	PAIRED	fastq		5.91 GB	SampleB_BRP1_ST25_100ng_LIB4.R1.fastq.gz	SampleB_BRP1_ST25_100ng_LIB4.R2.fastq.gz						
Sample B	SAMN16786361	BRP1	SRX9605697	SRR13167087	Illumina NovaSeq 6000	SampleB_BRP1_ST26_100ng_LIB1	PAIRED	fastq		4.98 GB	SampleB_BRP1_ST26_100ng_LIB1.R1.fastq.gz	SampleB_BRP1_ST26_100ng_LIB1.R2.fastq.gz						
Sample B	SAMN16786361	BRP1	SRX9605698	SRR13167086	Illumina NovaSeq 6000	SampleB_BRP1_ST26_100ng_LIB2	PAIRED	fastq		5.06 GB	SampleB_BRP1_ST26_100ng_LIB2.R1.fastq.gz	SampleB_BRP1_ST26_100ng_LIB2.R2.fastq.gz						
Sample B	SAMN16786361	BRP1	SRX9605699	SRR13167085	Illumina NovaSeq 6000	SampleB_BRP1_ST26_100ng_LIB3	PAIRED	fastq		5.47 GB	SampleB_BRP1_ST26_100ng_LIB3.R1.fastq.gz	SampleB_BRP1_ST26_100ng_LIB3.R2.fastq.gz						
Sample B	SAMN16786361	BRP1	SRX9605700	SRR13167084	Illumina NovaSeq 6000	SampleB_BRP1_ST26_100ng_LIB4	PAIRED	fastq		4.52 GB	SampleB_BRP1_ST26_100ng_LIB4.R1.fastq.gz	SampleB_BRP1_ST26_100ng_LIB4.R2.fastq.gz						
Sample B	SAMN16786361	BRP1	SRX9605702	SRR13167082	Illumina NovaSeq 6000	SampleB_BRP1_ST27_100ng_LIB1	PAIRED	fastq		4.36 GB	SampleB_BRP1_ST27_100ng_LIB1.R1.fastq.gz	SampleB_BRP1_ST27_100ng_LIB1.R2.fastq.gz						
Sample B	SAMN16786361	BRP1	SRX9605703	SRR13167081	Illumina NovaSeq 6000	SampleB_BRP1_ST27_100ng_LIB2	PAIRED	fastq		5.00 GB	SampleB_BRP1_ST27_100ng_LIB2.R1.fastq.gz	SampleB_BRP1_ST27_100ng_LIB2.R2.fastq.gz						
Sample B	SAMN16786361	BRP1	SRX9605704	SRR13167080	Illumina NovaSeq 6000	SampleB_BRP1_ST27_100ng_LIB3	PAIRED	fastq		4.54 GB	SampleB_BRP1_ST27_100ng_LIB3.R1.fastq.gz	SampleB_BRP1_ST27_100ng_LIB3.R2.fastq.gz						
Sample B	SAMN16786361	BRP1	SRX9605705	SRR13167079	Illumina NovaSeq 6000	SampleB_BRP1_ST27_100ng_LIB4	PAIRED	fastq		4.67 GB	SampleB_BRP1_ST27_100ng_LIB4.R1.fastq.gz	SampleB_BRP1_ST27_100ng_LIB4.R2.fastq.gz						
Sample B	SAMN16786361	BRP1	SRX9605706	SRR13167078	Illumina NovaSeq 6000	SampleB_BRP1_ST28_100ng_LIB1	PAIRED	fastq		4.93 GB	SampleB_BRP1_ST28_100ng_LIB1.R1.fastq.gz	SampleB_BRP1_ST28_100ng_LIB1.R2.fastq.gz						
Sample B	SAMN16786361	BRP1	SRX9605707	SRR13167077	Illumina NovaSeq 6000	SampleB_BRP1_ST28_100ng_LIB2	PAIRED	fastq		5.05 GB	SampleB_BRP1_ST28_100ng_LIB2.R1.fastq.gz	SampleB_BRP1_ST28_100ng_LIB2.R2.fastq.gz						
Sample B	SAMN16786361	BRP1	SRX9605708	SRR13167076	Illumina NovaSeq 6000	SampleB_BRP1_ST28_100ng_LIB3	PAIRED	fastq		3.91 GB	SampleB_BRP1_ST28_100ng_LIB3.R1.fastq.gz	SampleB_BRP1_ST28_100ng_LIB3.R2.fastq.gz						
Sample B	SAMN16786361	BRP1	SRX9605709	SRR13167075	Illumina NovaSeq 6000	SampleB_BRP1_ST28_100ng_LIB4	PAIRED	fastq		3.03 GB	SampleB_BRP1_ST28_100ng_LIB4.R1.fastq.gz	SampleB_BRP1_ST28_100ng_LIB4.R2.fastq.gz						
Sample C	SAMN16786362	BRP1	SRX9605710	SRR13167074	Illumina NovaSeq 6000	SampleC_BRP1_ST25_100ng_LIB1	PAIRED	fastq		3.65 GB	SampleC_BRP1_ST25_100ng_LIB1.R1.fastq.gz	SampleC_BRP1_ST25_100ng_LIB1.R2.fastq.gz						
Sample C	SAMN16786362	BRP1	SRX9605711	SRR13167073	Illumina NovaSeq 6000	SampleC_BRP1_ST25_100ng_LIB2	PAIRED	fastq		4.23 GB	SampleC_BRP1_ST25_100ng_LIB2.R1.fastq.gz	SampleC_BRP1_ST25_100ng_LIB2.R2.fastq.gz						
Sample C	SAMN16786362	BRP1	SRX9605713	SRR13167071	Illumina NovaSeq 6000	SampleC_BRP1_ST25_100ng_LIB3	PAIRED	fastq		4.58 GB	SampleC_BRP1_ST25_100ng_LIB3.R1.fastq.gz	SampleC_BRP1_ST25_100ng_LIB3.R2.fastq.gz						
Sample C	SAMN16786362	BRP1	SRX9605714	SRR13167070	Illumina NovaSeq 6000	SampleC_BRP1_ST25_100ng_LIB4	PAIRED	fastq		4.34 GB	SampleC_BRP1_ST25_100ng_LIB4.R1.fastq.gz	SampleC_BRP1_ST25_100ng_LIB4.R2.fastq.gz						
Sample C	SAMN16786362	BRP1	SRX9605715	SRR13167069	Illumina NovaSeq 6000	SampleC_BRP1_ST26_100ng_LIB1	PAIRED	fastq		6.87 GB	SampleC_BRP1_ST26_100ng_LIB1.R1.fastq.gz	SampleC_BRP1_ST26_100ng_LIB1.R2.fastq.gz						
Sample C	SAMN16786362	BRP1	SRX9605716	SRR13167068	Illumina NovaSeq 6000	SampleC_BRP1_ST26_100ng_LIB2	PAIRED	fastq		7.69 GB	SampleC_BRP1_ST26_100ng_LIB2.R1.fastq.gz	SampleC_BRP1_ST26_100ng_LIB2.R2.fastq.gz						
Sample C	SAMN16786362	BRP1	SRX9605717	SRR13167067	Illumina NovaSeq 6000	SampleC_BRP1_ST26_100ng_LIB3	PAIRED	fastq		7.83 GB	SampleC_BRP1_ST26_100ng_LIB3.R1.fastq.gz	SampleC_BRP1_ST26_100ng_LIB3.R2.fastq.gz						
Sample C	SAMN16786362	BRP1	SRX9605718	SRR13167066	Illumina NovaSeq 6000	SampleC_BRP1_ST26_100ng_LIB4	PAIRED	fastq		4.74 GB	SampleC_BRP1_ST26_100ng_LIB4.R1.fastq.gz	SampleC_BRP1_ST26_100ng_LIB4.R2.fastq.gz						
Sample C	SAMN16786362	BRP1	SRX9605719	SRR13167065	Illumina NovaSeq 6000	SampleC_BRP1_ST27_100ng_LIB1	PAIRED	fastq		5.19 GB	SampleC_BRP1_ST27_100ng_LIB1.R1.fastq.gz	SampleC_BRP1_ST27_100ng_LIB1.R2.fastq.gz						
Sample C	SAMN16786362	BRP1	SRX9605720	SRR13167064	Illumina NovaSeq 6000	SampleC_BRP1_ST27_100ng_LIB2	PAIRED	fastq		3.78 GB	SampleC_BRP1_ST27_100ng_LIB2.R1.fastq.gz	SampleC_BRP1_ST27_100ng_LIB2.R2.fastq.gz						
Sample C	SAMN16786362	BRP1	SRX9605721	SRR13167063	Illumina NovaSeq 6000	SampleC_BRP1_ST27_100ng_LIB3	PAIRED	fastq		4.11 GB	SampleC_BRP1_ST27_100ng_LIB3.R1.fastq.gz	SampleC_BRP1_ST27_100ng_LIB3.R2.fastq.gz						
Sample C	SAMN16786362	BRP1	SRX9605722	SRR13167062	Illumina NovaSeq 6000	SampleC_BRP1_ST27_100ng_LIB4	PAIRED	fastq		4.42 GB	SampleC_BRP1_ST27_100ng_LIB4.R1.fastq.gz	SampleC_BRP1_ST27_100ng_LIB4.R2.fastq.gz						
Sample C	SAMN16786362	BRP1	SRX9605724	SRR13167060	Illumina NovaSeq 6000	SampleC_BRP1_ST28_100ng_LIB1	PAIRED	fastq		4.34 GB	SampleC_BRP1_ST28_100ng_LIB1.R1.fastq.gz	SampleC_BRP1_ST28_100ng_LIB1.R2.fastq.gz						
Sample C	SAMN16786362	BRP1	SRX9605725	SRR13167059	Illumina NovaSeq 6000	SampleC_BRP1_ST28_100ng_LIB2	PAIRED	fastq		3.60 GB	SampleC_BRP1_ST28_100ng_LIB2.R1.fastq.gz	SampleC_BRP1_ST28_100ng_LIB2.R2.fastq.gz						
Sample C	SAMN16786362	BRP1	SRX9605726	SRR13167058	Illumina NovaSeq 6000	SampleC_BRP1_ST28_100ng_LIB3	PAIRED	fastq		2.85 GB	SampleC_BRP1_ST28_100ng_LIB3.R1.fastq.gz	SampleC_BRP1_ST28_100ng_LIB3.R2.fastq.gz						
Sample C	SAMN16786362	BRP1	SRX9605727	SRR13167057	Illumina NovaSeq 6000	SampleC_BRP1_ST28_100ng_LIB4	PAIRED	fastq		3.94 GB	SampleC_BRP1_ST28_100ng_LIB4.R1.fastq.gz	SampleC_BRP1_ST28_100ng_LIB4.R2.fastq.gz						
Sample A	SAMN16786360	IDT1	SRX9672606	SRR13240841	Illumina NovaSeq 6000	SampleA_IDT1_ST04_100ng_LIB1	PAIRED	fastq		5.91 GB	SampleA_IDT1_ST04_100ng_LIB1.R1.fastq.gz	SampleA_IDT1_ST04_100ng_LIB1.R2.fastq.gz						
Sample A	SAMN16786360	IDT1	SRX9672607	SRR13240840	Illumina NovaSeq 6000	SampleA_IDT1_ST04_100ng_LIB2	PAIRED	fastq		6.11 GB	SampleA_IDT1_ST04_100ng_LIB2.R1.fastq.gz	SampleA_IDT1_ST04_100ng_LIB2.R2.fastq.gz						
Sample A	SAMN16786360	IDT1	SRX9672618	SRR13240829	Illumina NovaSeq 6000	SampleA_IDT1_ST04_100ng_LIB3	PAIRED	fastq		6.10 GB	SampleA_IDT1_ST04_100ng_LIB3.R1.fastq.gz	SampleA_IDT1_ST04_100ng_LIB3.R2.fastq.gz						
Sample A	SAMN16786360	IDT1	SRX9672646	SRR13240801	Illumina NovaSeq 6000	SampleA_IDT1_ST04_100ng_LIB4	PAIRED	fastq		6.09 GB	SampleA_IDT1_ST04_100ng_LIB4.R1.fastq.gz	SampleA_IDT1_ST04_100ng_LIB4.R2.fastq.gz						
Sample A	SAMN16786360	IDT1	SRX9672619	SRR13240828	Illumina NovaSeq 6000	SampleA_IDT1_ST05_100ng_LIB1	PAIRED	fastq		5.96 GB	SampleA_IDT1_ST05_100ng_LIB1.R1.fastq.gz	SampleA_IDT1_ST05_100ng_LIB1.R2.fastq.gz						
Sample A	SAMN16786360	IDT1	SRX9672654	SRR13240793	Illumina NovaSeq 6000	SampleA_IDT1_ST05_100ng_LIB2	PAIRED	fastq		6.10 GB	SampleA_IDT1_ST05_100ng_LIB2.R1.fastq.gz	SampleA_IDT1_ST05_100ng_LIB2.R2.fastq.gz						
Sample A	SAMN16786360	IDT1	SRX9672653	SRR13240794	Illumina NovaSeq 6000	SampleA_IDT1_ST05_100ng_LIB3	PAIRED	fastq		6.13 GB	SampleA_IDT1_ST05_100ng_LIB3.R1.fastq.gz	SampleA_IDT1_ST05_100ng_LIB3.R2.fastq.gz						
Sample A	SAMN16786360	IDT1	SRX9672620	SRR13240827	Illumina NovaSeq 6000	SampleA_IDT1_ST05_100ng_LIB4	PAIRED	fastq		6.13 GB	SampleA_IDT1_ST05_100ng_LIB4.R1.fastq.gz	SampleA_IDT1_ST05_100ng_LIB4.R2.fastq.gz						
Sample A	SAMN16786360	IDT1	SRX9672634	SRR13240813	Illumina NovaSeq 6000	SampleA_IDT1_ST06_100ng_LIB1	PAIRED	fastq		6.11 GB	SampleA_IDT1_ST06_100ng_LIB1.R1.fastq.gz	SampleA_IDT1_ST06_100ng_LIB1.R2.fastq.gz						
Sample A	SAMN16786360	IDT1	SRX9672633	SRR13240814	Illumina NovaSeq 6000	SampleA_IDT1_ST06_100ng_LIB2	PAIRED	fastq		6.15 GB	SampleA_IDT1_ST06_100ng_LIB2.R1.fastq.gz	SampleA_IDT1_ST06_100ng_LIB2.R2.fastq.gz						
Sample A	SAMN16786360	IDT1	SRX9672632	SRR13240815	Illumina NovaSeq 6000	SampleA_IDT1_ST06_100ng_LIB3	PAIRED	fastq		6.14 GB	SampleA_IDT1_ST06_100ng_LIB3.R1.fastq.gz	SampleA_IDT1_ST06_100ng_LIB3.R2.fastq.gz						
Sample A	SAMN16786360	IDT1	SRX9672621	SRR13240826	Illumina NovaSeq 6000	SampleA_IDT1_ST06_100ng_LIB4	PAIRED	fastq		6.15 GB	SampleA_IDT1_ST06_100ng_LIB4.R1.fastq.gz	SampleA_IDT1_ST06_100ng_LIB4.R2.fastq.gz						
Sample Spike-in	SAMN16786363	IDT1	SRX9672656	SRR13240791	Illumina NovaSeq 6000	SampleAC5_IDT1_ST04_100ng_LIB1	PAIRED	fastq		6.08 GB	SampleAC5_IDT1_ST04_100ng_LIB1.R1.fastq.gz	SampleAC5_IDT1_ST04_100ng_LIB1.R2.fastq.gz						
Sample Spike-in	SAMN16786363	IDT1	SRX9672655	SRR13240792	Illumina NovaSeq 6000	SampleAC5_IDT1_ST04_100ng_LIB2	PAIRED	fastq		6.02 GB	SampleAC5_IDT1_ST04_100ng_LIB2.R1.fastq.gz	SampleAC5_IDT1_ST04_100ng_LIB2.R2.fastq.gz						
Sample Spike-in	SAMN16786363	IDT1	SRX9672608	SRR13240839	Illumina NovaSeq 6000	SampleAC5_IDT1_ST04_100ng_LIB3	PAIRED	fastq		5.93 GB	SampleAC5_IDT1_ST04_100ng_LIB3.R1.fastq.gz	SampleAC5_IDT1_ST04_100ng_LIB3.R2.fastq.gz						
Sample Spike-in	SAMN16786363	IDT1	SRX9672609	SRR13240838	Illumina NovaSeq 6000	SampleAC5_IDT1_ST04_100ng_LIB4	PAIRED	fastq		6.07 GB	SampleAC5_IDT1_ST04_100ng_LIB4.R1.fastq.gz	SampleAC5_IDT1_ST04_100ng_LIB4.R2.fastq.gz						
Sample Spike-in	SAMN16786363	IDT1	SRX9672648	SRR13240799	Illumina NovaSeq 6000	SampleAC5_IDT1_ST05_100ng_LIB1	PAIRED	fastq		6.15 GB	SampleAC5_IDT1_ST05_100ng_LIB1.R1.fastq.gz	SampleAC5_IDT1_ST05_100ng_LIB1.R2.fastq.gz						
Sample Spike-in	SAMN16786363	IDT1	SRX9672647	SRR13240800	Illumina NovaSeq 6000	SampleAC5_IDT1_ST05_100ng_LIB2	PAIRED	fastq		6.14 GB	SampleAC5_IDT1_ST05_100ng_LIB2.R1.fastq.gz	SampleAC5_IDT1_ST05_100ng_LIB2.R2.fastq.gz						
Sample Spike-in	SAMN16786363	IDT1	SRX9672645	SRR13240802	Illumina NovaSeq 6000	SampleAC5_IDT1_ST05_100ng_LIB3	PAIRED	fastq		6.09 GB	SampleAC5_IDT1_ST05_100ng_LIB3.R1.fastq.gz	SampleAC5_IDT1_ST05_100ng_LIB3.R2.fastq.gz						
Sample Spike-in	SAMN16786363	IDT1	SRX9672644	SRR13240803	Illumina NovaSeq 6000	SampleAC5_IDT1_ST05_100ng_LIB4	PAIRED	fastq		6.12 GB	SampleAC5_IDT1_ST05_100ng_LIB4.R1.fastq.gz	SampleAC5_IDT1_ST05_100ng_LIB4.R2.fastq.gz						
Sample Spike-in	SAMN16786363	IDT1	SRX9672622	SRR13240825	Illumina NovaSeq 6000	SampleAC5_IDT1_ST06_100ng_LIB1	PAIRED	fastq		6.19 GB	SampleAC5_IDT1_ST06_100ng_LIB1.R1.fastq.gz	SampleAC5_IDT1_ST06_100ng_LIB1.R2.fastq.gz						
Sample Spike-in	SAMN16786363	IDT1	SRX9672665	SRR13240782	Illumina NovaSeq 6000	SampleAC5_IDT1_ST06_100ng_LIB2	PAIRED	fastq		6.12 GB	SampleAC5_IDT1_ST06_100ng_LIB2.R1.fastq.gz	SampleAC5_IDT1_ST06_100ng_LIB2.R2.fastq.gz						
Sample Spike-in	SAMN16786363	IDT1	SRX9672626	SRR13240821	Illumina NovaSeq 6000	SampleAC5_IDT1_ST06_100ng_LIB3	PAIRED	fastq		6.09 GB	SampleAC5_IDT1_ST06_100ng_LIB3.R1.fastq.gz	SampleAC5_IDT1_ST06_100ng_LIB3.R2.fastq.gz						
Sample Spike-in	SAMN16786363	IDT1	SRX9672625	SRR13240822	Illumina NovaSeq 6000	SampleAC5_IDT1_ST06_100ng_LIB4	PAIRED	fastq		6.19 GB	SampleAC5_IDT1_ST06_100ng_LIB4.R1.fastq.gz	SampleAC5_IDT1_ST06_100ng_LIB4.R2.fastq.gz						
Sample B	SAMN16786361	IDT1	SRX9672610	SRR13240837	Illumina NovaSeq 6000	SampleB_IDT1_ST04_100ng_LIB1	PAIRED	fastq		6.04 GB	SampleB_IDT1_ST04_100ng_LIB1.R1.fastq.gz	SampleB_IDT1_ST04_100ng_LIB1.R2.fastq.gz						
Sample B	SAMN16786361	IDT1	SRX9672611	SRR13240836	Illumina NovaSeq 6000	SampleB_IDT1_ST04_100ng_LIB2	PAIRED	fastq		6.09 GB	SampleB_IDT1_ST04_100ng_LIB2.R1.fastq.gz	SampleB_IDT1_ST04_100ng_LIB2.R2.fastq.gz						
Sample B	SAMN16786361	IDT1	SRX9672612	SRR13240835	Illumina NovaSeq 6000	SampleB_IDT1_ST04_100ng_LIB3	PAIRED	fastq		6.11 GB	SampleB_IDT1_ST04_100ng_LIB3.R1.fastq.gz	SampleB_IDT1_ST04_100ng_LIB3.R2.fastq.gz						
Sample B	SAMN16786361	IDT1	SRX9672613	SRR13240834	Illumina NovaSeq 6000	SampleB_IDT1_ST04_100ng_LIB4	PAIRED	fastq		6.06 GB	SampleB_IDT1_ST04_100ng_LIB4.R1.fastq.gz	SampleB_IDT1_ST04_100ng_LIB4.R2.fastq.gz						
Sample B	SAMN16786361	IDT1	SRX9672643	SRR13240804	Illumina NovaSeq 6000	SampleB_IDT1_ST05_100ng_LIB1	PAIRED	fastq		6.07 GB	SampleB_IDT1_ST05_100ng_LIB1.R1.fastq.gz	SampleB_IDT1_ST05_100ng_LIB1.R2.fastq.gz						
Sample B	SAMN16786361	IDT1	SRX9672642	SRR13240805	Illumina NovaSeq 6000	SampleB_IDT1_ST05_100ng_LIB2	PAIRED	fastq		6.11 GB	SampleB_IDT1_ST05_100ng_LIB2.R1.fastq.gz	SampleB_IDT1_ST05_100ng_LIB2.R2.fastq.gz						
Sample B	SAMN16786361	IDT1	SRX9672641	SRR13240806	Illumina NovaSeq 6000	SampleB_IDT1_ST05_100ng_LIB3	PAIRED	fastq		6.12 GB	SampleB_IDT1_ST05_100ng_LIB3.R1.fastq.gz	SampleB_IDT1_ST05_100ng_LIB3.R2.fastq.gz						
Sample B	SAMN16786361	IDT1	SRX9672640	SRR13240807	Illumina NovaSeq 6000	SampleB_IDT1_ST05_100ng_LIB4	PAIRED	fastq		6.06 GB	SampleB_IDT1_ST05_100ng_LIB4.R1.fastq.gz	SampleB_IDT1_ST05_100ng_LIB4.R2.fastq.gz						
Sample B	SAMN16786361	IDT1	SRX9672624	SRR13240823	Illumina NovaSeq 6000	SampleB_IDT1_ST06_100ng_LIB1	PAIRED	fastq		6.14 GB	SampleB_IDT1_ST06_100ng_LIB1.R1.fastq.gz	SampleB_IDT1_ST06_100ng_LIB1.R2.fastq.gz						
Sample B	SAMN16786361	IDT1	SRX9672623	SRR13240824	Illumina NovaSeq 6000	SampleB_IDT1_ST06_100ng_LIB2	PAIRED	fastq		6.10 GB	SampleB_IDT1_ST06_100ng_LIB2.R1.fastq.gz	SampleB_IDT1_ST06_100ng_LIB2.R2.fastq.gz						
Sample B	SAMN16786361	IDT1	SRX9672664	SRR13240783	Illumina NovaSeq 6000	SampleB_IDT1_ST06_100ng_LIB3	PAIRED	fastq		6.15 GB	SampleB_IDT1_ST06_100ng_LIB3.R1.fastq.gz	SampleB_IDT1_ST06_100ng_LIB3.R2.fastq.gz						
Sample B	SAMN16786361	IDT1	SRX9672663	SRR13240784	Illumina NovaSeq 6000	SampleB_IDT1_ST06_100ng_LIB4	PAIRED	fastq		6.24 GB	SampleB_IDT1_ST06_100ng_LIB4.R1.fastq.gz	SampleB_IDT1_ST06_100ng_LIB4.R2.fastq.gz						
Sample C	SAMN16786362	IDT1	SRX9672614	SRR13240833	Illumina NovaSeq 6000	SampleC_IDT1_ST04_100ng_LIB1	PAIRED	fastq		6.06 GB	SampleC_IDT1_ST04_100ng_LIB1.R1.fastq.gz	SampleC_IDT1_ST04_100ng_LIB1.R2.fastq.gz						
Sample C	SAMN16786362	IDT1	SRX9672615	SRR13240832	Illumina NovaSeq 6000	SampleC_IDT1_ST04_100ng_LIB2	PAIRED	fastq		6.13 GB	SampleC_IDT1_ST04_100ng_LIB2.R1.fastq.gz	SampleC_IDT1_ST04_100ng_LIB2.R2.fastq.gz						
Sample C	SAMN16786362	IDT1	SRX9672616	SRR13240831	Illumina NovaSeq 6000	SampleC_IDT1_ST04_100ng_LIB3	PAIRED	fastq		6.07 GB	SampleC_IDT1_ST04_100ng_LIB3.R1.fastq.gz	SampleC_IDT1_ST04_100ng_LIB3.R2.fastq.gz						
Sample C	SAMN16786362	IDT1	SRX9672617	SRR13240830	Illumina NovaSeq 6000	SampleC_IDT1_ST04_100ng_LIB4	PAIRED	fastq		6.09 GB	SampleC_IDT1_ST04_100ng_LIB4.R1.fastq.gz	SampleC_IDT1_ST04_100ng_LIB4.R2.fastq.gz						
Sample C	SAMN16786362	IDT1	SRX9672639	SRR13240808	Illumina NovaSeq 6000	SampleC_IDT1_ST05_100ng_LIB1	PAIRED	fastq		6.10 GB	SampleC_IDT1_ST05_100ng_LIB1.R1.fastq.gz	SampleC_IDT1_ST05_100ng_LIB1.R2.fastq.gz						
Sample C	SAMN16786362	IDT1	SRX9672638	SRR13240809	Illumina NovaSeq 6000	SampleC_IDT1_ST05_100ng_LIB2	PAIRED	fastq		6.11 GB	SampleC_IDT1_ST05_100ng_LIB2.R1.fastq.gz	SampleC_IDT1_ST05_100ng_LIB2.R2.fastq.gz						
Sample C	SAMN16786362	IDT1	SRX9672637	SRR13240810	Illumina NovaSeq 6000	SampleC_IDT1_ST05_100ng_LIB3	PAIRED	fastq		6.16 GB	SampleC_IDT1_ST05_100ng_LIB3.R1.fastq.gz	SampleC_IDT1_ST05_100ng_LIB3.R2.fastq.gz						
Sample C	SAMN16786362	IDT1	SRX9672636	SRR13240811	Illumina NovaSeq 6000	SampleC_IDT1_ST05_100ng_LIB4	PAIRED	fastq		6.15 GB	SampleC_IDT1_ST05_100ng_LIB4.R1.fastq.gz	SampleC_IDT1_ST05_100ng_LIB4.R2.fastq.gz						
Sample C	SAMN16786362	IDT1	SRX9672662	SRR13240785	Illumina NovaSeq 6000	SampleC_IDT1_ST06_100ng_LIB1	PAIRED	fastq		6.26 GB	SampleC_IDT1_ST06_100ng_LIB1.R1.fastq.gz	SampleC_IDT1_ST06_100ng_LIB1.R2.fastq.gz						
Sample C	SAMN16786362	IDT1	SRX9672661	SRR13240786	Illumina NovaSeq 6000	SampleC_IDT1_ST06_100ng_LIB2	PAIRED	fastq		6.16 GB	SampleC_IDT1_ST06_100ng_LIB2.R1.fastq.gz	SampleC_IDT1_ST06_100ng_LIB2.R2.fastq.gz						
Sample C	SAMN16786362	IDT1	SRX9672660	SRR13240787	Illumina NovaSeq 6000	SampleC_IDT1_ST06_100ng_LIB3	PAIRED	fastq		6.17 GB	SampleC_IDT1_ST06_100ng_LIB3.R1.fastq.gz	SampleC_IDT1_ST06_100ng_LIB3.R2.fastq.gz						
Sample C	SAMN16786362	IDT1	SRX9672659	SRR13240788	Illumina NovaSeq 6000	SampleC_IDT1_ST06_100ng_LIB4	PAIRED	fastq		6.16 GB	SampleC_IDT1_ST06_100ng_LIB4.R1.fastq.gz	SampleC_IDT1_ST06_100ng_LIB4.R2.fastq.gz						
Sample A	SAMN16786360	IGT1	SRX9637187	SRR13203938	Illumina HiSeq 2500	SampleA_IGT1_ST07_100ng_LIB1	PAIRED	fastq		4.24 GB	SampleA_IGT1_ST07_100ng_LIB1.R1.fastq.gz	SampleA_IGT1_ST07_100ng_LIB1.R2.fastq.gz						
Sample A	SAMN16786360	IGT1	SRX9637188	SRR13203937	Illumina HiSeq 2500	SampleA_IGT1_ST07_100ng_LIB2	PAIRED	fastq		4.17 GB	SampleA_IGT1_ST07_100ng_LIB2.R1.fastq.gz	SampleA_IGT1_ST07_100ng_LIB2.R2.fastq.gz						
Sample A	SAMN16786360	IGT1	SRX9637199	SRR13203925	Illumina HiSeq 2500	SampleA_IGT1_ST07_100ng_LIB3	PAIRED	fastq		4.41 GB	SampleA_IGT1_ST07_100ng_LIB3.R1.fastq.gz	SampleA_IGT1_ST07_100ng_LIB3.R2.fastq.gz						
Sample A	SAMN16786360	IGT1	SRX9637210	SRR13203914	Illumina HiSeq 2500	SampleA_IGT1_ST07_100ng_LIB4	PAIRED	fastq		4.25 GB	SampleA_IGT1_ST07_100ng_LIB4.R1.fastq.gz	SampleA_IGT1_ST07_100ng_LIB4.R2.fastq.gz						
Sample A	SAMN16786360	IGT1	SRX9637200	SRR13203924	Illumina HiSeq 2500	SampleA_IGT1_ST08_100ng_LIB1	PAIRED	fastq		4.56 GB	SampleA_IGT1_ST08_100ng_LIB1.R1.fastq.gz	SampleA_IGT1_ST08_100ng_LIB1.R2.fastq.gz						
Sample A	SAMN16786360	IGT1	SRX9637201	SRR13203923	Illumina HiSeq 2500	SampleA_IGT1_ST08_100ng_LIB2	PAIRED	fastq		4.71 GB	SampleA_IGT1_ST08_100ng_LIB2.R1.fastq.gz	SampleA_IGT1_ST08_100ng_LIB2.R2.fastq.gz						
Sample A	SAMN16786360	IGT1	SRX9637202	SRR13203922	Illumina HiSeq 2500	SampleA_IGT1_ST08_100ng_LIB3	PAIRED	fastq		4.55 GB	SampleA_IGT1_ST08_100ng_LIB3.R1.fastq.gz	SampleA_IGT1_ST08_100ng_LIB3.R2.fastq.gz						
Sample A	SAMN16786360	IGT1	SRX9637203	SRR13203921	Illumina HiSeq 2500	SampleA_IGT1_ST08_100ng_LIB4	PAIRED	fastq		4.38 GB	SampleA_IGT1_ST08_100ng_LIB4.R1.fastq.gz	SampleA_IGT1_ST08_100ng_LIB4.R2.fastq.gz						
Sample A	SAMN16786360	IGT1	SRX9637162	SRR13203962	Illumina HiSeq 2500	SampleA_IGT1_ST09_100ng_LIB1	PAIRED	fastq		6.95 GB	SampleA_IGT1_ST09_100ng_LIB1.R1.fastq.gz	SampleA_IGT1_ST09_100ng_LIB1.R2.fastq.gz						
Sample A	SAMN16786360	IGT1	SRX9637163	SRR13203961	Illumina HiSeq 2500	SampleA_IGT1_ST09_100ng_LIB2	PAIRED	fastq		5.15 GB	SampleA_IGT1_ST09_100ng_LIB2.R1.fastq.gz	SampleA_IGT1_ST09_100ng_LIB2.R2.fastq.gz						
Sample A	SAMN16786360	IGT1	SRX9637164	SRR13203960	Illumina HiSeq 2500	SampleA_IGT1_ST09_100ng_LIB3	PAIRED	fastq		5.33 GB	SampleA_IGT1_ST09_100ng_LIB3.R1.fastq.gz	SampleA_IGT1_ST09_100ng_LIB3.R2.fastq.gz						
Sample A	SAMN16786360	IGT1	SRX9637165	SRR13203959	Illumina HiSeq 2500	SampleA_IGT1_ST09_100ng_LIB4	PAIRED	fastq		5.17 GB	SampleA_IGT1_ST09_100ng_LIB4.R1.fastq.gz	SampleA_IGT1_ST09_100ng_LIB4.R2.fastq.gz						
Sample Spike-in	SAMN16786363	IGT1	SRX9637185	SRR13203939	Illumina HiSeq 2500	SampleAC5_IGT1_ST07_100ng_LIB1	PAIRED	fastq		4.18 GB	SampleAC5_IGT1_ST07_100ng_LIB1.R1.fastq.gz	SampleAC5_IGT1_ST07_100ng_LIB1.R2.fastq.gz						
Sample Spike-in	SAMN16786363	IGT1	SRX9637186	SRR13203934	Illumina HiSeq 2500	SampleAC5_IGT1_ST07_100ng_LIB2	PAIRED	fastq		3.77 GB	SampleAC5_IGT1_ST07_100ng_LIB2.R1.fastq.gz	SampleAC5_IGT1_ST07_100ng_LIB2.R2.fastq.gz						
Sample Spike-in	SAMN16786363	IGT1	SRX9637189	SRR13203936	Illumina HiSeq 2500	SampleAC5_IGT1_ST07_100ng_LIB3	PAIRED	fastq		3.90 GB	SampleAC5_IGT1_ST07_100ng_LIB3.R1.fastq.gz	SampleAC5_IGT1_ST07_100ng_LIB3.R2.fastq.gz						
Sample Spike-in	SAMN16786363	IGT1	SRX9637190	SRR13203935	Illumina HiSeq 2500	SampleAC5_IGT1_ST07_100ng_LIB4	PAIRED	fastq		3.78 GB	SampleAC5_IGT1_ST07_100ng_LIB4.R1.fastq.gz	SampleAC5_IGT1_ST07_100ng_LIB4.R2.fastq.gz						
Sample Spike-in	SAMN16786363	IGT1	SRX9637208	SRR13203916	Illumina HiSeq 2500	SampleAC5_IGT1_ST08_100ng_LIB1	PAIRED	fastq		3.43 GB	SampleAC5_IGT1_ST08_100ng_LIB1.R1.fastq.gz	SampleAC5_IGT1_ST08_100ng_LIB1.R2.fastq.gz						
Sample Spike-in	SAMN16786363	IGT1	SRX9637209	SRR13203915	Illumina HiSeq 2500	SampleAC5_IGT1_ST08_100ng_LIB2	PAIRED	fastq		4.13 GB	SampleAC5_IGT1_ST08_100ng_LIB2.R1.fastq.gz	SampleAC5_IGT1_ST08_100ng_LIB2.R2.fastq.gz						
Sample Spike-in	SAMN16786363	IGT1	SRX9637211	SRR13203913	Illumina HiSeq 2500	SampleAC5_IGT1_ST08_100ng_LIB3	PAIRED	fastq		3.44 GB	SampleAC5_IGT1_ST08_100ng_LIB3.R1.fastq.gz	SampleAC5_IGT1_ST08_100ng_LIB3.R2.fastq.gz						
Sample Spike-in	SAMN16786363	IGT1	SRX9637212	SRR13203912	Illumina HiSeq 2500	SampleAC5_IGT1_ST08_100ng_LIB4	PAIRED	fastq		3.79 GB	SampleAC5_IGT1_ST08_100ng_LIB4.R1.fastq.gz	SampleAC5_IGT1_ST08_100ng_LIB4.R2.fastq.gz						
Sample Spike-in	SAMN16786363	IGT1	SRX9637170	SRR13203954	Illumina HiSeq 2500	SampleAC5_IGT1_ST09_100ng_LIB1	PAIRED	fastq		8.44 GB	SampleAC5_IGT1_ST09_100ng_LIB1.R1.fastq.gz	SampleAC5_IGT1_ST09_100ng_LIB1.R2.fastq.gz						
Sample Spike-in	SAMN16786363	IGT1	SRX9637171	SRR13203953	Illumina HiSeq 2500	SampleAC5_IGT1_ST09_100ng_LIB2	PAIRED	fastq		4.55 GB	SampleAC5_IGT1_ST09_100ng_LIB2.R1.fastq.gz	SampleAC5_IGT1_ST09_100ng_LIB2.R2.fastq.gz						
Sample Spike-in	SAMN16786363	IGT1	SRX9637173	SRR13203951	Illumina HiSeq 2500	SampleAC5_IGT1_ST09_100ng_LIB3	PAIRED	fastq		4.51 GB	SampleAC5_IGT1_ST09_100ng_LIB3.R1.fastq.gz	SampleAC5_IGT1_ST09_100ng_LIB3.R2.fastq.gz						
Sample Spike-in	SAMN16786363	IGT1	SRX9637174	SRR13203950	Illumina HiSeq 2500	SampleAC5_IGT1_ST09_100ng_LIB4	PAIRED	fastq		4.24 GB	SampleAC5_IGT1_ST09_100ng_LIB4.R1.fastq.gz	SampleAC5_IGT1_ST09_100ng_LIB4.R2.fastq.gz						
Sample B	SAMN16786361	IGT1	SRX9637191	SRR13203933	Illumina HiSeq 2500	SampleB_IGT1_ST07_100ng_LIB1	PAIRED	fastq		4.45 GB	SampleB_IGT1_ST07_100ng_LIB1.R1.fastq.gz	SampleB_IGT1_ST07_100ng_LIB1.R2.fastq.gz						
Sample B	SAMN16786361	IGT1	SRX9637192	SRR13203932	Illumina HiSeq 2500	SampleB_IGT1_ST07_100ng_LIB2	PAIRED	fastq		4.28 GB	SampleB_IGT1_ST07_100ng_LIB2.R1.fastq.gz	SampleB_IGT1_ST07_100ng_LIB2.R2.fastq.gz						
Sample B	SAMN16786361	IGT1	SRX9637193	SRR13203931	Illumina HiSeq 2500	SampleB_IGT1_ST07_100ng_LIB3	PAIRED	fastq		4.61 GB	SampleB_IGT1_ST07_100ng_LIB3.R1.fastq.gz	SampleB_IGT1_ST07_100ng_LIB3.R2.fastq.gz						
Sample B	SAMN16786361	IGT1	SRX9637194	SRR13203930	Illumina HiSeq 2500	SampleB_IGT1_ST07_100ng_LIB4	PAIRED	fastq		4.22 GB	SampleB_IGT1_ST07_100ng_LIB4.R1.fastq.gz	SampleB_IGT1_ST07_100ng_LIB4.R2.fastq.gz						
Sample B	SAMN16786361	IGT1	SRX9637213	SRR13203911	Illumina HiSeq 2500	SampleB_IGT1_ST08_100ng_LIB1	PAIRED	fastq		4.73 GB	SampleB_IGT1_ST08_100ng_LIB1.R1.fastq.gz	SampleB_IGT1_ST08_100ng_LIB1.R2.fastq.gz						
Sample B	SAMN16786361	IGT1	SRX9637214	SRR13203910	Illumina HiSeq 2500	SampleB_IGT1_ST08_100ng_LIB2	PAIRED	fastq		4.38 GB	SampleB_IGT1_ST08_100ng_LIB2.R1.fastq.gz	SampleB_IGT1_ST08_100ng_LIB2.R2.fastq.gz						
Sample B	SAMN16786361	IGT1	SRX9637215	SRR13203909	Illumina HiSeq 2500	SampleB_IGT1_ST08_100ng_LIB3	PAIRED	fastq		4.07 GB	SampleB_IGT1_ST08_100ng_LIB3.R1.fastq.gz	SampleB_IGT1_ST08_100ng_LIB3.R2.fastq.gz						
Sample B	SAMN16786361	IGT1	SRX9637216	SRR13203908	Illumina HiSeq 2500	SampleB_IGT1_ST08_100ng_LIB4	PAIRED	fastq		4.46 GB	SampleB_IGT1_ST08_100ng_LIB4.R1.fastq.gz	SampleB_IGT1_ST08_100ng_LIB4.R2.fastq.gz						
Sample B	SAMN16786361	IGT1	SRX9637175	SRR13203949	Illumina HiSeq 2500	SampleB_IGT1_ST09_100ng_LIB1	PAIRED	fastq		8.22 GB	SampleB_IGT1_ST09_100ng_LIB1.R1.fastq.gz	SampleB_IGT1_ST09_100ng_LIB1.R2.fastq.gz						
Sample B	SAMN16786361	IGT1	SRX9637176	SRR13203948	Illumina HiSeq 2500	SampleB_IGT1_ST09_100ng_LIB2	PAIRED	fastq		5.78 GB	SampleB_IGT1_ST09_100ng_LIB2.R1.fastq.gz	SampleB_IGT1_ST09_100ng_LIB2.R2.fastq.gz						
Sample B	SAMN16786361	IGT1	SRX9637177	SRR13203947	Illumina HiSeq 2500	SampleB_IGT1_ST09_100ng_LIB3	PAIRED	fastq		5.39 GB	SampleB_IGT1_ST09_100ng_LIB3.R1.fastq.gz	SampleB_IGT1_ST09_100ng_LIB3.R2.fastq.gz						
Sample B	SAMN16786361	IGT1	SRX9637178	SRR13203946	Illumina HiSeq 2500	SampleB_IGT1_ST09_100ng_LIB4	PAIRED	fastq		5.06 GB	SampleB_IGT1_ST09_100ng_LIB4.R1.fastq.gz	SampleB_IGT1_ST09_100ng_LIB4.R2.fastq.gz						
Sample C	SAMN16786362	IGT1	SRX9637195	SRR13203929	Illumina HiSeq 2500	SampleC_IGT1_ST07_100ng_LIB1	PAIRED	fastq		8.12 GB	SampleC_IGT1_ST07_100ng_LIB1.R1.fastq.gz	SampleC_IGT1_ST07_100ng_LIB1.R2.fastq.gz						
Sample C	SAMN16786362	IGT1	SRX9637196	SRR13203928	Illumina HiSeq 2500	SampleC_IGT1_ST07_100ng_LIB2	PAIRED	fastq		8.17 GB	SampleC_IGT1_ST07_100ng_LIB2.R1.fastq.gz	SampleC_IGT1_ST07_100ng_LIB2.R2.fastq.gz						
Sample C	SAMN16786362	IGT1	SRX9637197	SRR13203927	Illumina HiSeq 2500	SampleC_IGT1_ST07_100ng_LIB3	PAIRED	fastq		8.18 GB	SampleC_IGT1_ST07_100ng_LIB3.R1.fastq.gz	SampleC_IGT1_ST07_100ng_LIB3.R2.fastq.gz						
Sample C	SAMN16786362	IGT1	SRX9637198	SRR13203926	Illumina HiSeq 2500	SampleC_IGT1_ST07_100ng_LIB4	PAIRED	fastq		8.25 GB	SampleC_IGT1_ST07_100ng_LIB4.R1.fastq.gz	SampleC_IGT1_ST07_100ng_LIB4.R2.fastq.gz						
Sample C	SAMN16786362	IGT1	SRX9637217	SRR13203907	Illumina HiSeq 2500	SampleC_IGT1_ST08_100ng_LIB1	PAIRED	fastq		8.04 GB	SampleC_IGT1_ST08_100ng_LIB1.R1.fastq.gz	SampleC_IGT1_ST08_100ng_LIB1.R2.fastq.gz						
Sample C	SAMN16786362	IGT1	SRX9637218	SRR13203906	Illumina HiSeq 2500	SampleC_IGT1_ST08_100ng_LIB2	PAIRED	fastq		7.56 GB	SampleC_IGT1_ST08_100ng_LIB2.R1.fastq.gz	SampleC_IGT1_ST08_100ng_LIB2.R2.fastq.gz						
Sample C	SAMN16786362	IGT1	SRX9637159	SRR13203965	Illumina HiSeq 2500	SampleC_IGT1_ST08_100ng_LIB3	PAIRED	fastq		7.38 GB	SampleC_IGT1_ST08_100ng_LIB3.R1.fastq.gz	SampleC_IGT1_ST08_100ng_LIB3.R2.fastq.gz						
Sample C	SAMN16786362	IGT1	SRX9637160	SRR13203964	Illumina HiSeq 2500	SampleC_IGT1_ST08_100ng_LIB4	PAIRED	fastq		7.47 GB	SampleC_IGT1_ST08_100ng_LIB4.R1.fastq.gz	SampleC_IGT1_ST08_100ng_LIB4.R2.fastq.gz						
Sample C	SAMN16786362	IGT1	SRX9637179	SRR13203945	Illumina HiSeq 2500	SampleC_IGT1_ST09_100ng_LIB1	PAIRED	fastq		15.25 GB	SampleC_IGT1_ST09_100ng_LIB1.R1.fastq.gz	SampleC_IGT1_ST09_100ng_LIB1.R2.fastq.gz						
Sample C	SAMN16786362	IGT1	SRX9637180	SRR13203944	Illumina HiSeq 2500	SampleC_IGT1_ST09_100ng_LIB2	PAIRED	fastq		10.43 GB	SampleC_IGT1_ST09_100ng_LIB2.R1.fastq.gz	SampleC_IGT1_ST09_100ng_LIB2.R2.fastq.gz						
Sample C	SAMN16786362	IGT1	SRX9637181	SRR13203943	Illumina HiSeq 2500	SampleC_IGT1_ST09_100ng_LIB3	PAIRED	fastq		10.42 GB	SampleC_IGT1_ST09_100ng_LIB3.R1.fastq.gz	SampleC_IGT1_ST09_100ng_LIB3.R2.fastq.gz						
Sample C	SAMN16786362	IGT1	SRX9637182	SRR13203942	Illumina HiSeq 2500	SampleC_IGT1_ST09_100ng_LIB4	PAIRED	fastq		9.77 GB	SampleC_IGT1_ST09_100ng_LIB4.R1.fastq.gz	SampleC_IGT1_ST09_100ng_LIB4.R2.fastq.gz						
Sample A	SAMN16786360	ILM1	SRX9638519	SRR13205294	NextSeq 550	SampleA_ILM1_ST10_50ng_LIB1	PAIRED	fastq		8.29 GB	SampleA_ILM1_ST10_50ng_LIB1_PT1.R1.fastq.gz	SampleA_ILM1_ST10_50ng_LIB1_PT1.R2.fastq.gz	SampleA_ILM1_ST10_50ng_LIB1_PT2.R1.fastq.gz	SampleA_ILM1_ST10_50ng_LIB1_PT2.R2.fastq.gz	SampleA_ILM1_ST10_50ng_LIB1_PT3.R1.fastq.gz	SampleA_ILM1_ST10_50ng_LIB1_PT3.R2.fastq.gz	SampleA_ILM1_ST10_50ng_LIB1_PT4.R1.fastq.gz	SampleA_ILM1_ST10_50ng_LIB1_PT4.R2.fastq.gz
Sample A	SAMN16786360	ILM1	SRX9638520	SRR13205293	NextSeq 550	SampleA_ILM1_ST10_50ng_LIB2	PAIRED	fastq		7.68 GB	SampleA_ILM1_ST10_50ng_LIB2_PT1.R1.fastq.gz	SampleA_ILM1_ST10_50ng_LIB2_PT1.R2.fastq.gz	SampleA_ILM1_ST10_50ng_LIB2_PT2.R1.fastq.gz	SampleA_ILM1_ST10_50ng_LIB2_PT2.R2.fastq.gz	SampleA_ILM1_ST10_50ng_LIB2_PT3.R1.fastq.gz	SampleA_ILM1_ST10_50ng_LIB2_PT3.R2.fastq.gz	SampleA_ILM1_ST10_50ng_LIB2_PT4.R1.fastq.gz	SampleA_ILM1_ST10_50ng_LIB2_PT4.R2.fastq.gz
Sample A	SAMN16786360	ILM1	SRX9638531	SRR13205282	NextSeq 550	SampleA_ILM1_ST10_50ng_LIB3	PAIRED	fastq		7.96 GB	SampleA_ILM1_ST10_50ng_LIB3_PT1.R1.fastq.gz	SampleA_ILM1_ST10_50ng_LIB3_PT1.R2.fastq.gz	SampleA_ILM1_ST10_50ng_LIB3_PT2.R1.fastq.gz	SampleA_ILM1_ST10_50ng_LIB3_PT2.R2.fastq.gz	SampleA_ILM1_ST10_50ng_LIB3_PT3.R1.fastq.gz	SampleA_ILM1_ST10_50ng_LIB3_PT3.R2.fastq.gz	SampleA_ILM1_ST10_50ng_LIB3_PT4.R1.fastq.gz	SampleA_ILM1_ST10_50ng_LIB3_PT4.R2.fastq.gz
Sample A	SAMN16786360	ILM1	SRX9638532	SRR13205281	NextSeq 550	SampleA_ILM1_ST10_50ng_LIB4	PAIRED	fastq		8.55 GB	SampleA_ILM1_ST10_50ng_LIB4_PT1.R1.fastq.gz	SampleA_ILM1_ST10_50ng_LIB4_PT1.R2.fastq.gz	SampleA_ILM1_ST10_50ng_LIB4_PT2.R1.fastq.gz	SampleA_ILM1_ST10_50ng_LIB4_PT2.R2.fastq.gz	SampleA_ILM1_ST10_50ng_LIB4_PT3.R1.fastq.gz	SampleA_ILM1_ST10_50ng_LIB4_PT3.R2.fastq.gz	SampleA_ILM1_ST10_50ng_LIB4_PT4.R1.fastq.gz	SampleA_ILM1_ST10_50ng_LIB4_PT4.R2.fastq.gz
Sample A	SAMN16786360	ILM1	SRX9719039	SRR13289949	NextSeq 550	SampleA_ILM1_ST12_50ng_LIB1	PAIRED	fastq		6.55 GB	SampleA_ILM1_ST12_50ng_LIB1_PT1.R1.fastq.gz	SampleA_ILM1_ST12_50ng_LIB1_PT1.R2.fastq.gz	SampleA_ILM1_ST12_50ng_LIB1_PT2.R1.fastq.gz	SampleA_ILM1_ST12_50ng_LIB1_PT2.R2.fastq.gz	SampleA_ILM1_ST12_50ng_LIB1_PT3.R1.fastq.gz	SampleA_ILM1_ST12_50ng_LIB1_PT3.R2.fastq.gz	SampleA_ILM1_ST12_50ng_LIB1_PT4.R1.fastq.gz	SampleA_ILM1_ST12_50ng_LIB1_PT4.R2.fastq.gz
Sample A	SAMN16786360	ILM1	SRX9719040	SRR13289948	NextSeq 550	SampleA_ILM1_ST12_50ng_LIB2	PAIRED	fastq		6.66 GB	SampleA_ILM1_ST12_50ng_LIB2_PT1.R1.fastq.gz	SampleA_ILM1_ST12_50ng_LIB2_PT1.R2.fastq.gz	SampleA_ILM1_ST12_50ng_LIB2_PT2.R1.fastq.gz	SampleA_ILM1_ST12_50ng_LIB2_PT2.R2.fastq.gz	SampleA_ILM1_ST12_50ng_LIB2_PT3.R1.fastq.gz	SampleA_ILM1_ST12_50ng_LIB2_PT3.R2.fastq.gz	SampleA_ILM1_ST12_50ng_LIB2_PT4.R1.fastq.gz	SampleA_ILM1_ST12_50ng_LIB2_PT4.R2.fastq.gz
Sample A	SAMN16786360	ILM1	SRX9719041	SRR13289947	NextSeq 550	SampleA_ILM1_ST23_50ng_LIB1	PAIRED	fastq		6.71 GB	SampleA_ILM1_ST23_50ng_LIB1_PT1.R1.fastq.gz	SampleA_ILM1_ST23_50ng_LIB1_PT1.R2.fastq.gz	SampleA_ILM1_ST23_50ng_LIB1_PT2.R1.fastq.gz	SampleA_ILM1_ST23_50ng_LIB1_PT2.R2.fastq.gz	SampleA_ILM1_ST23_50ng_LIB1_PT3.R1.fastq.gz	SampleA_ILM1_ST23_50ng_LIB1_PT3.R2.fastq.gz	SampleA_ILM1_ST23_50ng_LIB1_PT4.R1.fastq.gz	SampleA_ILM1_ST23_50ng_LIB1_PT4.R2.fastq.gz
Sample A	SAMN16786360	ILM1	SRX9719042	SRR13289946	NextSeq 550	SampleA_ILM1_ST23_50ng_LIB2	PAIRED	fastq		5.23 GB	SampleA_ILM1_ST23_50ng_LIB2_PT1.R1.fastq.gz	SampleA_ILM1_ST23_50ng_LIB2_PT1.R2.fastq.gz	SampleA_ILM1_ST23_50ng_LIB2_PT2.R1.fastq.gz	SampleA_ILM1_ST23_50ng_LIB2_PT2.R2.fastq.gz	SampleA_ILM1_ST23_50ng_LIB2_PT3.R1.fastq.gz	SampleA_ILM1_ST23_50ng_LIB2_PT3.R2.fastq.gz	SampleA_ILM1_ST23_50ng_LIB2_PT4.R1.fastq.gz	SampleA_ILM1_ST23_50ng_LIB2_PT4.R2.fastq.gz
Sample A	SAMN16786360	ILM1	SRX9719026	SRR13289962	NextSeq 550	SampleA_ILM1_ST23_50ng_LIB3	PAIRED	fastq		6.91 GB	SampleA_ILM1_ST23_50ng_LIB3_PT1.R1.fastq.gz	SampleA_ILM1_ST23_50ng_LIB3_PT1.R2.fastq.gz	SampleA_ILM1_ST23_50ng_LIB3_PT2.R1.fastq.gz	SampleA_ILM1_ST23_50ng_LIB3_PT2.R2.fastq.gz	SampleA_ILM1_ST23_50ng_LIB3_PT3.R1.fastq.gz	SampleA_ILM1_ST23_50ng_LIB3_PT3.R2.fastq.gz	SampleA_ILM1_ST23_50ng_LIB3_PT4.R1.fastq.gz	SampleA_ILM1_ST23_50ng_LIB3_PT4.R2.fastq.gz
Sample A	SAMN16786360	ILM1	SRX9719025	SRR13289963	NextSeq 550	SampleA_ILM1_ST23_50ng_LIB4	PAIRED	fastq		0.19 GB	SampleA_ILM1_ST23_50ng_LIB4_PT1.R1.fastq.gz	SampleA_ILM1_ST23_50ng_LIB4_PT1.R2.fastq.gz	SampleA_ILM1_ST23_50ng_LIB4_PT2.R1.fastq.gz	SampleA_ILM1_ST23_50ng_LIB4_PT2.R2.fastq.gz	SampleA_ILM1_ST23_50ng_LIB4_PT3.R1.fastq.gz	SampleA_ILM1_ST23_50ng_LIB4_PT3.R2.fastq.gz	SampleA_ILM1_ST23_50ng_LIB4_PT4.R1.fastq.gz	SampleA_ILM1_ST23_50ng_LIB4_PT4.R2.fastq.gz
Sample A	SAMN16786360	ILM1	SRX9719044	SRR13289944	NextSeq 550	SampleA_ILM1_ST29_50ng_LIB3	PAIRED	fastq		5.87 GB	SampleA_ILM1_ST29_50ng_LIB3_PT1.R1.fastq.gz	SampleA_ILM1_ST29_50ng_LIB3_PT1.R2.fastq.gz	SampleA_ILM1_ST29_50ng_LIB3_PT2.R1.fastq.gz	SampleA_ILM1_ST29_50ng_LIB3_PT2.R2.fastq.gz	SampleA_ILM1_ST29_50ng_LIB3_PT3.R1.fastq.gz	SampleA_ILM1_ST29_50ng_LIB3_PT3.R2.fastq.gz	SampleA_ILM1_ST29_50ng_LIB3_PT4.R1.fastq.gz	SampleA_ILM1_ST29_50ng_LIB3_PT4.R2.fastq.gz
Sample Spike-in	SAMN16786363	ILM1	SRX9638537	SRR13205276	NextSeq 550	SampleAC5_ILM1_ST10_50ng_LIB1	PAIRED	fastq		9.39 GB	SampleAC5_ILM1_ST10_50ng_LIB1_PT1.R1.fastq.gz	SampleAC5_ILM1_ST10_50ng_LIB1_PT1.R2.fastq.gz	SampleAC5_ILM1_ST10_50ng_LIB1_PT2.R1.fastq.gz	SampleAC5_ILM1_ST10_50ng_LIB1_PT2.R2.fastq.gz	SampleAC5_ILM1_ST10_50ng_LIB1_PT3.R1.fastq.gz	SampleAC5_ILM1_ST10_50ng_LIB1_PT3.R2.fastq.gz	SampleAC5_ILM1_ST10_50ng_LIB1_PT4.R1.fastq.gz	SampleAC5_ILM1_ST10_50ng_LIB1_PT4.R2.fastq.gz
Sample Spike-in	SAMN16786363	ILM1	SRX9638518	SRR13205295	NextSeq 550	SampleAC5_ILM1_ST10_50ng_LIB2	PAIRED	fastq		8.46 GB	SampleAC5_ILM1_ST10_50ng_LIB2_PT1.R1.fastq.gz	SampleAC5_ILM1_ST10_50ng_LIB2_PT1.R2.fastq.gz	SampleAC5_ILM1_ST10_50ng_LIB2_PT2.R1.fastq.gz	SampleAC5_ILM1_ST10_50ng_LIB2_PT2.R2.fastq.gz	SampleAC5_ILM1_ST10_50ng_LIB2_PT3.R1.fastq.gz	SampleAC5_ILM1_ST10_50ng_LIB2_PT3.R2.fastq.gz	SampleAC5_ILM1_ST10_50ng_LIB2_PT4.R1.fastq.gz	SampleAC5_ILM1_ST10_50ng_LIB2_PT4.R2.fastq.gz
Sample Spike-in	SAMN16786363	ILM1	SRX9638521	SRR13205292	NextSeq 550	SampleAC5_ILM1_ST10_50ng_LIB3	PAIRED	fastq		9.75 GB	SampleAC5_ILM1_ST10_50ng_LIB3_PT1.R1.fastq.gz	SampleAC5_ILM1_ST10_50ng_LIB3_PT1.R2.fastq.gz	SampleAC5_ILM1_ST10_50ng_LIB3_PT2.R1.fastq.gz	SampleAC5_ILM1_ST10_50ng_LIB3_PT2.R2.fastq.gz	SampleAC5_ILM1_ST10_50ng_LIB3_PT3.R1.fastq.gz	SampleAC5_ILM1_ST10_50ng_LIB3_PT3.R2.fastq.gz	SampleAC5_ILM1_ST10_50ng_LIB3_PT4.R1.fastq.gz	SampleAC5_ILM1_ST10_50ng_LIB3_PT4.R2.fastq.gz
Sample Spike-in	SAMN16786363	ILM1	SRX9638522	SRR13205291	NextSeq 550	SampleAC5_ILM1_ST10_50ng_LIB4	PAIRED	fastq		8.74 GB	SampleAC5_ILM1_ST10_50ng_LIB4_PT1.R1.fastq.gz	SampleAC5_ILM1_ST10_50ng_LIB4_PT1.R2.fastq.gz	SampleAC5_ILM1_ST10_50ng_LIB4_PT2.R1.fastq.gz	SampleAC5_ILM1_ST10_50ng_LIB4_PT2.R2.fastq.gz	SampleAC5_ILM1_ST10_50ng_LIB4_PT3.R1.fastq.gz	SampleAC5_ILM1_ST10_50ng_LIB4_PT3.R2.fastq.gz	SampleAC5_ILM1_ST10_50ng_LIB4_PT4.R1.fastq.gz	SampleAC5_ILM1_ST10_50ng_LIB4_PT4.R2.fastq.gz
Sample Spike-in	SAMN16786363	ILM1	SRX9719049	SRR13289939	NextSeq 550	SampleAC5_ILM1_ST12_50ng_LIB1	PAIRED	fastq		6.78 GB	SampleAC5_ILM1_ST12_50ng_LIB1_PT1.R1.fastq.gz	SampleAC5_ILM1_ST12_50ng_LIB1_PT1.R2.fastq.gz	SampleAC5_ILM1_ST12_50ng_LIB1_PT2.R1.fastq.gz	SampleAC5_ILM1_ST12_50ng_LIB1_PT2.R2.fastq.gz	SampleAC5_ILM1_ST12_50ng_LIB1_PT3.R1.fastq.gz	SampleAC5_ILM1_ST12_50ng_LIB1_PT3.R2.fastq.gz	SampleAC5_ILM1_ST12_50ng_LIB1_PT4.R1.fastq.gz	SampleAC5_ILM1_ST12_50ng_LIB1_PT4.R2.fastq.gz
Sample Spike-in	SAMN16786363	ILM1	SRX9719050	SRR13289938	NextSeq 550	SampleAC5_ILM1_ST12_50ng_LIB2	PAIRED	fastq		6.67 GB	SampleAC5_ILM1_ST12_50ng_LIB2_PT1.R1.fastq.gz	SampleAC5_ILM1_ST12_50ng_LIB2_PT1.R2.fastq.gz	SampleAC5_ILM1_ST12_50ng_LIB2_PT2.R1.fastq.gz	SampleAC5_ILM1_ST12_50ng_LIB2_PT2.R2.fastq.gz	SampleAC5_ILM1_ST12_50ng_LIB2_PT3.R1.fastq.gz	SampleAC5_ILM1_ST12_50ng_LIB2_PT3.R2.fastq.gz	SampleAC5_ILM1_ST12_50ng_LIB2_PT4.R1.fastq.gz	SampleAC5_ILM1_ST12_50ng_LIB2_PT4.R2.fastq.gz
Sample Spike-in	SAMN16786363	ILM1	SRX9719057	SRR13289931	NextSeq 550	SampleAC5_ILM1_ST23_50ng_LIB1	PAIRED	fastq		8.03 GB	SampleAC5_ILM1_ST23_50ng_LIB1_PT1.R1.fastq.gz	SampleAC5_ILM1_ST23_50ng_LIB1_PT1.R2.fastq.gz	SampleAC5_ILM1_ST23_50ng_LIB1_PT2.R1.fastq.gz	SampleAC5_ILM1_ST23_50ng_LIB1_PT2.R2.fastq.gz	SampleAC5_ILM1_ST23_50ng_LIB1_PT3.R1.fastq.gz	SampleAC5_ILM1_ST23_50ng_LIB1_PT3.R2.fastq.gz	SampleAC5_ILM1_ST23_50ng_LIB1_PT4.R1.fastq.gz	SampleAC5_ILM1_ST23_50ng_LIB1_PT4.R2.fastq.gz
Sample Spike-in	SAMN16786363	ILM1	SRX9719056	SRR13289932	NextSeq 550	SampleAC5_ILM1_ST23_50ng_LIB2	PAIRED	fastq		7.96 GB	SampleAC5_ILM1_ST23_50ng_LIB2_PT1.R1.fastq.gz	SampleAC5_ILM1_ST23_50ng_LIB2_PT1.R2.fastq.gz	SampleAC5_ILM1_ST23_50ng_LIB2_PT2.R1.fastq.gz	SampleAC5_ILM1_ST23_50ng_LIB2_PT2.R2.fastq.gz	SampleAC5_ILM1_ST23_50ng_LIB2_PT3.R1.fastq.gz	SampleAC5_ILM1_ST23_50ng_LIB2_PT3.R2.fastq.gz	SampleAC5_ILM1_ST23_50ng_LIB2_PT4.R1.fastq.gz	SampleAC5_ILM1_ST23_50ng_LIB2_PT4.R2.fastq.gz
Sample Spike-in	SAMN16786363	ILM1	SRX9719036	SRR13289952	NextSeq 550	SampleAC5_ILM1_ST23_50ng_LIB3	PAIRED	fastq		6.90 GB	SampleAC5_ILM1_ST23_50ng_LIB3_PT1.R1.fastq.gz	SampleAC5_ILM1_ST23_50ng_LIB3_PT1.R2.fastq.gz	SampleAC5_ILM1_ST23_50ng_LIB3_PT2.R1.fastq.gz	SampleAC5_ILM1_ST23_50ng_LIB3_PT2.R2.fastq.gz	SampleAC5_ILM1_ST23_50ng_LIB3_PT3.R1.fastq.gz	SampleAC5_ILM1_ST23_50ng_LIB3_PT3.R2.fastq.gz	SampleAC5_ILM1_ST23_50ng_LIB3_PT4.R1.fastq.gz	SampleAC5_ILM1_ST23_50ng_LIB3_PT4.R2.fastq.gz
Sample Spike-in	SAMN16786363	ILM1	SRX9719035	SRR13289953	NextSeq 550	SampleAC5_ILM1_ST23_50ng_LIB4	PAIRED	fastq		2.06 GB	SampleAC5_ILM1_ST23_50ng_LIB4_PT1.R1.fastq.gz	SampleAC5_ILM1_ST23_50ng_LIB4_PT1.R2.fastq.gz	SampleAC5_ILM1_ST23_50ng_LIB4_PT2.R1.fastq.gz	SampleAC5_ILM1_ST23_50ng_LIB4_PT2.R2.fastq.gz	SampleAC5_ILM1_ST23_50ng_LIB4_PT3.R1.fastq.gz	SampleAC5_ILM1_ST23_50ng_LIB4_PT3.R2.fastq.gz	SampleAC5_ILM1_ST23_50ng_LIB4_PT4.R1.fastq.gz	SampleAC5_ILM1_ST23_50ng_LIB4_PT4.R2.fastq.gz
Sample Spike-in	SAMN16786363	ILM1	SRX9719046	SRR13289942	NextSeq 550	SampleAC5_ILM1_ST29_50ng_LIB3	PAIRED	fastq		6.40 GB	SampleAC5_ILM1_ST29_50ng_LIB3_PT1.R1.fastq.gz	SampleAC5_ILM1_ST29_50ng_LIB3_PT1.R2.fastq.gz	SampleAC5_ILM1_ST29_50ng_LIB3_PT2.R1.fastq.gz	SampleAC5_ILM1_ST29_50ng_LIB3_PT2.R2.fastq.gz	SampleAC5_ILM1_ST29_50ng_LIB3_PT3.R1.fastq.gz	SampleAC5_ILM1_ST29_50ng_LIB3_PT3.R2.fastq.gz	SampleAC5_ILM1_ST29_50ng_LIB3_PT4.R1.fastq.gz	SampleAC5_ILM1_ST29_50ng_LIB3_PT4.R2.fastq.gz
Sample B	SAMN16786361	ILM1	SRX9638523	SRR13205290	NextSeq 550	SampleB_ILM1_ST10_50ng_LIB1	PAIRED	fastq		7.73 GB	SampleB_ILM1_ST10_50ng_LIB1_PT1.R1.fastq.gz	SampleB_ILM1_ST10_50ng_LIB1_PT1.R2.fastq.gz	SampleB_ILM1_ST10_50ng_LIB1_PT2.R1.fastq.gz	SampleB_ILM1_ST10_50ng_LIB1_PT2.R2.fastq.gz	SampleB_ILM1_ST10_50ng_LIB1_PT3.R1.fastq.gz	SampleB_ILM1_ST10_50ng_LIB1_PT3.R2.fastq.gz	SampleB_ILM1_ST10_50ng_LIB1_PT4.R1.fastq.gz	SampleB_ILM1_ST10_50ng_LIB1_PT4.R2.fastq.gz
Sample B	SAMN16786361	ILM1	SRX9638524	SRR13205289	NextSeq 550	SampleB_ILM1_ST10_50ng_LIB2	PAIRED	fastq		8.09 GB	SampleB_ILM1_ST10_50ng_LIB2_PT1.R1.fastq.gz	SampleB_ILM1_ST10_50ng_LIB2_PT1.R2.fastq.gz	SampleB_ILM1_ST10_50ng_LIB2_PT2.R1.fastq.gz	SampleB_ILM1_ST10_50ng_LIB2_PT2.R2.fastq.gz	SampleB_ILM1_ST10_50ng_LIB2_PT3.R1.fastq.gz	SampleB_ILM1_ST10_50ng_LIB2_PT3.R2.fastq.gz	SampleB_ILM1_ST10_50ng_LIB2_PT4.R1.fastq.gz	SampleB_ILM1_ST10_50ng_LIB2_PT4.R2.fastq.gz
Sample B	SAMN16786361	ILM1	SRX9638525	SRR13205288	NextSeq 550	SampleB_ILM1_ST10_50ng_LIB3	PAIRED	fastq		7.90 GB	SampleB_ILM1_ST10_50ng_LIB3_PT1.R1.fastq.gz	SampleB_ILM1_ST10_50ng_LIB3_PT1.R2.fastq.gz	SampleB_ILM1_ST10_50ng_LIB3_PT2.R1.fastq.gz	SampleB_ILM1_ST10_50ng_LIB3_PT2.R2.fastq.gz	SampleB_ILM1_ST10_50ng_LIB3_PT3.R1.fastq.gz	SampleB_ILM1_ST10_50ng_LIB3_PT3.R2.fastq.gz	SampleB_ILM1_ST10_50ng_LIB3_PT4.R1.fastq.gz	SampleB_ILM1_ST10_50ng_LIB3_PT4.R2.fastq.gz
Sample B	SAMN16786361	ILM1	SRX9638526	SRR13205287	NextSeq 550	SampleB_ILM1_ST10_50ng_LIB4	PAIRED	fastq		8.46 GB	SampleB_ILM1_ST10_50ng_LIB4_PT1.R1.fastq.gz	SampleB_ILM1_ST10_50ng_LIB4_PT1.R2.fastq.gz	SampleB_ILM1_ST10_50ng_LIB4_PT2.R1.fastq.gz	SampleB_ILM1_ST10_50ng_LIB4_PT2.R2.fastq.gz	SampleB_ILM1_ST10_50ng_LIB4_PT3.R1.fastq.gz	SampleB_ILM1_ST10_50ng_LIB4_PT3.R2.fastq.gz	SampleB_ILM1_ST10_50ng_LIB4_PT4.R1.fastq.gz	SampleB_ILM1_ST10_50ng_LIB4_PT4.R2.fastq.gz
Sample B	SAMN16786361	ILM1	SRX9719051	SRR13289937	NextSeq 550	SampleB_ILM1_ST12_50ng_LIB1	PAIRED	fastq		6.32 GB	SampleB_ILM1_ST12_50ng_LIB1_PT1.R1.fastq.gz	SampleB_ILM1_ST12_50ng_LIB1_PT1.R2.fastq.gz	SampleB_ILM1_ST12_50ng_LIB1_PT2.R1.fastq.gz	SampleB_ILM1_ST12_50ng_LIB1_PT2.R2.fastq.gz	SampleB_ILM1_ST12_50ng_LIB1_PT3.R1.fastq.gz	SampleB_ILM1_ST12_50ng_LIB1_PT3.R2.fastq.gz	SampleB_ILM1_ST12_50ng_LIB1_PT4.R1.fastq.gz	SampleB_ILM1_ST12_50ng_LIB1_PT4.R2.fastq.gz
Sample B	SAMN16786361	ILM1	SRX9719052	SRR13289936	NextSeq 550	SampleB_ILM1_ST12_50ng_LIB2	PAIRED	fastq		6.60 GB	SampleB_ILM1_ST12_50ng_LIB2_PT1.R1.fastq.gz	SampleB_ILM1_ST12_50ng_LIB2_PT1.R2.fastq.gz	SampleB_ILM1_ST12_50ng_LIB2_PT2.R1.fastq.gz	SampleB_ILM1_ST12_50ng_LIB2_PT2.R2.fastq.gz	SampleB_ILM1_ST12_50ng_LIB2_PT3.R1.fastq.gz	SampleB_ILM1_ST12_50ng_LIB2_PT3.R2.fastq.gz	SampleB_ILM1_ST12_50ng_LIB2_PT4.R1.fastq.gz	SampleB_ILM1_ST12_50ng_LIB2_PT4.R2.fastq.gz
Sample B	SAMN16786361	ILM1	SRX9719034	SRR13289954	NextSeq 550	SampleB_ILM1_ST23_50ng_LIB1	PAIRED	fastq		7.05 GB	SampleB_ILM1_ST23_50ng_LIB1_PT1.R1.fastq.gz	SampleB_ILM1_ST23_50ng_LIB1_PT1.R2.fastq.gz	SampleB_ILM1_ST23_50ng_LIB1_PT2.R1.fastq.gz	SampleB_ILM1_ST23_50ng_LIB1_PT2.R2.fastq.gz	SampleB_ILM1_ST23_50ng_LIB1_PT3.R1.fastq.gz	SampleB_ILM1_ST23_50ng_LIB1_PT3.R2.fastq.gz	SampleB_ILM1_ST23_50ng_LIB1_PT4.R1.fastq.gz	SampleB_ILM1_ST23_50ng_LIB1_PT4.R2.fastq.gz
Sample B	SAMN16786361	ILM1	SRX9719033	SRR13289955	NextSeq 550	SampleB_ILM1_ST23_50ng_LIB2	PAIRED	fastq		8.07 GB	SampleB_ILM1_ST23_50ng_LIB2_PT1.R1.fastq.gz	SampleB_ILM1_ST23_50ng_LIB2_PT1.R2.fastq.gz	SampleB_ILM1_ST23_50ng_LIB2_PT2.R1.fastq.gz	SampleB_ILM1_ST23_50ng_LIB2_PT2.R2.fastq.gz	SampleB_ILM1_ST23_50ng_LIB2_PT3.R1.fastq.gz	SampleB_ILM1_ST23_50ng_LIB2_PT3.R2.fastq.gz	SampleB_ILM1_ST23_50ng_LIB2_PT4.R1.fastq.gz	SampleB_ILM1_ST23_50ng_LIB2_PT4.R2.fastq.gz
Sample B	SAMN16786361	ILM1	SRX9719032	SRR13289956	NextSeq 550	SampleB_ILM1_ST23_50ng_LIB3	PAIRED	fastq		6.53 GB	SampleB_ILM1_ST23_50ng_LIB3_PT1.R1.fastq.gz	SampleB_ILM1_ST23_50ng_LIB3_PT1.R2.fastq.gz	SampleB_ILM1_ST23_50ng_LIB3_PT2.R1.fastq.gz	SampleB_ILM1_ST23_50ng_LIB3_PT2.R2.fastq.gz	SampleB_ILM1_ST23_50ng_LIB3_PT3.R1.fastq.gz	SampleB_ILM1_ST23_50ng_LIB3_PT3.R2.fastq.gz	SampleB_ILM1_ST23_50ng_LIB3_PT4.R1.fastq.gz	SampleB_ILM1_ST23_50ng_LIB3_PT4.R2.fastq.gz
Sample B	SAMN16786361	ILM1	SRX9719031	SRR13289957	NextSeq 550	SampleB_ILM1_ST23_50ng_LIB4	PAIRED	fastq		0.18 GB	SampleB_ILM1_ST23_50ng_LIB4_PT1.R1.fastq.gz	SampleB_ILM1_ST23_50ng_LIB4_PT1.R2.fastq.gz	SampleB_ILM1_ST23_50ng_LIB4_PT2.R1.fastq.gz	SampleB_ILM1_ST23_50ng_LIB4_PT2.R2.fastq.gz	SampleB_ILM1_ST23_50ng_LIB4_PT3.R1.fastq.gz	SampleB_ILM1_ST23_50ng_LIB4_PT3.R2.fastq.gz	SampleB_ILM1_ST23_50ng_LIB4_PT4.R1.fastq.gz	SampleB_ILM1_ST23_50ng_LIB4_PT4.R2.fastq.gz
Sample B	SAMN16786361	ILM1	SRX9719047	SRR13289941	NextSeq 550	SampleB_ILM1_ST29_50ng_LIB3	PAIRED	fastq		5.69 GB	SampleB_ILM1_ST29_50ng_LIB3_PT1.R1.fastq.gz	SampleB_ILM1_ST29_50ng_LIB3_PT1.R2.fastq.gz	SampleB_ILM1_ST29_50ng_LIB3_PT2.R1.fastq.gz	SampleB_ILM1_ST29_50ng_LIB3_PT2.R2.fastq.gz	SampleB_ILM1_ST29_50ng_LIB3_PT3.R1.fastq.gz	SampleB_ILM1_ST29_50ng_LIB3_PT3.R2.fastq.gz	SampleB_ILM1_ST29_50ng_LIB3_PT4.R1.fastq.gz	SampleB_ILM1_ST29_50ng_LIB3_PT4.R2.fastq.gz
Sample C	SAMN16786362	ILM1	SRX9638527	SRR13205286	NextSeq 550	SampleC_ILM1_ST10_50ng_LIB1	PAIRED	fastq		8.10 GB	SampleC_ILM1_ST10_50ng_LIB1_PT1.R1.fastq.gz	SampleC_ILM1_ST10_50ng_LIB1_PT1.R2.fastq.gz	SampleC_ILM1_ST10_50ng_LIB1_PT2.R1.fastq.gz	SampleC_ILM1_ST10_50ng_LIB1_PT2.R2.fastq.gz	SampleC_ILM1_ST10_50ng_LIB1_PT3.R1.fastq.gz	SampleC_ILM1_ST10_50ng_LIB1_PT3.R2.fastq.gz	SampleC_ILM1_ST10_50ng_LIB1_PT4.R1.fastq.gz	SampleC_ILM1_ST10_50ng_LIB1_PT4.R2.fastq.gz
Sample C	SAMN16786362	ILM1	SRX9638528	SRR13205285	NextSeq 550	SampleC_ILM1_ST10_50ng_LIB2	PAIRED	fastq		8.20 GB	SampleC_ILM1_ST10_50ng_LIB2_PT1.R1.fastq.gz	SampleC_ILM1_ST10_50ng_LIB2_PT1.R2.fastq.gz	SampleC_ILM1_ST10_50ng_LIB2_PT2.R1.fastq.gz	SampleC_ILM1_ST10_50ng_LIB2_PT2.R2.fastq.gz	SampleC_ILM1_ST10_50ng_LIB2_PT3.R1.fastq.gz	SampleC_ILM1_ST10_50ng_LIB2_PT3.R2.fastq.gz	SampleC_ILM1_ST10_50ng_LIB2_PT4.R1.fastq.gz	SampleC_ILM1_ST10_50ng_LIB2_PT4.R2.fastq.gz
Sample C	SAMN16786362	ILM1	SRX9638529	SRR13205284	NextSeq 550	SampleC_ILM1_ST10_50ng_LIB3	PAIRED	fastq		7.92 GB	SampleC_ILM1_ST10_50ng_LIB3_PT1.R1.fastq.gz	SampleC_ILM1_ST10_50ng_LIB3_PT1.R2.fastq.gz	SampleC_ILM1_ST10_50ng_LIB3_PT2.R1.fastq.gz	SampleC_ILM1_ST10_50ng_LIB3_PT2.R2.fastq.gz	SampleC_ILM1_ST10_50ng_LIB3_PT3.R1.fastq.gz	SampleC_ILM1_ST10_50ng_LIB3_PT3.R2.fastq.gz	SampleC_ILM1_ST10_50ng_LIB3_PT4.R1.fastq.gz	SampleC_ILM1_ST10_50ng_LIB3_PT4.R2.fastq.gz
Sample C	SAMN16786362	ILM1	SRX9638530	SRR13205283	NextSeq 550	SampleC_ILM1_ST10_50ng_LIB4	PAIRED	fastq		9.26 GB	SampleC_ILM1_ST10_50ng_LIB4_PT1.R1.fastq.gz	SampleC_ILM1_ST10_50ng_LIB4_PT1.R2.fastq.gz	SampleC_ILM1_ST10_50ng_LIB4_PT2.R1.fastq.gz	SampleC_ILM1_ST10_50ng_LIB4_PT2.R2.fastq.gz	SampleC_ILM1_ST10_50ng_LIB4_PT3.R1.fastq.gz	SampleC_ILM1_ST10_50ng_LIB4_PT3.R2.fastq.gz	SampleC_ILM1_ST10_50ng_LIB4_PT4.R1.fastq.gz	SampleC_ILM1_ST10_50ng_LIB4_PT4.R2.fastq.gz
Sample C	SAMN16786362	ILM1	SRX9719053	SRR13289935	NextSeq 550	SampleC_ILM1_ST12_50ng_LIB1	PAIRED	fastq		13.10 GB	SampleC_ILM1_ST12_50ng_LIB1_PT1.R1.fastq.gz	SampleC_ILM1_ST12_50ng_LIB1_PT1.R2.fastq.gz	SampleC_ILM1_ST12_50ng_LIB1_PT2.R1.fastq.gz	SampleC_ILM1_ST12_50ng_LIB1_PT2.R2.fastq.gz	SampleC_ILM1_ST12_50ng_LIB1_PT3.R1.fastq.gz	SampleC_ILM1_ST12_50ng_LIB1_PT3.R2.fastq.gz	SampleC_ILM1_ST12_50ng_LIB1_PT4.R1.fastq.gz	SampleC_ILM1_ST12_50ng_LIB1_PT4.R2.fastq.gz
Sample C	SAMN16786362	ILM1	SRX9719054	SRR13289934	NextSeq 550	SampleC_ILM1_ST12_50ng_LIB2	PAIRED	fastq		13.39 GB	SampleC_ILM1_ST12_50ng_LIB2_PT1.R1.fastq.gz	SampleC_ILM1_ST12_50ng_LIB2_PT1.R2.fastq.gz	SampleC_ILM1_ST12_50ng_LIB2_PT2.R1.fastq.gz	SampleC_ILM1_ST12_50ng_LIB2_PT2.R2.fastq.gz	SampleC_ILM1_ST12_50ng_LIB2_PT3.R1.fastq.gz	SampleC_ILM1_ST12_50ng_LIB2_PT3.R2.fastq.gz	SampleC_ILM1_ST12_50ng_LIB2_PT4.R1.fastq.gz	SampleC_ILM1_ST12_50ng_LIB2_PT4.R2.fastq.gz
Sample C	SAMN16786362	ILM1	SRX9719030	SRR13289958	NextSeq 550	SampleC_ILM1_ST23_50ng_LIB1	PAIRED	fastq		6.57 GB	SampleC_ILM1_ST23_50ng_LIB1_PT1.R1.fastq.gz	SampleC_ILM1_ST23_50ng_LIB1_PT1.R2.fastq.gz	SampleC_ILM1_ST23_50ng_LIB1_PT2.R1.fastq.gz	SampleC_ILM1_ST23_50ng_LIB1_PT2.R2.fastq.gz	SampleC_ILM1_ST23_50ng_LIB1_PT3.R1.fastq.gz	SampleC_ILM1_ST23_50ng_LIB1_PT3.R2.fastq.gz	SampleC_ILM1_ST23_50ng_LIB1_PT4.R1.fastq.gz	SampleC_ILM1_ST23_50ng_LIB1_PT4.R2.fastq.gz
Sample C	SAMN16786362	ILM1	SRX9719029	SRR13289959	NextSeq 550	SampleC_ILM1_ST23_50ng_LIB2	PAIRED	fastq		8.60 GB	SampleC_ILM1_ST23_50ng_LIB2_PT1.R1.fastq.gz	SampleC_ILM1_ST23_50ng_LIB2_PT1.R2.fastq.gz	SampleC_ILM1_ST23_50ng_LIB2_PT2.R1.fastq.gz	SampleC_ILM1_ST23_50ng_LIB2_PT2.R2.fastq.gz	SampleC_ILM1_ST23_50ng_LIB2_PT3.R1.fastq.gz	SampleC_ILM1_ST23_50ng_LIB2_PT3.R2.fastq.gz	SampleC_ILM1_ST23_50ng_LIB2_PT4.R1.fastq.gz	SampleC_ILM1_ST23_50ng_LIB2_PT4.R2.fastq.gz
Sample C	SAMN16786362	ILM1	SRX9719028	SRR13289960	NextSeq 550	SampleC_ILM1_ST23_50ng_LIB3	PAIRED	fastq		6.70 GB	SampleC_ILM1_ST23_50ng_LIB3_PT1.R1.fastq.gz	SampleC_ILM1_ST23_50ng_LIB3_PT1.R2.fastq.gz	SampleC_ILM1_ST23_50ng_LIB3_PT2.R1.fastq.gz	SampleC_ILM1_ST23_50ng_LIB3_PT2.R2.fastq.gz	SampleC_ILM1_ST23_50ng_LIB3_PT3.R1.fastq.gz	SampleC_ILM1_ST23_50ng_LIB3_PT3.R2.fastq.gz	SampleC_ILM1_ST23_50ng_LIB3_PT4.R1.fastq.gz	SampleC_ILM1_ST23_50ng_LIB3_PT4.R2.fastq.gz
Sample C	SAMN16786362	ILM1	SRX9719027	SRR13289961	NextSeq 550	SampleC_ILM1_ST23_50ng_LIB4	PAIRED	fastq		0.63 GB	SampleC_ILM1_ST23_50ng_LIB4_PT1.R1.fastq.gz	SampleC_ILM1_ST23_50ng_LIB4_PT1.R2.fastq.gz	SampleC_ILM1_ST23_50ng_LIB4_PT2.R1.fastq.gz	SampleC_ILM1_ST23_50ng_LIB4_PT2.R2.fastq.gz	SampleC_ILM1_ST23_50ng_LIB4_PT3.R1.fastq.gz	SampleC_ILM1_ST23_50ng_LIB4_PT3.R2.fastq.gz	SampleC_ILM1_ST23_50ng_LIB4_PT4.R1.fastq.gz	SampleC_ILM1_ST23_50ng_LIB4_PT4.R2.fastq.gz
Sample C	SAMN16786362	ILM1	SRX9719048	SRR13289940	NextSeq 550	SampleC_ILM1_ST29_50ng_LIB3	PAIRED	fastq		5.76 GB	SampleC_ILM1_ST29_50ng_LIB3_PT1.R1.fastq.gz	SampleC_ILM1_ST29_50ng_LIB3_PT1.R2.fastq.gz	SampleC_ILM1_ST29_50ng_LIB3_PT2.R1.fastq.gz	SampleC_ILM1_ST29_50ng_LIB3_PT2.R2.fastq.gz	SampleC_ILM1_ST29_50ng_LIB3_PT3.R1.fastq.gz	SampleC_ILM1_ST29_50ng_LIB3_PT3.R2.fastq.gz	SampleC_ILM1_ST29_50ng_LIB3_PT4.R1.fastq.gz	SampleC_ILM1_ST29_50ng_LIB3_PT4.R2.fastq.gz
Sample A	SAMN16786360	QGN1	SRX9657102	SRR13224722	Illumina NovaSeq 6000	SampleA_QGN1_ST13_40ng_LIB1	PAIRED	fastq		29.08 GB	SampleA_QGN1_ST13_40ng_LIB1.R1.fastq.gz	SampleA_QGN1_ST13_40ng_LIB1.R2.fastq.gz						
Sample A	SAMN16786360	QGN1	SRX9657103	SRR13224721	Illumina NovaSeq 6000	SampleA_QGN1_ST13_40ng_LIB2	PAIRED	fastq		15.89 GB	SampleA_QGN1_ST13_40ng_LIB2.R1.fastq.gz	SampleA_QGN1_ST13_40ng_LIB2.R2.fastq.gz						
Sample A	SAMN16786360	QGN1	SRX9657114	SRR13224710	Illumina NovaSeq 6000	SampleA_QGN1_ST13_40ng_LIB3	PAIRED	fastq		30.17 GB	SampleA_QGN1_ST13_40ng_LIB3.R1.fastq.gz	SampleA_QGN1_ST13_40ng_LIB3.R2.fastq.gz						
Sample A	SAMN16786360	QGN1	SRX9657125	SRR13224699	Illumina NovaSeq 6000	SampleA_QGN1_ST13_40ng_LIB4	PAIRED	fastq		19.92 GB	SampleA_QGN1_ST13_40ng_LIB4.R1.fastq.gz	SampleA_QGN1_ST13_40ng_LIB4.R2.fastq.gz						
Sample A	SAMN16786360	QGN1	SRX9657115	SRR13224709	Illumina NovaSeq 6000	SampleA_QGN1_ST14_25ng_LIB1	PAIRED	fastq		11.42 GB	SampleA_QGN1_ST14_25ng_LIB1.R1.fastq.gz	SampleA_QGN1_ST14_25ng_LIB1.R2.fastq.gz						
Sample A	SAMN16786360	QGN1	SRX9657116	SRR13224708	Illumina NovaSeq 6000	SampleA_QGN1_ST14_25ng_LIB2	PAIRED	fastq		13.11 GB	SampleA_QGN1_ST14_25ng_LIB2.R1.fastq.gz	SampleA_QGN1_ST14_25ng_LIB2.R2.fastq.gz						
Sample A	SAMN16786360	QGN1	SRX9657117	SRR13224707	Illumina NovaSeq 6000	SampleA_QGN1_ST14_25ng_LIB3	PAIRED	fastq		9.82 GB	SampleA_QGN1_ST14_25ng_LIB3.R1.fastq.gz	SampleA_QGN1_ST14_25ng_LIB3.R2.fastq.gz						
Sample A	SAMN16786360	QGN1	SRX9657118	SRR13224706	Illumina NovaSeq 6000	SampleA_QGN1_ST14_25ng_LIB4	PAIRED	fastq		20.12 GB	SampleA_QGN1_ST14_25ng_LIB4.R1.fastq.gz	SampleA_QGN1_ST14_25ng_LIB4.R2.fastq.gz						
Sample A	SAMN16786360	QGN1	SRX9657137	SRR13224687	Illumina NovaSeq 6000	SampleA_QGN1_ST15_25ng_LIB1	PAIRED	fastq		11.23 GB	SampleA_QGN1_ST15_25ng_LIB1.R1.fastq.gz	SampleA_QGN1_ST15_25ng_LIB1.R2.fastq.gz						
Sample A	SAMN16786360	QGN1	SRX9657138	SRR13224686	Illumina NovaSeq 6000	SampleA_QGN1_ST15_25ng_LIB2	PAIRED	fastq		15.17 GB	SampleA_QGN1_ST15_25ng_LIB2.R1.fastq.gz	SampleA_QGN1_ST15_25ng_LIB2.R2.fastq.gz						
Sample A	SAMN16786360	QGN1	SRX9657139	SRR13224685	Illumina NovaSeq 6000	SampleA_QGN1_ST15_25ng_LIB3	PAIRED	fastq		11.51 GB	SampleA_QGN1_ST15_25ng_LIB3.R1.fastq.gz	SampleA_QGN1_ST15_25ng_LIB3.R2.fastq.gz						
Sample A	SAMN16786360	QGN1	SRX9657140	SRR13224684	Illumina NovaSeq 6000	SampleA_QGN1_ST15_25ng_LIB4	PAIRED	fastq		12.49 GB	SampleA_QGN1_ST15_25ng_LIB4.R1.fastq.gz	SampleA_QGN1_ST15_25ng_LIB4.R2.fastq.gz						
Sample Spike-in	SAMN16786363	QGN1	SRX9657160	SRR13224664	Illumina NovaSeq 6000	SampleAC5_QGN1_ST13_40ng_LIB1	PAIRED	fastq		13.00 GB	SampleAC5_QGN1_ST13_40ng_LIB1.R1.fastq.gz	SampleAC5_QGN1_ST13_40ng_LIB1.R2.fastq.gz						
Sample Spike-in	SAMN16786363	QGN1	SRX9657161	SRR13224663	Illumina NovaSeq 6000	SampleAC5_QGN1_ST13_40ng_LIB2	PAIRED	fastq		13.94 GB	SampleAC5_QGN1_ST13_40ng_LIB2.R1.fastq.gz	SampleAC5_QGN1_ST13_40ng_LIB2.R2.fastq.gz						
Sample Spike-in	SAMN16786363	QGN1	SRX9657104	SRR13224720	Illumina NovaSeq 6000	SampleAC5_QGN1_ST13_40ng_LIB3	PAIRED	fastq		8.58 GB	SampleAC5_QGN1_ST13_40ng_LIB3.R1.fastq.gz	SampleAC5_QGN1_ST13_40ng_LIB3.R2.fastq.gz						
Sample Spike-in	SAMN16786363	QGN1	SRX9657105	SRR13224719	Illumina NovaSeq 6000	SampleAC5_QGN1_ST13_40ng_LIB4	PAIRED	fastq		16.21 GB	SampleAC5_QGN1_ST13_40ng_LIB4.R1.fastq.gz	SampleAC5_QGN1_ST13_40ng_LIB4.R2.fastq.gz						
Sample Spike-in	SAMN16786363	QGN1	SRX9657123	SRR13224701	Illumina NovaSeq 6000	SampleAC5_QGN1_ST14_25ng_LIB1	PAIRED	fastq		12.92 GB	SampleAC5_QGN1_ST14_25ng_LIB1.R1.fastq.gz	SampleAC5_QGN1_ST14_25ng_LIB1.R2.fastq.gz						
Sample Spike-in	SAMN16786363	QGN1	SRX9657124	SRR13224700	Illumina NovaSeq 6000	SampleAC5_QGN1_ST14_25ng_LIB2	PAIRED	fastq		9.45 GB	SampleAC5_QGN1_ST14_25ng_LIB2.R1.fastq.gz	SampleAC5_QGN1_ST14_25ng_LIB2.R2.fastq.gz						
Sample Spike-in	SAMN16786363	QGN1	SRX9657126	SRR13224698	Illumina NovaSeq 6000	SampleAC5_QGN1_ST14_25ng_LIB3	PAIRED	fastq		12.62 GB	SampleAC5_QGN1_ST14_25ng_LIB3.R1.fastq.gz	SampleAC5_QGN1_ST14_25ng_LIB3.R2.fastq.gz						
Sample Spike-in	SAMN16786363	QGN1	SRX9657127	SRR13224697	Illumina NovaSeq 6000	SampleAC5_QGN1_ST14_25ng_LIB4	PAIRED	fastq		11.14 GB	SampleAC5_QGN1_ST14_25ng_LIB4.R1.fastq.gz	SampleAC5_QGN1_ST14_25ng_LIB4.R2.fastq.gz						
Sample Spike-in	SAMN16786363	QGN1	SRX9657145	SRR13224679	Illumina NovaSeq 6000	SampleAC5_QGN1_ST15_25ng_LIB1	PAIRED	fastq		11.35 GB	SampleAC5_QGN1_ST15_25ng_LIB1.R1.fastq.gz	SampleAC5_QGN1_ST15_25ng_LIB1.R2.fastq.gz						
Sample Spike-in	SAMN16786363	QGN1	SRX9657146	SRR13224678	Illumina NovaSeq 6000	SampleAC5_QGN1_ST15_25ng_LIB2	PAIRED	fastq		13.51 GB	SampleAC5_QGN1_ST15_25ng_LIB2.R1.fastq.gz	SampleAC5_QGN1_ST15_25ng_LIB2.R2.fastq.gz						
Sample Spike-in	SAMN16786363	QGN1	SRX9657148	SRR13224676	Illumina NovaSeq 6000	SampleAC5_QGN1_ST15_25ng_LIB3	PAIRED	fastq		12.40 GB	SampleAC5_QGN1_ST15_25ng_LIB3.R1.fastq.gz	SampleAC5_QGN1_ST15_25ng_LIB3.R2.fastq.gz						
Sample Spike-in	SAMN16786363	QGN1	SRX9657149	SRR13224675	Illumina NovaSeq 6000	SampleAC5_QGN1_ST15_25ng_LIB4	PAIRED	fastq		12.21 GB	SampleAC5_QGN1_ST15_25ng_LIB4.R1.fastq.gz	SampleAC5_QGN1_ST15_25ng_LIB4.R2.fastq.gz						
Sample B	SAMN16786361	QGN1	SRX9657106	SRR13224718	Illumina NovaSeq 6000	SampleB_QGN1_ST13_40ng_LIB1	PAIRED	fastq		11.54 GB	SampleB_QGN1_ST13_40ng_LIB1.R1.fastq.gz	SampleB_QGN1_ST13_40ng_LIB1.R2.fastq.gz						
Sample B	SAMN16786361	QGN1	SRX9657107	SRR13224717	Illumina NovaSeq 6000	SampleB_QGN1_ST13_40ng_LIB2	PAIRED	fastq		11.80 GB	SampleB_QGN1_ST13_40ng_LIB2.R1.fastq.gz	SampleB_QGN1_ST13_40ng_LIB2.R2.fastq.gz						
Sample B	SAMN16786361	QGN1	SRX9657108	SRR13224716	Illumina NovaSeq 6000	SampleB_QGN1_ST13_40ng_LIB3	PAIRED	fastq		10.81 GB	SampleB_QGN1_ST13_40ng_LIB3.R1.fastq.gz	SampleB_QGN1_ST13_40ng_LIB3.R2.fastq.gz						
Sample B	SAMN16786361	QGN1	SRX9657109	SRR13224715	Illumina NovaSeq 6000	SampleB_QGN1_ST13_40ng_LIB4	PAIRED	fastq		9.98 GB	SampleB_QGN1_ST13_40ng_LIB4.R1.fastq.gz	SampleB_QGN1_ST13_40ng_LIB4.R2.fastq.gz						
Sample B	SAMN16786361	QGN1	SRX9657128	SRR13224696	Illumina NovaSeq 6000	SampleB_QGN1_ST14_25ng_LIB1	PAIRED	fastq		13.91 GB	SampleB_QGN1_ST14_25ng_LIB1.R1.fastq.gz	SampleB_QGN1_ST14_25ng_LIB1.R2.fastq.gz						
Sample B	SAMN16786361	QGN1	SRX9657129	SRR13224695	Illumina NovaSeq 6000	SampleB_QGN1_ST14_25ng_LIB2	PAIRED	fastq		9.75 GB	SampleB_QGN1_ST14_25ng_LIB2.R1.fastq.gz	SampleB_QGN1_ST14_25ng_LIB2.R2.fastq.gz						
Sample B	SAMN16786361	QGN1	SRX9657130	SRR13224694	Illumina NovaSeq 6000	SampleB_QGN1_ST14_25ng_LIB3	PAIRED	fastq		11.60 GB	SampleB_QGN1_ST14_25ng_LIB3.R1.fastq.gz	SampleB_QGN1_ST14_25ng_LIB3.R2.fastq.gz						
Sample B	SAMN16786361	QGN1	SRX9657131	SRR13224693	Illumina NovaSeq 6000	SampleB_QGN1_ST14_25ng_LIB4	PAIRED	fastq		10.08 GB	SampleB_QGN1_ST14_25ng_LIB4.R1.fastq.gz	SampleB_QGN1_ST14_25ng_LIB4.R2.fastq.gz						
Sample B	SAMN16786361	QGN1	SRX9657150	SRR13224674	Illumina NovaSeq 6000	SampleB_QGN1_ST15_25ng_LIB1	PAIRED	fastq		14.40 GB	SampleB_QGN1_ST15_25ng_LIB1.R1.fastq.gz	SampleB_QGN1_ST15_25ng_LIB1.R2.fastq.gz						
Sample B	SAMN16786361	QGN1	SRX9657151	SRR13224673	Illumina NovaSeq 6000	SampleB_QGN1_ST15_25ng_LIB2	PAIRED	fastq		9.71 GB	SampleB_QGN1_ST15_25ng_LIB2.R1.fastq.gz	SampleB_QGN1_ST15_25ng_LIB2.R2.fastq.gz						
Sample B	SAMN16786361	QGN1	SRX9657152	SRR13224672	Illumina NovaSeq 6000	SampleB_QGN1_ST15_25ng_LIB3	PAIRED	fastq		11.46 GB	SampleB_QGN1_ST15_25ng_LIB3.R1.fastq.gz	SampleB_QGN1_ST15_25ng_LIB3.R2.fastq.gz						
Sample B	SAMN16786361	QGN1	SRX9657153	SRR13224671	Illumina NovaSeq 6000	SampleB_QGN1_ST15_25ng_LIB4	PAIRED	fastq		11.07 GB	SampleB_QGN1_ST15_25ng_LIB4.R1.fastq.gz	SampleB_QGN1_ST15_25ng_LIB4.R2.fastq.gz						
Sample C	SAMN16786362	QGN1	SRX9657110	SRR13224714	Illumina NovaSeq 6000	SampleC_QGN1_ST13_40ng_LIB1	PAIRED	fastq		20.47 GB	SampleC_QGN1_ST13_40ng_LIB1.R1.fastq.gz	SampleC_QGN1_ST13_40ng_LIB1.R2.fastq.gz						
Sample C	SAMN16786362	QGN1	SRX9657111	SRR13224713	Illumina NovaSeq 6000	SampleC_QGN1_ST13_40ng_LIB2	PAIRED	fastq		23.53 GB	SampleC_QGN1_ST13_40ng_LIB2.R1.fastq.gz	SampleC_QGN1_ST13_40ng_LIB2.R2.fastq.gz						
Sample C	SAMN16786362	QGN1	SRX9657112	SRR13224712	Illumina NovaSeq 6000	SampleC_QGN1_ST13_40ng_LIB3	PAIRED	fastq		14.02 GB	SampleC_QGN1_ST13_40ng_LIB3.R1.fastq.gz	SampleC_QGN1_ST13_40ng_LIB3.R2.fastq.gz						
Sample C	SAMN16786362	QGN1	SRX9657113	SRR13224711	Illumina NovaSeq 6000	SampleC_QGN1_ST13_40ng_LIB4	PAIRED	fastq		20.13 GB	SampleC_QGN1_ST13_40ng_LIB4.R1.fastq.gz	SampleC_QGN1_ST13_40ng_LIB4.R2.fastq.gz						
Sample C	SAMN16786362	QGN1	SRX9657132	SRR13224692	Illumina NovaSeq 6000	SampleC_QGN1_ST14_25ng_LIB1	PAIRED	fastq		21.31 GB	SampleC_QGN1_ST14_25ng_LIB1.R1.fastq.gz	SampleC_QGN1_ST14_25ng_LIB1.R2.fastq.gz						
Sample C	SAMN16786362	QGN1	SRX9657133	SRR13224691	Illumina NovaSeq 6000	SampleC_QGN1_ST14_25ng_LIB2	PAIRED	fastq		20.91 GB	SampleC_QGN1_ST14_25ng_LIB2.R1.fastq.gz	SampleC_QGN1_ST14_25ng_LIB2.R2.fastq.gz						
Sample C	SAMN16786362	QGN1	SRX9657134	SRR13224690	Illumina NovaSeq 6000	SampleC_QGN1_ST14_25ng_LIB3	PAIRED	fastq		25.03 GB	SampleC_QGN1_ST14_25ng_LIB3.R1.fastq.gz	SampleC_QGN1_ST14_25ng_LIB3.R2.fastq.gz						
Sample C	SAMN16786362	QGN1	SRX9657135	SRR13224689	Illumina NovaSeq 6000	SampleC_QGN1_ST14_25ng_LIB4	PAIRED	fastq		37.50 GB	SampleC_QGN1_ST14_25ng_LIB4.R1.fastq.gz	SampleC_QGN1_ST14_25ng_LIB4.R2.fastq.gz						
Sample C	SAMN16786362	QGN1	SRX9657154	SRR13224670	Illumina NovaSeq 6000	SampleC_QGN1_ST15_25ng_LIB1	PAIRED	fastq		23.52 GB	SampleC_QGN1_ST15_25ng_LIB1.R1.fastq.gz	SampleC_QGN1_ST15_25ng_LIB1.R2.fastq.gz						
Sample C	SAMN16786362	QGN1	SRX9657155	SRR13224669	Illumina NovaSeq 6000	SampleC_QGN1_ST15_25ng_LIB2	PAIRED	fastq		27.48 GB	SampleC_QGN1_ST15_25ng_LIB2.R1.fastq.gz	SampleC_QGN1_ST15_25ng_LIB2.R2.fastq.gz						
Sample C	SAMN16786362	QGN1	SRX9657156	SRR13224668	Illumina NovaSeq 6000	SampleC_QGN1_ST15_25ng_LIB3	PAIRED	fastq		24.67 GB	SampleC_QGN1_ST15_25ng_LIB3.R1.fastq.gz	SampleC_QGN1_ST15_25ng_LIB3.R2.fastq.gz						
Sample C	SAMN16786362	QGN1	SRX9657157	SRR13224667	Illumina NovaSeq 6000	SampleC_QGN1_ST15_25ng_LIB4	PAIRED	fastq		36.12 GB	SampleC_QGN1_ST15_25ng_LIB4.R1.fastq.gz	SampleC_QGN1_ST15_25ng_LIB4.R2.fastq.gz						
Sample A	SAMN16786360	ROC1	SRX9805935	SRR13386727	Illumina NovaSeq 6000	SampleA_ROC1_ST16_100ng_LIB1	PAIRED	bam	unmapped	26.37 GB	SampleA_ROC1_ST16_100ng_LIB1.bam							
Sample A	SAMN16786360	ROC1	SRX9805936	SRR13386726	Illumina NovaSeq 6000	SampleA_ROC1_ST16_100ng_LIB2	PAIRED	bam	unmapped	24.19 GB	SampleA_ROC1_ST16_100ng_LIB2.bam							
Sample A	SAMN16786360	ROC1	SRX9805947	SRR13386715	Illumina NovaSeq 6000	SampleA_ROC1_ST16_100ng_LIB3	PAIRED	bam	unmapped	18.47 GB	SampleA_ROC1_ST16_100ng_LIB3.bam							
Sample A	SAMN16786360	ROC1	SRX9805958	SRR13386704	Illumina NovaSeq 6000	SampleA_ROC1_ST16_100ng_LIB4	PAIRED	bam	unmapped	18.75 GB	SampleA_ROC1_ST16_100ng_LIB4.bam							
Sample A	SAMN16786360	ROC1	SRX9805948	SRR13386714	Illumina NovaSeq 6000	SampleA_ROC1_ST17_100ng_LIB1	PAIRED	bam	unmapped	21.18 GB	SampleA_ROC1_ST17_100ng_LIB1.bam							
Sample A	SAMN16786360	ROC1	SRX9805949	SRR13386713	Illumina NovaSeq 6000	SampleA_ROC1_ST17_100ng_LIB2	PAIRED	bam	unmapped	26.89 GB	SampleA_ROC1_ST17_100ng_LIB2.bam							
Sample A	SAMN16786360	ROC1	SRX9805950	SRR13386712	Illumina NovaSeq 6000	SampleA_ROC1_ST17_100ng_LIB3	PAIRED	bam	unmapped	28.95 GB	SampleA_ROC1_ST17_100ng_LIB3.bam							
Sample A	SAMN16786360	ROC1	SRX9805951	SRR13386711	Illumina NovaSeq 6000	SampleA_ROC1_ST17_100ng_LIB4	PAIRED	bam	unmapped	25.69 GB	SampleA_ROC1_ST17_100ng_LIB4.bam							
Sample A	SAMN16786360	ROC1	SRX9805990	SRR13386671	Illumina NovaSeq 6000	SampleA_ROC1_ST18_100ng_LIB1	PAIRED	bam	unmapped	16.84 GB	SampleA_ROC1_ST18_100ng_LIB1.bam							
Sample A	SAMN16786360	ROC1	SRX9805991	SRR13386670	Illumina NovaSeq 6000	SampleA_ROC1_ST18_100ng_LIB2	PAIRED	bam	unmapped	18.64 GB	SampleA_ROC1_ST18_100ng_LIB2.bam							
Sample A	SAMN16786360	ROC1	SRX9805992	SRR13386669	Illumina NovaSeq 6000	SampleA_ROC1_ST18_100ng_LIB3	PAIRED	bam	unmapped	19.17 GB	SampleA_ROC1_ST18_100ng_LIB3.bam							
Sample A	SAMN16786360	ROC1	SRX9805993	SRR13386668	Illumina NovaSeq 6000	SampleA_ROC1_ST18_100ng_LIB4	PAIRED	bam	unmapped	17.55 GB	SampleA_ROC1_ST18_100ng_LIB4.bam							
Sample A	SAMN16786360	ROC1	SRX9806012	SRR13386649	Illumina NovaSeq 6000	SampleA_ROC1_ST19_100ng_LIB1	PAIRED	bam	unmapped	24.34 GB	SampleA_ROC1_ST19_100ng_LIB1.bam							
Sample A	SAMN16786360	ROC1	SRX9806013	SRR13386648	Illumina NovaSeq 6000	SampleA_ROC1_ST19_100ng_LIB2	PAIRED	bam	unmapped	17.22 GB	SampleA_ROC1_ST19_100ng_LIB2.bam							
Sample A	SAMN16786360	ROC1	SRX9806014	SRR13386696	Illumina NovaSeq 6000	SampleA_ROC1_ST19_100ng_LIB3	PAIRED	bam	unmapped	21.32 GB	SampleA_ROC1_ST19_100ng_LIB3.bam							
Sample A	SAMN16786360	ROC1	SRX9805967	SRR13386695	Illumina NovaSeq 6000	SampleA_ROC1_ST19_100ng_LIB4	PAIRED	bam	unmapped	24.40 GB	SampleA_ROC1_ST19_100ng_LIB4.bam							
Sample Spike-in	SAMN16786363	ROC1	SRX9805985	SRR13386677	Illumina NovaSeq 6000	SampleAC5_ROC1_ST16_100ng_LIB1	PAIRED	bam	unmapped	24.54 GB	SampleAC5_ROC1_ST16_100ng_LIB1.bam							
Sample Spike-in	SAMN16786363	ROC1	SRX9805986	SRR13386675	Illumina NovaSeq 6000	SampleAC5_ROC1_ST16_100ng_LIB2	PAIRED	bam	unmapped	18.06 GB	SampleAC5_ROC1_ST16_100ng_LIB2.bam							
Sample Spike-in	SAMN16786363	ROC1	SRX9805937	SRR13386725	Illumina NovaSeq 6000	SampleAC5_ROC1_ST16_100ng_LIB3	PAIRED	bam	unmapped	18.47 GB	SampleAC5_ROC1_ST16_100ng_LIB3.bam							
Sample Spike-in	SAMN16786363	ROC1	SRX9805938	SRR13386724	Illumina NovaSeq 6000	SampleAC5_ROC1_ST16_100ng_LIB4	PAIRED	bam	unmapped	18.67 GB	SampleAC5_ROC1_ST16_100ng_LIB4.bam							
Sample Spike-in	SAMN16786363	ROC1	SRX9805956	SRR13386706	Illumina NovaSeq 6000	SampleAC5_ROC1_ST17_100ng_LIB1	PAIRED	bam	unmapped	21.20 GB	SampleAC5_ROC1_ST17_100ng_LIB1.bam							
Sample Spike-in	SAMN16786363	ROC1	SRX9805957	SRR13386705	Illumina NovaSeq 6000	SampleAC5_ROC1_ST17_100ng_LIB2	PAIRED	bam	unmapped	18.68 GB	SampleAC5_ROC1_ST17_100ng_LIB2.bam							
Sample Spike-in	SAMN16786363	ROC1	SRX9805959	SRR13386703	Illumina NovaSeq 6000	SampleAC5_ROC1_ST17_100ng_LIB3	PAIRED	bam	unmapped	28.75 GB	SampleAC5_ROC1_ST17_100ng_LIB3.bam							
Sample Spike-in	SAMN16786363	ROC1	SRX9805960	SRR13386702	Illumina NovaSeq 6000	SampleAC5_ROC1_ST17_100ng_LIB4	PAIRED	bam	unmapped	18.32 GB	SampleAC5_ROC1_ST17_100ng_LIB4.bam							
Sample Spike-in	SAMN16786363	ROC1	SRX9805998	SRR13386663	Illumina NovaSeq 6000	SampleAC5_ROC1_ST18_100ng_LIB1	PAIRED	bam	unmapped	19.73 GB	SampleAC5_ROC1_ST18_100ng_LIB1.bam							
Sample Spike-in	SAMN16786363	ROC1	SRX9805999	SRR13386662	Illumina NovaSeq 6000	SampleAC5_ROC1_ST18_100ng_LIB2	PAIRED	bam	unmapped	16.98 GB	SampleAC5_ROC1_ST18_100ng_LIB2.bam							
Sample Spike-in	SAMN16786363	ROC1	SRX9806001	SRR13386660	Illumina NovaSeq 6000	SampleAC5_ROC1_ST18_100ng_LIB3	PAIRED	bam	unmapped	17.45 GB	SampleAC5_ROC1_ST18_100ng_LIB3.bam							
Sample Spike-in	SAMN16786363	ROC1	SRX9806002	SRR13386659	Illumina NovaSeq 6000	SampleAC5_ROC1_ST18_100ng_LIB4	PAIRED	bam	unmapped	15.17 GB	SampleAC5_ROC1_ST18_100ng_LIB4.bam							
Sample Spike-in	SAMN16786363	ROC1	SRX9805972	SRR13386690	Illumina NovaSeq 6000	SampleAC5_ROC1_ST19_100ng_LIB1	PAIRED	bam	unmapped	22.79 GB	SampleAC5_ROC1_ST19_100ng_LIB1.bam							
Sample Spike-in	SAMN16786363	ROC1	SRX9805973	SRR13386689	Illumina NovaSeq 6000	SampleAC5_ROC1_ST19_100ng_LIB2	PAIRED	bam	unmapped	21.80 GB	SampleAC5_ROC1_ST19_100ng_LIB2.bam							
Sample Spike-in	SAMN16786363	ROC1	SRX9805975	SRR13386687	Illumina NovaSeq 6000	SampleAC5_ROC1_ST19_100ng_LIB3	PAIRED	bam	unmapped	23.40 GB	SampleAC5_ROC1_ST19_100ng_LIB3.bam							
Sample Spike-in	SAMN16786363	ROC1	SRX9805976	SRR13386686	Illumina NovaSeq 6000	SampleAC5_ROC1_ST19_100ng_LIB4	PAIRED	bam	unmapped	23.53 GB	SampleAC5_ROC1_ST19_100ng_LIB4.bam							
Sample B	SAMN16786361	ROC1	SRX9805939	SRR13386723	Illumina NovaSeq 6000	SampleB_ROC1_ST16_100ng_LIB1	PAIRED	bam	unmapped	21.20 GB	SampleB_ROC1_ST16_100ng_LIB1.bam							
Sample B	SAMN16786361	ROC1	SRX9805940	SRR13386722	Illumina NovaSeq 6000	SampleB_ROC1_ST16_100ng_LIB2	PAIRED	bam	unmapped	6.39 GB	SampleB_ROC1_ST16_100ng_LIB2.bam							
Sample B	SAMN16786361	ROC1	SRX9805941	SRR13386721	Illumina NovaSeq 6000	SampleB_ROC1_ST16_100ng_LIB3	PAIRED	bam	unmapped	15.17 GB	SampleB_ROC1_ST16_100ng_LIB3.bam							
Sample B	SAMN16786361	ROC1	SRX9805942	SRR13386720	Illumina NovaSeq 6000	SampleB_ROC1_ST16_100ng_LIB4	PAIRED	bam	unmapped	19.70 GB	SampleB_ROC1_ST16_100ng_LIB4.bam							
Sample B	SAMN16786361	ROC1	SRX9805961	SRR13386701	Illumina NovaSeq 6000	SampleB_ROC1_ST17_100ng_LIB1	PAIRED	bam	unmapped	19.04 GB	SampleB_ROC1_ST17_100ng_LIB1.bam							
Sample B	SAMN16786361	ROC1	SRX9805962	SRR13386700	Illumina NovaSeq 6000	SampleB_ROC1_ST17_100ng_LIB2	PAIRED	bam	unmapped	21.30 GB	SampleB_ROC1_ST17_100ng_LIB2.bam							
Sample B	SAMN16786361	ROC1	SRX9805963	SRR13386699	Illumina NovaSeq 6000	SampleB_ROC1_ST17_100ng_LIB3	PAIRED	bam	unmapped	30.77 GB	SampleB_ROC1_ST17_100ng_LIB3.bam							
Sample B	SAMN16786361	ROC1	SRX9805964	SRR13386698	Illumina NovaSeq 6000	SampleB_ROC1_ST17_100ng_LIB4	PAIRED	bam	unmapped	22.09 GB	SampleB_ROC1_ST17_100ng_LIB4.bam							
Sample B	SAMN16786361	ROC1	SRX9806003	SRR13386658	Illumina NovaSeq 6000	SampleB_ROC1_ST18_100ng_LIB1	PAIRED	bam	unmapped	16.06 GB	SampleB_ROC1_ST18_100ng_LIB1.bam							
Sample B	SAMN16786361	ROC1	SRX9806004	SRR13386657	Illumina NovaSeq 6000	SampleB_ROC1_ST18_100ng_LIB2	PAIRED	bam	unmapped	15.50 GB	SampleB_ROC1_ST18_100ng_LIB2.bam							
Sample B	SAMN16786361	ROC1	SRX9806005	SRR13386656	Illumina NovaSeq 6000	SampleB_ROC1_ST18_100ng_LIB3	PAIRED	bam	unmapped	16.96 GB	SampleB_ROC1_ST18_100ng_LIB3.bam							
Sample B	SAMN16786361	ROC1	SRX9806006	SRR13386655	Illumina NovaSeq 6000	SampleB_ROC1_ST18_100ng_LIB4	PAIRED	bam	unmapped	16.48 GB	SampleB_ROC1_ST18_100ng_LIB4.bam							
Sample B	SAMN16786361	ROC1	SRX9805977	SRR13386685	Illumina NovaSeq 6000	SampleB_ROC1_ST19_100ng_LIB1	PAIRED	bam	unmapped	19.70 GB	SampleB_ROC1_ST19_100ng_LIB1.bam							
Sample B	SAMN16786361	ROC1	SRX9805978	SRR13386684	Illumina NovaSeq 6000	SampleB_ROC1_ST19_100ng_LIB2	PAIRED	bam	unmapped	16.64 GB	SampleB_ROC1_ST19_100ng_LIB2.bam							
Sample B	SAMN16786361	ROC1	SRX9805979	SRR13386683	Illumina NovaSeq 6000	SampleB_ROC1_ST19_100ng_LIB3	PAIRED	bam	unmapped	20.06 GB	SampleB_ROC1_ST19_100ng_LIB3.bam							
Sample B	SAMN16786361	ROC1	SRX9805980	SRR13386682	Illumina NovaSeq 6000	SampleB_ROC1_ST19_100ng_LIB4	PAIRED	bam	unmapped	19.29 GB	SampleB_ROC1_ST19_100ng_LIB4.bam							
Sample C	SAMN16786362	ROC1	SRX9805943	SRR13386719	Illumina NovaSeq 6000	SampleC_ROC1_ST16_100ng_LIB1	PAIRED	bam	unmapped	34.84 GB	SampleC_ROC1_ST16_100ng_LIB1.bam							
Sample C	SAMN16786362	ROC1	SRX9805944	SRR13386718	Illumina NovaSeq 6000	SampleC_ROC1_ST16_100ng_LIB2	PAIRED	bam	unmapped	37.98 GB	SampleC_ROC1_ST16_100ng_LIB2.bam							
Sample C	SAMN16786362	ROC1	SRX9805945	SRR13386717	Illumina NovaSeq 6000	SampleC_ROC1_ST16_100ng_LIB3	PAIRED	bam	unmapped	46.57 GB	SampleC_ROC1_ST16_100ng_LIB3.bam							
Sample C	SAMN16786362	ROC1	SRX9805946	SRR13386716	Illumina NovaSeq 6000	SampleC_ROC1_ST16_100ng_LIB4	PAIRED	bam	unmapped	36.29 GB	SampleC_ROC1_ST16_100ng_LIB4.bam							
Sample C	SAMN16786362	ROC1	SRX9805965	SRR13386697	Illumina NovaSeq 6000	SampleC_ROC1_ST17_100ng_LIB1	PAIRED	bam	unmapped	34.04 GB	SampleC_ROC1_ST17_100ng_LIB1.bam							
Sample C	SAMN16786362	ROC1	SRX9805966	SRR13386676	Illumina NovaSeq 6000	SampleC_ROC1_ST17_100ng_LIB2	PAIRED	bam	unmapped	33.53 GB	SampleC_ROC1_ST17_100ng_LIB2.bam							
Sample C	SAMN16786362	ROC1	SRX9805987	SRR13386674	Illumina NovaSeq 6000	SampleC_ROC1_ST17_100ng_LIB3	PAIRED	bam	unmapped	58.64 GB	SampleC_ROC1_ST17_100ng_LIB3.bam							
Sample C	SAMN16786362	ROC1	SRX9805988	SRR13386673	Illumina NovaSeq 6000	SampleC_ROC1_ST17_100ng_LIB4	PAIRED	bam	unmapped	51.50 GB	SampleC_ROC1_ST17_100ng_LIB4.bam							
Sample C	SAMN16786362	ROC1	SRX9806007	SRR13386654	Illumina NovaSeq 6000	SampleC_ROC1_ST18_100ng_LIB1	PAIRED	bam	unmapped	37.12 GB	SampleC_ROC1_ST18_100ng_LIB1.bam							
Sample C	SAMN16786362	ROC1	SRX9806008	SRR13386653	Illumina NovaSeq 6000	SampleC_ROC1_ST18_100ng_LIB2	PAIRED	bam	unmapped	34.42 GB	SampleC_ROC1_ST18_100ng_LIB2.bam							
Sample C	SAMN16786362	ROC1	SRX9806009	SRR13386652	Illumina NovaSeq 6000	SampleC_ROC1_ST18_100ng_LIB3	PAIRED	bam	unmapped	35.99 GB	SampleC_ROC1_ST18_100ng_LIB3.bam							
Sample C	SAMN16786362	ROC1	SRX9806010	SRR13386651	Illumina NovaSeq 6000	SampleC_ROC1_ST18_100ng_LIB4	PAIRED	bam	unmapped	33.74 GB	SampleC_ROC1_ST18_100ng_LIB4.bam							
Sample C	SAMN16786362	ROC1	SRX9805981	SRR13386681	Illumina NovaSeq 6000	SampleC_ROC1_ST19_100ng_LIB1	PAIRED	bam	unmapped	37.94 GB	SampleC_ROC1_ST19_100ng_LIB1.bam							
Sample C	SAMN16786362	ROC1	SRX9805982	SRR13386680	Illumina NovaSeq 6000	SampleC_ROC1_ST19_100ng_LIB2	PAIRED	bam	unmapped	32.24 GB	SampleC_ROC1_ST19_100ng_LIB2.bam							
Sample C	SAMN16786362	ROC1	SRX9805983	SRR13386679	Illumina NovaSeq 6000	SampleC_ROC1_ST19_100ng_LIB3	PAIRED	bam	unmapped	39.05 GB	SampleC_ROC1_ST19_100ng_LIB3.bam							
Sample C	SAMN16786362	ROC1	SRX9805984	SRR13386678	Illumina NovaSeq 6000	SampleC_ROC1_ST19_100ng_LIB4	PAIRED	bam	unmapped	41.11 GB	SampleC_ROC1_ST19_100ng_LIB4.bam							
Sample A	SAMN16786360	TFS1	SRX9607679	SRR13169358	Ion Torrent S5 XL	SampleA_TFS1_ST22_20ng_LIB1	SINGLE	bam	GRCh37/hg19	0.71 GB	SampleA_TFS1_ST22_20ng_LIB1.bam							
Sample A	SAMN16786360	TFS1	SRX9607680	SRR13169357	Ion Torrent S5 XL	SampleA_TFS1_ST22_20ng_LIB2	SINGLE	bam	GRCh37/hg19	0.57 GB	SampleA_TFS1_ST22_20ng_LIB2.bam							
Sample A	SAMN16786360	TFS1	SRX9607681	SRR13169356	Ion Torrent S5 XL	SampleA_TFS1_ST22_20ng_LIB3	SINGLE	bam	GRCh37/hg19	0.73 GB	SampleA_TFS1_ST22_20ng_LIB3.bam							
Sample A	SAMN16786360	TFS1	SRX9607682	SRR13169355	Ion Torrent S5 XL	SampleA_TFS1_ST22_20ng_LIB4	SINGLE	bam	GRCh37/hg19	0.77 GB	SampleA_TFS1_ST22_20ng_LIB4.bam							
Sample A	SAMN16786360	TFS1	SRX9607684	SRR13169353	Ion Torrent S5 XL	SampleA_TFS1_ST23_20ng_LIB1	SINGLE	bam	GRCh37/hg19	0.66 GB	SampleA_TFS1_ST23_20ng_LIB1.bam							
Sample A	SAMN16786360	TFS1	SRX9607685	SRR13169352	Ion Torrent S5 XL	SampleA_TFS1_ST23_20ng_LIB2	SINGLE	bam	GRCh37/hg19	0.65 GB	SampleA_TFS1_ST23_20ng_LIB2.bam							
Sample A	SAMN16786360	TFS1	SRX9607686	SRR13169351	Ion Torrent S5 XL	SampleA_TFS1_ST23_20ng_LIB3	SINGLE	bam	GRCh37/hg19	0.63 GB	SampleA_TFS1_ST23_20ng_LIB3.bam							
Sample A	SAMN16786360	TFS1	SRX9607687	SRR13169350	Ion Torrent S5 XL	SampleA_TFS1_ST23_20ng_LIB4	SINGLE	bam	GRCh37/hg19	0.69 GB	SampleA_TFS1_ST23_20ng_LIB4.bam							
Sample A	SAMN16786360	TFS1	SRX9607688	SRR13169349	Ion Torrent S5 XL	SampleA_TFS1_ST24_20ng_LIB1	SINGLE	bam	GRCh37/hg19	0.55 GB	SampleA_TFS1_ST24_20ng_LIB1.bam							
Sample A	SAMN16786360	TFS1	SRX9607689	SRR13169348	Ion Torrent S5 XL	SampleA_TFS1_ST24_20ng_LIB2	SINGLE	bam	GRCh37/hg19	0.62 GB	SampleA_TFS1_ST24_20ng_LIB2.bam							
Sample A	SAMN16786360	TFS1	SRX9607690	SRR13169347	Ion Torrent S5 XL	SampleA_TFS1_ST24_20ng_LIB3	SINGLE	bam	GRCh37/hg19	0.56 GB	SampleA_TFS1_ST24_20ng_LIB3.bam							
Sample A	SAMN16786360	TFS1	SRX9607691	SRR13169346	Ion Torrent S5 XL	SampleA_TFS1_ST24_20ng_LIB4	SINGLE	bam	GRCh37/hg19	0.66 GB	SampleA_TFS1_ST24_20ng_LIB4.bam							
Sample Spike-in	SAMN16786363	TFS1	SRX9607692	SRR13169345	Ion Torrent S5 XL	SampleAC5_TFS1_ST22_20ng_LIB1	SINGLE	bam	GRCh37/hg19	0.70 GB	SampleAC5_TFS1_ST22_20ng_LIB1.bam							
Sample Spike-in	SAMN16786363	TFS1	SRX9607693	SRR13169344	Ion Torrent S5 XL	SampleAC5_TFS1_ST22_20ng_LIB2	SINGLE	bam	GRCh37/hg19	0.66 GB	SampleAC5_TFS1_ST22_20ng_LIB2.bam							
Sample Spike-in	SAMN16786363	TFS1	SRX9607695	SRR13169342	Ion Torrent S5 XL	SampleAC5_TFS1_ST22_20ng_LIB3	SINGLE	bam	GRCh37/hg19	0.83 GB	SampleAC5_TFS1_ST22_20ng_LIB3.bam							
Sample Spike-in	SAMN16786363	TFS1	SRX9607696	SRR13169341	Ion Torrent S5 XL	SampleAC5_TFS1_ST22_20ng_LIB4	SINGLE	bam	GRCh37/hg19	0.68 GB	SampleAC5_TFS1_ST22_20ng_LIB4.bam							
Sample Spike-in	SAMN16786363	TFS1	SRX9607697	SRR13169340	Ion Torrent S5 XL	SampleAC5_TFS1_ST23_20ng_LIB1	SINGLE	bam	GRCh37/hg19	0.69 GB	SampleAC5_TFS1_ST23_20ng_LIB1.bam							
Sample Spike-in	SAMN16786363	TFS1	SRX9607698	SRR13169339	Ion Torrent S5 XL	SampleAC5_TFS1_ST23_20ng_LIB2	SINGLE	bam	GRCh37/hg19	0.69 GB	SampleAC5_TFS1_ST23_20ng_LIB2.bam							
Sample Spike-in	SAMN16786363	TFS1	SRX9607699	SRR13169338	Ion Torrent S5 XL	SampleAC5_TFS1_ST23_20ng_LIB3	SINGLE	bam	GRCh37/hg19	0.74 GB	SampleAC5_TFS1_ST23_20ng_LIB3.bam							
Sample Spike-in	SAMN16786363	TFS1	SRX9607700	SRR13169337	Ion Torrent S5 XL	SampleAC5_TFS1_ST23_20ng_LIB4	SINGLE	bam	GRCh37/hg19	0.70 GB	SampleAC5_TFS1_ST23_20ng_LIB4.bam							
Sample Spike-in	SAMN16786363	TFS1	SRX9607701	SRR13169336	Ion Torrent S5 XL	SampleAC5_TFS1_ST24_20ng_LIB1	SINGLE	bam	GRCh37/hg19	63 GB	SampleAC5_TFS1_ST24_20ng_LIB1.bam							
Sample Spike-in	SAMN16786363	TFS1	SRX9607702	SRR13169335	Ion Torrent S5 XL	SampleAC5_TFS1_ST24_20ng_LIB2	SINGLE	bam	GRCh37/hg19	0.64 GB	SampleAC5_TFS1_ST24_20ng_LIB2.bam							
Sample Spike-in	SAMN16786363	TFS1	SRX9607703	SRR13169334	Ion Torrent S5 XL	SampleAC5_TFS1_ST24_20ng_LIB3	SINGLE	bam	GRCh37/hg19	0.75 GB	SampleAC5_TFS1_ST24_20ng_LIB3.bam							
Sample Spike-in	SAMN16786363	TFS1	SRX9607704	SRR13169333	Ion Torrent S5 XL	SampleAC5_TFS1_ST24_20ng_LIB4	SINGLE	bam	GRCh37/hg19	0.67 GB	SampleAC5_TFS1_ST24_20ng_LIB4.bam							
Sample B	SAMN16786361	TFS1	SRX9607706	SRR13169331	Ion Torrent S5 XL	SampleB_TFS1_ST22_20ng_LIB1	SINGLE	bam	GRCh37/hg19	0.66 GB	SampleB_TFS1_ST22_20ng_LIB1.bam							
Sample B	SAMN16786361	TFS1	SRX9607707	SRR13169330	Ion Torrent S5 XL	SampleB_TFS1_ST22_20ng_LIB2	SINGLE	bam	GRCh37/hg19	0.69 GB	SampleB_TFS1_ST22_20ng_LIB2.bam							
Sample B	SAMN16786361	TFS1	SRX9607708	SRR13169329	Ion Torrent S5 XL	SampleB_TFS1_ST22_20ng_LIB3	SINGLE	bam	GRCh37/hg19	0.66 GB	SampleB_TFS1_ST22_20ng_LIB3.bam							
Sample B	SAMN16786361	TFS1	SRX9607709	SRR13169328	Ion Torrent S5 XL	SampleB_TFS1_ST22_20ng_LIB4	SINGLE	bam	GRCh37/hg19	0.58 GB	SampleB_TFS1_ST22_20ng_LIB4.bam							
Sample B	SAMN16786361	TFS1	SRX9607710	SRR13169327	Ion Torrent S5 XL	SampleB_TFS1_ST23_20ng_LIB1	SINGLE	bam	GRCh37/hg19	0.59 GB	SampleB_TFS1_ST23_20ng_LIB1.bam							
Sample B	SAMN16786361	TFS1	SRX9607711	SRR13169326	Ion Torrent S5 XL	SampleB_TFS1_ST23_20ng_LIB2	SINGLE	bam	GRCh37/hg19	0.70 GB	SampleB_TFS1_ST23_20ng_LIB2.bam							
Sample B	SAMN16786361	TFS1	SRX9607712	SRR13169325	Ion Torrent S5 XL	SampleB_TFS1_ST23_20ng_LIB3	SINGLE	bam	GRCh37/hg19	0.70 GB	SampleB_TFS1_ST23_20ng_LIB3.bam							
Sample B	SAMN16786361	TFS1	SRX9607713	SRR13169324	Ion Torrent S5 XL	SampleB_TFS1_ST23_20ng_LIB4	SINGLE	bam	GRCh37/hg19	0.70 GB	SampleB_TFS1_ST23_20ng_LIB4.bam							
Sample B	SAMN16786361	TFS1	SRX9607714	SRR13169323	Ion Torrent S5 XL	SampleB_TFS1_ST24_20ng_LIB1	SINGLE	bam	GRCh37/hg19	0.55 GB	SampleB_TFS1_ST24_20ng_LIB1.bam							
Sample B	SAMN16786361	TFS1	SRX9607715	SRR13169322	Ion Torrent S5 XL	SampleB_TFS1_ST24_20ng_LIB2	SINGLE	bam	GRCh37/hg19	0.64 GB	SampleB_TFS1_ST24_20ng_LIB2.bam							
Sample B	SAMN16786361	TFS1	SRX9607717	SRR13169320	Ion Torrent S5 XL	SampleB_TFS1_ST24_20ng_LIB3	SINGLE	bam	GRCh37/hg19	0.62 GB	SampleB_TFS1_ST24_20ng_LIB3.bam							
Sample B	SAMN16786361	TFS1	SRX9607718	SRR13169319	Ion Torrent S5 XL	SampleB_TFS1_ST24_20ng_LIB4	SINGLE	bam	GRCh37/hg19	0.53 GB	SampleB_TFS1_ST24_20ng_LIB4.bam							
